# Decreased tryptophan metabolism in patients with autism spectrum disorders

**DOI:** 10.1186/2040-2392-4-16

**Published:** 2013-06-03

**Authors:** Luigi Boccuto, Chin-Fu Chen, Ayla R Pittman, Cindy D Skinner, Heather J McCartney, Kelly Jones, Barry R Bochner, Roger E Stevenson, Charles E Schwartz

**Affiliations:** 1 Greenwood Genetic Center, 113 Gregor Mendel Circle, Greenwood, SC 29646, USA; 2 Biolog, Inc., 21124 Cabot Boulevard, Hayward, CA, 94545, USA

**Keywords:** Autism, Biomarker, Tryptophan, Metabolism, Screening

## Abstract

**Background:**

Autism spectrum disorders (ASDs) are relatively common neurodevelopmental conditions whose biological basis has been incompletely determined. Several biochemical markers have been associated with ASDs, but there is still no laboratory test for these conditions.

**Methods:**

We analyzed the metabolic profile of lymphoblastoid cell lines from 137 patients with neurodevelopmental disorders with or without ASDs and 78 normal individuals, using Biolog Phenotype MicroArrays.

**Results:**

Metabolic profiling of lymphoblastoid cells revealed that the 87 patients with ASD as a clinical feature, as compared to the 78 controls, exhibited on average reduced generation of NADH when tryptophan was the sole energy source. The results correlated with the behavioral traits associated with either syndromal or non-syndromal autism, independent of the genetic background of the individual. The low level of NADH generation in the presence of tryptophan was not observed in cell lines from non-ASD patients with intellectual disability, schizophrenia or conditions exhibiting several similarities with syndromal autism except for the behavioral traits. Analysis of a previous small gene expression study found abnormal levels for some genes involved in tryptophan metabolic pathways in 10 patients.

**Conclusions:**

Tryptophan is a precursor of important compounds, such as serotonin, quinolinic acid, and kynurenic acid, which are involved in neurodevelopment and synaptogenesis. In addition, quinolinic acid is the structural precursor of NAD^+^, a critical energy carrier in mitochondria. Also, the serotonin branch of the tryptophan metabolic pathway generates NADH. Lastly, the levels of quinolinic and kynurenic acid are strongly influenced by the activity of the immune system. Therefore, decreased tryptophan metabolism may alter brain development, neuroimmune activity and mitochondrial function. Our finding of decreased tryptophan metabolism appears to provide a unifying biochemical basis for ASDs and perhaps an initial step in the development of a diagnostic assay for ASDs.

## Background

Autism spectrum disorders (ASDs) constitute a heterogeneous group of neurodevelopmental conditions characterized by three core signs: impairment of social interactions, communication issues, and repetitive behaviors. The prevalence of ASDs is approximately 1% in the US population aged <8 years [1] and the conditions have a tremendous impact on society and families as affected individuals utilize a wide range of services and considerable resources [2]. Currently, ASDs are diagnosed solely by analysis of the complex behavioral phenotype and such diagnosis is typically not performed before the age of 3 years, even if recent progress has allowed diagnosis at an earlier age [3]. A strong genetic predisposition for ASDs has been inferred from a higher concordance in identical twins as compared to fraternal twins, a significant sib recurrence risk, and the identification of a predisposing genetic event in 15% to 30% of ASD children [4,5]. Nevertheless, ASDs exhibit a remarkable genetic heterogeneity and a pathogenic hypothesis encompassing all the reported candidate genes is difficult to postulate.

The investigation for the molecular bases of these disorders has highlighted numerous metabolic abnormalities in ASD patients and several biochemical markers have been associated with autistic traits (hyperserotoninemia [6], urinary catabolites of sulfur and amino acid metabolism [7], and oxidative metabolism biomarkers [8]). However, there are still no laboratory tests that offer a reliable confirmation for the clinical diagnosis, or provide for efficient screening of individuals presenting with behavioral features suggestive of ASDs.

## Methods

### Patient and control cell lines

We analyzed 87 ASD patients, 80 patients with non-syndromal ASDs and seven patients with syndromal ASDs. Overall, 74 ASD patients were male and 13 were female, a 5.69:1 ratio. The age range was 2.5 to 34.25 years at the time the blood sample was obtained. The non-syndromal patients were part of the South Carolina Autism Project (SCAP) and were diagnosed with autistic disorder based on evaluation using the Autism Diagnostic Interview-Revised (ADI-R) and according to the Diagnostic and Statistical Manual of Mental Disorders (DSM) IV-Revised criteria [9]. Genetic tests excluded major chromosomal abnormalities, Fragile X syndrome, Rett syndrome (by testing the *MECP2* gene), and abnormalities in plasma amino acid levels. The seven syndromal patients were diagnosed with ASDs based on evaluation using the ADI-R and the Autism Diagnostic Observation Schedule (ADOS), according to the DSM IV-Revised criteria. Two cell lines from patients with Fragile X syndrome were provided from the Catholic University of Sacred Heart, Rome, Italy. We also analyzed 35 patients with 15 different intellectual disability (ID) conditions who were known to have mutations in ID-associated genes, and 10 patients with schizophrenia, provided by the Ste-Justine Hospital Research Center, University of Montreal, Canada. We compared these various patients with 78 controls, who were matched by age (range, 4.17-53.5 years at blood sampling), sex (66 males and 12 females, ratio 5.2), and geographic area.

Informed consent was approved by the Self Regional Healthcare Institutional Review Board (IRB) for Human Research and was reviewed and signed by all the participants evaluated and/or their legal guardian. The IRB approved the use of the samples in the studies reported in this paper.

Cell lines were obtained by immortalization of lymphocytes from blood samples using Epstein-Barr virus. The lymphoblastoid cell lines were harvested in Sigma RPMI-1640 with 75 mL fetal bovine serum from Atlanta Biological (Lawrenceville, GA, USA) and 5 mL L-Glutamine and 5 mL antibiotic/antimycotic from Sigma-Aldrich (St. Louis, MO, USA).

### Biolog metabolic arrays

The Phenotype MicroArray (PM) developed by Biolog (Hayward, CA, USA) is designed to provide an unbiased scan of the activity of cellular metabolic pathways involved in the rate of production of NADH (nicotinamide adenine dinucleotide, reduced form). The methodology employs microplates with diverse carbon energy sources (plate PM-M1), as well as amino acids, both alone and as dipeptides (plates PM-M2 to M4). Each well contains a single chemical as the sole energy source and production of NADH per well is monitored using a colorimetric redox dye chemistry [10]. PM-M plates were incubated with 20,000 lymphoblastoid cells per well in a volume of 50 μL. The cells were incubated for 48 h at 37°C in 5% CO_2_, using the modified Biolog IF-M1 medium. This medium was prepared by adding the following to 100 mL of Biolog IF-M1: 1.1 mL of 100× penicillin/streptomycin solution, 0.16 mL of 200 mM Glutamine (final concentration 0.3 mM), and 5.3 mL of fetal bovine serum (final concentration 5%). During the 48-h incubation, the only energy source the cells had was the chemical in the well. After this first incubation, Biolog Redox Dye Mix MB was added (10 μL/well) and the plates were incubated under the same conditions for an additional 24 h, during which time the cells metabolize the sole carbon source in the well. As the cells metabolize the carbon source, tetrazolium dye in the media is reduced, producing a purple color according to the amount of NADH generated. For further details on the Biolog Phenotype MicroArray see Bochner *et al.*[10] and Putluri *et al*. [11].

At the end of the 24 h incubation, the plates were analyzed utilizing a microplate reader with readings at 590 and 750 nm. The first value (A_590_) indicated the highest absorbance peak of the redox dye and the second value (A_750_) gave a measure of the background noise. The relative absorbance (A_590-750_) was calculated per well.

For the last experiment of 50 ASD patients *versus* 50 controls, we used the customized Biolog plates described below following the same protocol described above.

### Customized metabolic plates

Customized plates were designed by Biolog for the 50 ASD patients *versus* 50 controls experiment. The wells contained a positive control (α-D-glucose), a negative control (empty well), L-tryptophan, and the five most informative tryptophan dipeptides (Trp-Gly, Trp-Lys, Trp-Ala, Trp-Arg, and Trp-Leu).

### Human gene expression microarrays

The Agilent Whole Human Genome Oligo Microarray, containing over 40,000 human genes and transcripts [12], was utilized to evaluate gene expression levels in cell lines from 10 patients with ASDs and 10 controls. Two independent microarray experiments were performed for each sample. To maximize the contrast between samples, we implemented a loop experimental design [13] with dye swap. Total RNA was isolated using the Agilent Total RNA Isolation Mini Kit (Agilent Technologies, Wilmington, DE, USA). RNA samples were labeled with Cy3 or Cy5 fluorescent dye and hybridized for 17 h, and microarray slides were washed and scanned with Agilent G2505B microarray scanner. Image processing and fluorescence intensity were interpreted and analyzed by Agilent Feature Extraction (version 9.1).

### Data processing and statistical analysis

#### Phenotype MicroArray (PM) analysis

For PM data, the absorbance readings were transformed to a logarithmic scale. We assumed that as a group, patients with ASDs share a similar metabolic profile. At the first stage, as an exploratory approach, our goal was to identify the wells in which ASD patients were significantly different from the controls. We utilized a popular statistical program, Significance Analysis of Microarray (SAM) [14], which implements a modified *t* test by assigning a score to each observation on the basis of change and utilizes permutations to estimate the percentage of observations identified by chance using a false discovery rate (FDR). FDR maintains a balance between the number of false positives [15] and true positives and is equivalent to the family-wise error but with a gain in power. We used FDR at 1% as the selection threshold. At the second stage, after selecting most of the tryptophan-containing wells as the main focus, a *t* test with Bonferroni correction was used for determination of statistical significance.

#### Feature selection

In order to determine whether each tryptophan dipeptide possesses the same statistical power in distinguishing patients from controls, we utilized 10 feature selection methods. Since each feature selection method contains a bias, we adopted a ‘voting’ strategy to look for ‘features’ (that is, tryptophan substrates) selected by the majority of the algorithms. We utilized 10 feature selection methods (CfsSubsetEval, ChiSquared, FilteredAttribute, FilteredSubset, GainRatioAttribute, InfoGainAttribute, OneRAttribute, ReliefFAttribute, SVMAttribute, SymmetricalUncertAttribute) in the Weka package [16] and had each method rank and report the best top five discriminators.

#### Expression microarray analysis

For the Agilent expression arrays, data normalization and statistical analysis were performed as in the reference [17].

## Results

### Identification of reduced tryptophan metabolism in the ASD cells

We initiated an investigation of the metabolic profile of lymphoblastoid cell lines from patients with different neurobehavioral disorders and normal individuals, utilizing Biolog Phenotype MicroArray plates to see if patients with specific disorders had a characteristic profile. The assay, as carried out, analyzes the NADH generation in the presence of each substrate by measuring the redox color change of a tetrazolium dye after 24 h of incubation.

We first tested the ability of lymphoblastoid cells to utilize the 367 substrates in the PM plates in 18 controls and 55 patients. Seventeen of these patients were affected with ASDs and 38 had 15 different intellectual disability (ID) conditions and known pathogenic mutations in specific ID-associated genes. Of the 17 ASD patients, 10 had non-syndromal autistic disorder with known genetic alterations: two of these patients were monozygotic male twins with a chromosome 3p26.2 deletion, not involving any reported gene, one had a chromosome 2p21 duplication and a chromosome 20p21.1 deletion, three had *SHANK3* mutations, two had *NLGN4* mutations, and one had a balanced translocation involving chromosomes 14 and 15. The remaining seven cell lines were from patients with syndromal autism: four cases had Fragile X syndrome, autistic features and full methylation at the *FRAXA* locus; two cases had Rett syndrome and *MECP2* mutations; one case had a *ZNF711* mutation, X-linked intellectual disability and clinical findings consistent with ASDs (see Additional file 1: Table S1 for more detailed description).

The analysis of the Biolog PM plate data was conducted in a blind fashion so that one of us (CFC) was ignorant of the clinical information connected to each patient. The analysis of the assay data revealed significant differences in the utilization of multiple substrates as an energy source for most patient cell lines when they were compared to the controls. However, once the clinical information was attached to the data, if an ASD was present, it was noted that the 25 substrates with the greatest statistical significance (*P* value ≤0.001) contained a form of tryptophan, and all 27 wells containing tryptophan had *P* values ≤0.05 (Additional file 2: Table S2). This collective finding was independent of whether tryptophan was alone in the well or in the first or second position of a dipeptide. These observations suggested that lymphoblastoid cell lines from either syndromal or non-syndromal ASD patients exhibit reduced tryptophan metabolism as compared to normal individuals. Among the top 50 wells with a significant difference between patients without ASDs and controls, none contained tryptophan (Additional file 3: Table S3). Out of 222 substrates that met the minimal statistical threshold for significance (*P* <0.05), only two (0.9%) contained tryptophan (Lys-Trp and Pro-Trp) for patients without ASDs. The results suggest that tryptophan metabolism in lymphoblast cells from non-ASD patients with neurodevelopmental disorders is not significantly different as compared to normal individuals.

It is noteworthy that some of these patients were affected by conditions that sometimes may present with autistic traits: (1) two cell lines were from patients with Angelman syndrome, a condition often considered in the differential diagnosis of Rett syndrome, who did not meet criteria for ASDs; and (2) a patient with a *ZNF711* mutation who did not exhibit ASD features.

In order to evaluate the specificity of our finding with regard to the behavioral phenotype associated with ASDs, we tested 10 patients with schizophrenia, a condition that shares some features with ASDs. We did not detect significant abnormalities in tryptophan metabolism, as compared to controls (data not shown).

In summary, these data suggested that the reduced tryptophan metabolism: (1) was a prominent feature common for the 17 ASD patient cell lines tested, as a group, independent of whether the autistic traits were the sole clinical findings in the patients or whether they were accompanied by other signs in a syndromal condition; (2) was extremely rare in patients with similar conditions but without features consistent with ASDs; and (3) was not observed in other neurodevelopmental conditions.

### Validation studies and feature selection

**Figure 1:**
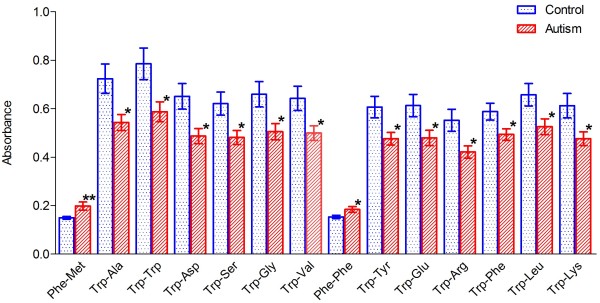
**Histogram of energy metabolism in the ASD cells *vs*. control cells. **The bar height of the histogram indicates the mean of measurements of 20 cell lines for each group; vertical bars are standard errors of the means. Star* symbol denotes level of statistical significance determined by a *t* test: **P <0.01; *P <0.05. The substrates were ordered according to their individual *P *values with the lowest on the left.

In order to replicate our initial results, we tested an additional 20 patients with autistic disorders, according to the DSM IV-Revised criteria, 10 new controls and 10 of the previously tested controls randomly selected. Thirteen of the 20 ASD patients had genetic abnormalities: seven patients had the *MET* rs1858830 C/C genotype, four had chromosomal rearrangements found by array-CGH analysis, one had a balanced translocation involving chromosomes 13 and 15, and one had a mutation in the *OCRL1* gene. To focus our analysis on tryptophan metabolism, we utilized only the PM-M4 plate, which contains the 12 wells with tryptophan as the first amino acid in various dipeptides. The PM-M4 plate also contains 17 wells with tyrosine, an amino acid that shares some common pathways with tryptophan and whose utilization was significantly reduced in 19/27 wells (70.4%) in our previous cohort. The Biolog results in these ASD cell lines, on average, indicated that the tryptophan metabolism was reduced when compared to the controls (Figure 1 and Additional file 4: Table S4), which was consistent with our findings for the previously tested 17 ASD cell lines. No significant changes were detected for the wells containing tyrosine, except for the one with the tryptophan-tyrosine dipeptide (Additional file 4: Table S4).

We investigated whether each tryptophan dipeptide possesses the same statistical power in distinguishing patients from controls. Ten feature selection methods were utilized to analyze the data obtained from the experiments of 20 controls *vs.* 20 patients. The analysis revealed that the top tryptophan dipeptides in order of informative power were: tryptophan-glycine (Trp-Gly), tryptophan-lysine (Trp-Lys), tryptophan-alanine (Trp-Ala), tryptophan-arginine (Trp-Arg), and tryptophan-leucine (Trp-Leu).

**Figure 2:**
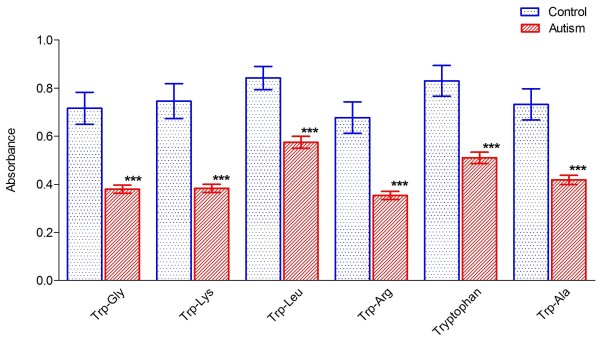
**Histogram of energy metabolism in the ASD cells *vs*. control cells. **The bar height of the histogram indicates the mean of measurements of 50 cell lines for each group; vertical bars are standard errors of the means. Star symbol (*) denotes level of statistical significance determined by a *t* test: ***P <0.001. The substrates were ordered according to their individual *P *values with the lowest on the left.

The results allowed us to design a customized 96-well plate with 12 columns of eight wells. These customized plates were used to test lymphoblastoid cell lines from new, unrelated cohorts of 50 ASD patients and 50 controls. This experiment, using five tryptophan dipeptides along with a positive control (α-D-glucose), a negative control (empty well), and L-tryptophan, replicated our previous observations of reduced tryptophan metabolism in lymphoblastoid cell lines from patients with ASDs (Figure 2). We also noticed that the positive control well containing glucose showed a difference between controls and patients with ASDs, even if not as significant as the one observed in the wells containing tryptophan. This observation suggested that there might exist some fundamental differences in the general energetic metabolism between ASD cases and controls, which were not observed in our previous data. To determine if the contribution of this possible general effect of energy utilization affected our results, we re-analyzed the data by ‘normalizing’ the tryptophan values with the positive control well. For each individual we have tested, we subtracted the value of each well containing tryptophan from the corresponding value of the well containing glucose. The result of this ‘normalization’ was that data still supported the previous observation that the lymphoblastoid cell lines of the patients with ASDs, on average, utilize tryptophan less effectively than those of controls (data not shown).

Since the experiment exploring the detected five tryptophan dipeptides utilized a larger cohort and more diverse group of samples, we tested whether age was a co-factor for the difference observed. A Spearman correlation analysis failed to find any age-related effect on the tryptophan utilization either in the patients with ASDs or in the controls (results not shown).

### Gene expression in the tryptophan metabolic pathway

**Figure 3:**
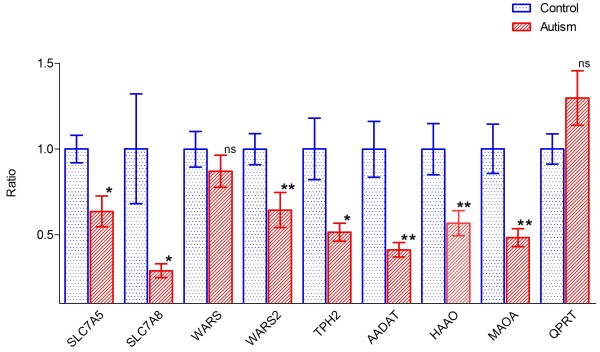
**Histogram of gene expression of selected genes in tryptophan metabolic pathway in the ASD cells *vs*. control cells. **The bar height of the histogram indicates the mean of measurements of 10 cell lines for the controls and 10 cell lines of patients with ASDs. Vertical bars are standard errors of the means. Symbol denotes level of statistical significance determined by a *t *test: *P <0.05; **P <0.01. ns, non-significant.

**Figure 4:**
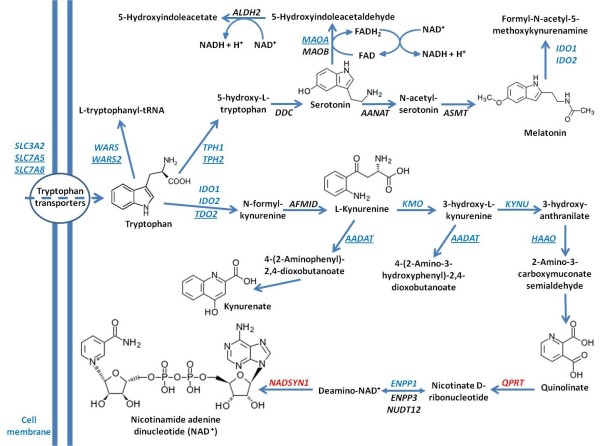
**Tryptophan pathways. **The figure illustrates the main intracellular pathways involving tryptophan. Genes with reduced expression in our microarray dataset are in blue, genes with increased expression are in red. Genes with statistically significant reduction of expression are underlined. Reactions generating NADH are indicated in the top section of the figure. FAD, flavin adenine dinucleotide.

It is reasonable to hypothesize that the observed reduction of tryptophan metabolism in ASD cell lines may stem from abnormal functions along tryptophan metabolic pathways in the cells. We therefore mined data generated from a small gene expression profiling study we had conducted previously to examine this possibility. The study employed the Agilent Whole Human Genome Oligo Microarrays and RNA extracted from the initial 10 ASD cell lines and 10 controls (Figure 3 and online). The data indicated that two genes, *SLC7A5* and *SLC7A8*, coding for tryptophan transporter subunits, expressed in both blood and brain, had reduced expression in all patients (Figures 3 and 4). The mitochondrial isoform of tryptophanyl tRNA synthetase (*WARS2*) had significantly reduced expression (*P* <0.001) in a majority (6/10) of the ASD cell lines while the cytoplasmic isoform (*WARS*) showed no difference in expression levels (Figures 3 and 4, Additional file 5: Table S5).

The two main pathways of tryptophan metabolism lead to the synthesis of serotonin and kynurenine [18]. Tryptophan hydroxylase is the rate-limiting enzyme in the biosynthesis of serotonin and the gene encoding the isoform 2 of this enzyme (*TPH2*) showed significantly reduced expression levels (Figures 3 and 4, Additional file 5: Table S5). Also, several genes coding for enzymes involved in the kynurenine pathway showed significant differences (*P* <0.05) between ASD cell lines and controls. The expression levels of *AADAT*, *HAAO*, and *MAOA* were reduced in patients with ASDs, while *QPRT* showed a non-significant trend towards over-expression (Figure 3 and Additional file 5: Table S5).

Not all genes in tryptophan related pathways exhibited significant expression differences between ASD patients and controls, and each patient exhibited a different profile for the group of genes examined. However, each of the 10 ASD patients showed significant differences (*P* <0.05) from controls in the expression levels of at least 9/15 genes involved in tryptophan metabolism (Additional file 5: Table S5).

## Discussion

Our findings show an abnormal utilization of tryptophan as energy source in cells from patients with ASDs, suggesting impaired tryptophan metabolism. Our analysis consisted of three independent sets of experiments in which we measured NADH production in 87 lymphoblastoid cells derived from ASD patients as compared to 78 controls. The difference between the case–control populations was statistically significant in each of these experiments. The statistical analysis was performed in a blind fashion with regard to the presence of ASDs in the patients. The results correlated with the behavioral traits associated with either syndromal or non-syndromal autism, independent of the genetic background of the individual. The low level of NADH generation in the presence of tryptophan was not observed in cell lines from patients with intellectual disability without ASD or schizophrenia, or in conditions showing several similarities with syndromal ASDs except for the behavioral traits.

Metabolism of tryptophan via the serotonin synthesis pathway leads to production of NADH, while metabolism via the kynurenine-quinolinic acid pathway leads to the synthesis of NAD^+^, the precursor of NADH (Figure 4). The decreased level of NADH generation in the presence of tryptophan may reflect less utilization of tryptophan resulting from downregulation of metabolic reactions along either of these pathways.

Analysis of microarray expression data previously collected on the initial 10 ASD patient cell lines and 10 controls found reduced levels of some genes involved in the serotonin and kynurenine pathways in ASD patients. No two patients exhibited the same expression profile. The enzyme responsible for the conversion of quinolinic acid to nicotinate D-ribonucleotide (quinolinate phosphoribosyltransferase) showed a trend towards increased expression, although this was statistically significant in only seven out of the 10 cell lines analyzed, when each cell line was individually compared to the control group using the Mann–Whitney one sample test (Additional file 5: Table S5). This enzyme links the tryptophan-kynurenine pathway to NAD^+^ biosynthesis (Figure 4) and is produced by a gene (*QPRT*) mapping to 16p11.2. Deletions and duplications of this region have been frequently associated with ASDs, suggesting that abnormal dosage of the genes in 16p11.2 may be responsible for autism features [19].

Pyridoxine and its metabolite, pyridoxal phosphate, play a critical role as co-factors in both the serotonin and kynurenine pathways, so their deficiency may affect tryptophan metabolism. Unfortunately, the PM plates do not contain any compound closely related to pyridoxine. Additionally, our limited microarray data for the enzymes involved in pyridoxine/pyridoxal phosphate metabolism (*PHOSPHO2*, *PDX*, *PNPO*) did not show significant differences when compared to controls in any of the 10 patients.

Our findings support a possible mitochondrial dysfunction as a result of impaired tryptophan metabolism in cells from patients with ASDs [20] and several biochemical mechanisms could be responsible. NADH is a critical energy carrier for the electron transport chain and tryptophan is the main precursor of the kynurenine-quinolinic acid pathway that leads to NAD^+^ synthesis. Furthermore, the expression microarray data from a previous study revealed selective under-expression of the mitochondrial isoform of tryptophanyl tRNA synthetase (*WARS2*) in 6/10 cell lines from ASD patients, and normal expression levels for the cytoplasmic isoform (*WARS1*). Considering the expression of tRNA genes is sensitive to the availability of the corresponding amino acid, it is possible the tryptophan levels in mitochondria are lower in ASD patients than controls (Figure 4).

In the brain, mitochondrial dysfunction has an effect on neuronal development and morphology, neurite overgrowth, and synaptic plasticity [20-22]. There appears to be a close link between mitochondrial dysfunction and synaptic abnormalities, which are considered one of the main pathogenic events associated with ASDs [5,23]. Five of the first 10 ASD cases had a mutation in genes involved in glutamatergic synapses (*SHANK3* and *NLGN4*). Also, some of the pathways involved in regulation of synaptic protein expression [24] are also involved in the expression of protein destined for the mitochondria.

The biochemical pathway that leads to NAD^+^ synthesis, beginning with tryptophan, is the kynurenine pathway (Figure 4) which has two major final products: quinolinic acid (metabolized to nicotinamide and NAD^+^), and kynurenic acid [18]. Some of the most frequently reported neuroanatomical findings in ASD brains are increased brain size and relatively increased white matter, particularly in the radiate zone [25,26]. It is possible that impairment of the quinolinic-kynurenic balance may have consequences on the programmed ‘pruning’ process and lead to an excess of white matter. Microglial cells are considered to be responsible for keeping the proper balance between quinolinic and kynurenic acid, since they are able to secrete both compounds. The expression of those molecules is strongly influenced by the activity of the immune system. Particularly, nitric oxide (NO) plays a critical role in the interaction between inflammation and neuronal circuits and causes mitochondrial dysfunction. It is important to note that elevated NO levels have been reported in ASD patients [27,28].

Although approximately 99% of the dietary tryptophan intake is metabolized via the kynurenine pathway [18], tryptophan is also the main precursor for both serotonin and melatonin (Figure 4). Melatonin plays a critical role in the regulation of the circadian rhythm, and anomalies of this rhythm have been associated with some of the signs in the autistic spectrum, like seizures or sleep disorders [23]. Serotonin is a neurotransmitter involved in multiple aspects of brain functions, ranging from the regulation of mood to the control of appetite and social interactions [29] and its production has been reported as deficient in ASD brains [30]. Tryptophan levels have been demonstrated to directly influence central nervous system (CNS) serotonin levels [31] and behavior [32], and altered tryptophan transport has been described in fibroblasts from boys with attention deficit/hyperactivity disorder (ADHD) [33]. It is noteworthy that tryptophan hydroxylase is the rate-limiting enzyme in the biosynthesis of serotonin and the gene coding the isoform 2 of this enzyme (*TPH2*) was underexpressed in the expression microarray analysis of lymphoblastoid cells from 10 ASD patients as compared to controls (*P* = 0.0166). In the human brain, both isoforms, *TPH1* and *TPH2*, are ubiquitously expressed with some particular region-specific differences [34] and *TPH2* expression is significantly higher in the raphe nuclei, the core of serotonergic system [35]. Thus, lower expression levels of *TPH2* in white cells might reflect abnormal serotonergic activity in the raphe nuclei. Additionally, the MAOA enzyme in the serotonin pathway which generates NADH was found to be underexpressed in cells from ASD patients.

Serotonin also plays a critical role in regulating neuronal morphology and circuitry [36] and recent work showed that placental cells are able to synthesize serotonin from tryptophan provided by the maternal blood [37]. This exogenous source of serotonin is important in the neurodevelopment of the forebrain in the first month of gestation, because the endogenous source (hindbrain, future serotonergic system) is not sufficient at this stage. Interestingly, disrupted organization of the fronto-temporal lobes is one of the most consistent neuroanatomical findings in ASD patients [25], and lower expression levels of genes involved in synapses, neurotransmitter transport and neuron projection have been detected in frontal and temporal lobes of ASD brains [38]. At the cellular level, such disrupted organization is reflected by abnormalities in the minicolumns, the basic functional units of the cortex, which have been reported in ASD brains to be narrower, increased in number per cortical area and with reduction of neuropil space, caused by the smaller size of the peripheral interneurons [25,39].

## Conclusions

The data presented in this work indicate that cells from patients with ASDs, on average, are less capable of utilizing tryptophan as an energy source than controls. The finding was consistent in both syndromal and non-syndromal cases, and was not influenced by age, sex, or genotype of the patients. We believe that decreased tryptophan metabolism in patients with ASDs may alter metabolic pathways involved in the regulation of the early stages of brain development (first month of gestation), mitochondrial homeostasis and immune system activity in the brain. Disruption of such pathways can primarily be caused either by insufficient serotonin production by placental cells, mitochondrial dysfunction and/or impaired balance between quinolinic and kynurenic acid in fetal cells. The combined effects of these events could lead to abnormal organization of neurons (minicolumnopathy), particularly in specific brain regions (fronto-temporal lobes, limbic system), determining the imbalance between the short- and long-term circuitry that has been considered to be one of the fundamentals of the ASD neuropathology [25,40]. Pathogenic events involving one or more branches of such pathways have been described. Even though the ideal target tissue, brain, could not be investigated, our observation of decreased tryptophan metabolism in cells from patients with ASDs may provide a unifying model that could help explain the genetic heterogeneity of ASDs. Our findings perhaps represent a preliminary step in the development of an array which could be used to provide a quick and reliable screening test for ASDs.

## Abbreviations

ADHD: Attention deficit/hyperactivity disorder; ADI-R: Autism diagnostic interview-revised; ADOS: Autism diagnostic observation schedule; ASDs: Autism spectrum disorders; CNS: Central nervous system; DSM: Diagnostic and statistical manual of mental disorders; FDR: False discovery rate; IRB: Institutional review board; NAD+: Nicotinamide adenine dinucleotide; NADH: Nicotinamide adenine dinucleotide reduced form; NADPH: Nicotinamide adenine dinucleotide phosphate reduced form; NO: Nitric oxide; PM: Phenotype MicroArray; RNA: Ribonucleic acid; SAM: Significance analysis of microarray; SCAP: South Carolina Autism Project.

## Competing interests

Barry Bochner is President, CEO, and CSO of Biolog. The other authors declare that they have no competing interests.

## Authors’ contributions

LB, AP, and HM conducted experiments, LB, CFC, BRB, and CES contributed to writing of the paper, LB designed experiments, CFC conducted statistical analyses and data mining, CS and RES assisted with collection of patient material, BRB provided advice on the Biolog platform, CES designed the overall project. All authors read and approved the final manuscript.

## Supplementary Material

Additional file 1: Table S1 Genotype and phenotype of the first 17 patients with ASDs tested. Notes. del, deletion; dup, duplication; ID, intellectual disability; ukn, unknown. Click here for file

Additional file 2: Table S2 Absorbance data of PM-M1 to M4 plates for the first 18 controls (C1-C18) and 17 patients with ASDs (A1-A17). Notes. The data were log-transformed before undergoing statistical analyses. The wells containing tryptophan are indicated in red. The wells with *P* value ≤0.05 are in bold. Click here for file

Additional file 3: Table S3 Absorbance data of PM-M1 to M4 plates for the first 18 controls (C1-C18) and 15 patients with different conditions characterized by ID (N1-N15). Notes. The data were log-transformed before undergoing statistical analyses. The wells containing tryptophan are indicated in red. The wells with *P *value <0.05 are in bold. Click here for file

Additional file 4: Table S4 Significant absorbance data of PM-M4 wells for 20 controls (C1-C20) and 20 patients with ASDs (A1-A20). Notes. The data were log-transformed before undergoing statistical analyses. The wells containing tryptophan are indicated in red. Click here for file

Additional file 5: Table S5 Expression microarray data for genes involved in tryptophan metabolic pathways in 10 patients with ASDs versus 10 controls. Notes. Each cell line from the 10 patients with ASDs was individually compared to the control group using the Mann–Whitney one sample test. Bold digits denote that control intensities are well above detection threshold, red color denotes that the gene is expressed in the patient significantly more than in controls, blue color denotes that the gene is expressed in the patient significantly less than in controls. Click here for file

## References

[B1] Autism and Developmental Disabilities Monitoring Network Surveillance Year 2006 Principal Investigators; Centers for Disease Control and Prevention (CDC) Prevalence of autism spectrum disorders - autism and developmental disabilities monitoring network, United States, 2006 MMWR Surveill Summ 2009 58 1 20 20023608

[B2] Ganz ML The lifetime distribution of the incremental societal costs of autism Arch Pediatr Adolesc Med 2007 161 343 349 17404130 10.1001/archpedi.161.4.343

[B3] Valicenti-McDermott M Hottinger K Seijo R Shulman L Age at diagnosis of autism spectrum disorders J Pediatr 2012 161 554 556 22683037 10.1016/j.jpeds.2012.05.012

[B4] Schaefer GB Mendelsohn NJ Clinical genetics evaluation in identifying the etiology of autism spectrum disorders Genet Med 2008 10 301 305 18414214 10.1097/GIM.0b013e31816b5cc9 PMC3111012

[B5] Miles JH Autism spectrum disorders–a genetics review Genet Med 2011 13 278 294 21358411 10.1097/GIM.0b013e3181ff67ba

[B6] Anderson GM Lombroso PJ Genetics of childhood disorders: XLV. Autism, part 4: serotonin in autism J Am Acad Child Adolesc Psychiatry 2002 41 1513 1516 12447040 10.1097/00004583-200212000-00025

[B7] Yap IK Angley M Veselkov KA Holmes E Lindon JC Nicholson JK Urinary metabolic phenotyping differentiates children with autism from their unaffected siblings and age-matched controls J Proteome Res 2010 9 2996 3004 20337404 10.1021/pr901188e

[B8] Adams JB Audhya T McDonough-Means S Rubin RA Quig D Geis E Gehn E Loresto M Mitchell J Atwood S Barnhouse S Lee W Nutritional and metabolic status of children with autism vs. neurotypical children, and the association with autism severity Nutr Metab (Lond) 2011 8 34 21651783 10.1186/1743-7075-8-34 PMC3135510

[B9] Schroer RJ Phelan MC Michaelis RC Crawford EC Skinner SA Cuccaro M Simensen RJ Bishop J Skinner C Fender D Stevenson RE Autism and maternally derived aberrations of chromosome 15q Am J Med Genet 1998 76 327 336 9545097 10.1002/(sici)1096-8628(19980401)76:4<327::aid-ajmg8>3.0.co;2-m

[B10] Bochner BR Siri M Huang RH Noble S Lei XH Clemons PA Wagner BK Assay of the multiple energy-producing pathways of mammalian cells PLoS One 2011 6 e18147 21455318 10.1371/journal.pone.0018147 PMC3063803

[B11] Putluri N Shojaie A Vasu VT Nalluri S Vareed SK Putluri V Vivekanandan-Giri A Byun J Pennathur S Sana TR Fischer SM Palapattu GS Creighton CJ Michailidis G Sreekumar A Metabolomic profiling reveals a role for androgen in activating amino acid metabolism and methylation in prostate cancer cells PLoS One 2011 6 e21417 21789170 10.1371/journal.pone.0021417 PMC3138744

[B12] Hughes TR Mao M Jones AR Burchard J Marton MJ Shannon KW Lefkowitz SM Ziman M Schelter JM Meyer MR Kobayashi S Davis C Dai H He YD Stephaniants SB Cavet G Walker WL West A Coffey E Shoemaker DD Stoughton R Blanchard AP Friend SH Linsley PS Expression profiling using microarrays fabricated by an ink-jet oligonucleotide synthesizer Nat Biotechnol 2001 19 342 347 11283592 10.1038/86730

[B13] Churchill GA Fundamentals of experimental design for cDNA microarrays Nat Genet 2002 Suppl 490 495 12454643 10.1038/ng1031

[B14] Tusher VG Tibshirani R Chu G Significance analysis of microarrays applied to the ionizing radiation response Proc Natl Acad Sci USA 2001 98 5116 5121 11309499 10.1073/pnas.091062498 PMC33173

[B15] Storey JD Tibshirani R Statistical significance for genomewide studies Proc Natl Acad Sci USA 2003 100 9440 9445 12883005 10.1073/pnas.1530509100 PMC170937

[B16] Witten IH Eibe F Data Mining: Practical Machine Learning Tools and Techniques 2005 San Francisco, CA: Morgan Kaufman

[B17] Chen CF Cheng CH Regulation of cellular metabolism and cytokines by the medicinal herb feverfew in the human monocytic THP-1 cells Evid Based Complement Alternat Med 2009 6 91 98 18955216 10.1093/ecam/nem061 PMC2644270

[B18] Stone TW Darlington LG Endogenous kynurenines as targets for drug discovery and development Nat Rev Drug Discov 2002 1 609 620 12402501 10.1038/nrd870

[B19] Weiss LA Shen Y Korn JM Arking DE Miller DT Fossdal R Saemundsen E Stefansson H Ferreira MA Green T Platt OS Ruderfer DM Walsh CA Altshuler D Chakravarti A Tanzi RE Stefansson K Santangelo SL Gusella JF Sklar P Wu BL Daly MJ Autism Consortium Association between microdeletion and microduplication at 16p11.2 and autism N Engl J Med 2008 358 667 675 18184952 10.1056/NEJMoa075974

[B20] Rossignol DA Frye RE Mitochondrial dysfunction in autism spectrum disorders: a systematic review and meta-analysis Mol Psychiatry 2012 17 290 314 21263444 10.1038/mp.2010.136 PMC3285768

[B21] Mattson MP Liu D Energetics and oxidative stress in synaptic plasticity and neurodegenerative disorders Neuromolecular Med 2002 2 215 231 12428812 10.1385/NMM:2:2:215

[B22] Anderson MP Hooker BS Herbert MR Bridging from cells to cognition in autism pathophysiology: biological pathways to defective brain function and plasticity Am J Biochem Biotechnol 2008 4 167 176

[B23] Bourgeron T A synaptic trek to autism Curr Opin Neurobiol 2009 19 231 234 19545994 10.1016/j.conb.2009.06.003

[B24] Kelleher RJ 3rd Bear MF The autistic neuron: troubled translation? Cell 2008 135 401 406 18984149 10.1016/j.cell.2008.10.017

[B25] Casanova MF The neuropathology of autism Brain Pathol 2007 17 422 433 17919128 10.1111/j.1750-3639.2007.00100.x PMC8095561

[B26] Courchesne E Mouton PR Calhoun ME Semendeferi K Ahrens-Barbeau C Hallet MJ Barnes CC Pierce K Neuron number and size in prefrontal cortex of children with autism JAMA 2011 306 2001 2010 22068992 10.1001/jama.2011.1638

[B27] Sweeten TL Posey DJ Shankar S McDougle CJ High nitric oxide production in autistic disorder: a possible role for interferon-gamma Biol Psychiatry 2004 55 434 437 14960298 10.1016/j.biopsych.2003.09.001

[B28] Sogut S Zoroglu SS Ozyurt H Yilmaz HR Ozugurlu F Sivasli E Yetkin O Yanik M Tutkun H Savas HA Tarakcioglu M Aykol O Changes in nitric oxide levels and antioxidant enzyme activities may have a role in the pathophysiological mechanisms involved in autism Clin Chim Acta 2003 331 111 117 12691871 10.1016/s0009-8981(03)00119-0

[B29] Barnes NM Sharp T A review of central 5-HT receptors and their function Neuropharmacology 1999 38 1083 1152 10462127 10.1016/s0028-3908(99)00010-6

[B30] Chugani DC Muzik O Behen M Rothermel R Janisse JJ Lee J Chugani HT Developmental changes in brain serotonin synthesis capacity in autistic and nonautistic children Ann Neurol 1999 45 287 295 10072042 10.1002/1531-8249(199903)45:3<287::aid-ana3>3.0.co;2-9

[B31] Schaechter JD Wurtman RJ Serotonin release varies with brain tryptophan levels Brain Res 1990 532 203 210 1704290 10.1016/0006-8993(90)91761-5

[B32] Moskowitz DS Pinard G Zuroff DC Annable L Young SN The effect of tryptophan on social interaction in everyday life: a placebo-controlled study Neuropsychopharmacology 2001 25 277 289 11425511 10.1016/S0893-133X(01)00219-6

[B33] Johansson J Landgren M Fernell E Vumma R Ahlin A Bjerkenstedt L Venizelos N Altered tryptophan and alanine transport in fibroblasts from boys with attention-deficit/hyperactivity disorder (ADHD): an in vitro study Behav Brain Funct 2011 7 40 21942982 10.1186/1744-9081-7-40 PMC3191351

[B34] Sugden K Tichopad A Khan N Craig IW D’Souza UM Genes within the serotonergic system are differentially expressed in human brain BMC Neurosci 2009 10 50 19445671 10.1186/1471-2202-10-50 PMC2697991

[B35] Zill P Buttner A Eisenmenger W Moller HJ Ackenheil M Bondy B Analysis of tryptophan hydroxylase I and II mRNA expression in the human brain: a post-mortem study J Psychiatr Res 2007 41 168 173 16023677 10.1016/j.jpsychires.2005.05.004

[B36] Daubert EA Condron BG Serotonin: a regulator of neuronal morphology and circuitry Trends Neurosci 2010 33 424 434 20561690 10.1016/j.tins.2010.05.005 PMC2929308

[B37] Bonnin A Goeden N Chen K Wilson ML King J Shih JC Blakely RD Deneris ES Levitt P A transient placental source of serotonin for the fetal forebrain Nature 2011 472 347 350 21512572 10.1038/nature09972 PMC3084180

[B38] Voineagu I Wang X Johnston P Lowe JK Tian Y Horvath S Mill J Cantor RM Blencowe BJ Geschwind DH Transcriptomic analysis of autistic brain reveals convergent molecular pathology Nature 2011 474 380 384 21614001 10.1038/nature10110 PMC3607626

[B39] Casanova MF van Kooten IA Switala AE van Engeland H Heinsen H Steinbusch HW Hof PR Trippe J Stone J Schmitz C Minicolumnar abnormalities in autism Acta Neuropathol 2006 112 287 303 16819561 10.1007/s00401-006-0085-5

[B40] Minshew NJ Williams DL The new neurobiology of autism: cortex, connectivity, and neuronal organization Arch Neurol 2007 64 945 950 17620483 10.1001/archneur.64.7.945 PMC2597785

