# Supplementary material for: Decreased tryptophan metabolism in patients with autism spectrum disorders
Source: Mol Autism. 2013 Jun 3;4:16. doi: 10.1186/2040-2392-4-16 (PMC3680090; doi:10.1186/2040-2392-4-16)
Supplement: Additional file 1: Table S1 — Genotype and phenotype of the first 17 patients with ASDs tested. Notes. del, deletion; dup, duplication; ID, intellectual disability; ukn, unknown. [file 2040-2392-4-16-S1.pdf]

Additional Table 1. Genotype and phenotype of the first 17 patients with ASDs tested.

Patient	Genetics	Comments	ID	Seizures	Speech delay	Motor impairment	Facial dysmorphisms	Aggressive/ repetitive behavior	Other features
1	*NLGN4* p.Ala289Thr	The change is considered to be pathogenic.	no	no	yes	no	slight eversion of lower lip, attached earlobes	yes (hyperactivity)	overgrowth, recurrent ear infections
2	*NLGN4* p.Ser259Pro	The change is considered to be pathogenic.	yes	no	yes (regression)	yes ("clumsy" gross motor skills)	depressed nasal bridge, broad nasal tip, full lips, attached earlobes	yes (outbursts)	recurrent ear infections, abdomen striae
3	(*SHANK3* c.1304+48C>T) + (chr. 18p11.31 dup)	The *SHANK3* change (SNP rs76224556) is supposed to affect the gene expression. Chr. 18p11.31 dup (position 6.1-6.5Mb, genome build hg 17). One gene involved (*L3MBTL4*). Patient's mother was normal by OGT array. The clinical significance of this variant is unclear.	yes	no	yes (regression)	yes (regression)	slight bitemporal depression, slightly upslanting palpebral fissures, epicanthal folds, a thin upper lip with a bow shape, full cheeks	yes (aggressive/ repetitive behavior)	overgrowth
4	Chr. 2p21 dup + chr. 20p21.1 del	Chr. 2p21 dup (approximately 563 kb, 45,265,479-45,828,359, genomic build hg 18). Two genes involved, one has an OMIM entry (*PRKCE*). Chr. 20p21.1 del (approximately 218 kb, 14,823,878-15,041,954, genomic build hg 18). One gene involved (*MACROD2*). No parental studies were performed. The clinical significance of these copy number variants is unclear.	yes	no	yes	yes	prominent forehead and occiput, recessed eyes, mild epicanthal folds, traingular face, upslanted palpebral fissures, ptosis, full lips, thin hair, high arched palate, missing teeth and poor enamel,	no	neonatal jaundice, macrocephaly, pectus excavatum, radial-ulnar synostosis, hydrocephalus, small penis
5	Chr. 3p26.2 del	Chr. 3p26.2 deletion (159 kb, 4,067,922-4,227,267, genomic build hg 18). No genes involved. No parental studies were performed. The clinical significance of this copy loss is unclear.	yes	no	yes	yes (decreased fine and gross motor skills)	slightly flat midface	no	sensitivity issues, neonatal jaundice
6	Chr. 3p26.2 del	Chr. 3p26.2 deletion (159 kb, 4,067,922-4,227,267, genomic build hg 18). No genes involved. No parental studies were performed. The clinical significance of this copy loss is unclear.	yes	no	yes	no	slightly flat midface	no	sensitivity issues, neonatal jaundice
7	No significant genetic finding	Array-CGH and methylation arrays did not identified significant abnormalities.	yes	no	yes (regression)	no	flat midface, full lips	no	no
8	(*SHANK3* c.1304+48C>T) + (*EGR2* SNPs)	The *SHANK3* change (SNP rs76224556) is supposed to affect the gene expression.	no	yes	yes	yes	bilateral epicanthal folds, slightly upturned nose, thickened helices and fleshy lobes at the ears	no	overgrowth
9	*SHANK3* c.1304+48C>T	The *SHANK3* change (SNP rs76224556) is supposed to affect the gene expression.	yes	yes	yes	yes (left spastic hemiparesis)	full lips, a short philtrum, upslanting palpebral fissures, recessed-appearing eyes, unattached earlobes	yes (mannerism)	recurrent ear infections, sinus problems
10	t 14;15, ish der(14)t(14;15)(q10?;p10?)(D15Z1+),15q11q13(D15S10x2)	The translocation occurred *de novo.*	yes	no	yes (regression)	no	no	yes (aggressive/ hyperactive behavior)	positive family history for ID and speech delay (both sides), recurrent skin infections, EEG abnormalities
11	*FMR1* full methylation	The change is considered to be pathogenic.	yes	ukn	ukn	ukn	ukn	ukn	ukn
12	*FMR1* full methylation	The change is considered to be pathogenic.	yes	ukn	ukn	ukn	ukn	ukn	ukn
13	*FMR1* full methylation	The change is considered to be pathogenic.	yes	no	yes	yes	no	no	kyphosis
14	*FMR1* full methylation	The change is considered to be pathogenic.	yes	no	no	no	no	no	no
15	*MECP2* p.Arg168X	The change is considered to be pathogenic.	yes	yes	yes (regression)	yes (stereotypical hand movements)	ukn	yes	ukn
16	*MECP2* p.Met158Thr	The change is considered to be pathogenic.	yes	yes	yes (regression)	yes (regression)	no	no	microcephaly
17	*ZNF711* c.2157_2158 del TG; p.719fs*1	The change is considered to be pathogenic.	yes	no	yes	no	no	yes	no
