# Supplementary material for: Decreased tryptophan metabolism in patients with autism spectrum disorders
Source: Mol Autism. 2013 Jun 3;4:16. doi: 10.1186/2040-2392-4-16 (PMC3680090; doi:10.1186/2040-2392-4-16)
Supplement: Additional file 2: Table S2 — Absorbance data of PM-M1 to M4 plates for the first 18 controls (C1-C18) and 17 patients with ASDs (A1-A17). Notes. The data were log-transformed before undergoing statistical analyses. The wells containing tryptophan are indicated in red. The wells with P value ≤0.05 are in bold. [file 2040-2392-4-16-S2.pdf]

Additional Table 2. Absorbance data of PM-M1 to M4 plates for the first 18 controls (C1-C18) and 17 patients with ASDs (A1-A17).

Substrate	C1	C2	C3	C4	C5	C6	C7	C8	C9	C10	C11	C12	C13	C14	C15	C16	C17	C18	A1	A2	A3	A4	A5	A6	A7	A8	A9	A10	A11	A12	A13	A14	A15	A16	A17	*P* value
Trp-Trp	-0.94	-1.56	-0.97	-1.12	-1.69	-0.94	-0.95	-1.24	-0.81	-0.92	-0.92	-1.66	-0.27	-0.53	-0.90	-0.90	-0.56	-1.07	-2.36	-1.75	-1.73	-2.00	-1.57	-1.76	-1.51	-1.21	-1.29	-1.68	-1.96	-1.58	-1.52	-1.55	-2.33	-2.07	-1.55	0.00000047
Tryptophan	-0.99	-1.23	-0.61	-1.13	-1.53	-0.89	-0.86	-1.12	-0.29	-0.83	-0.69	-0.98	0.33	0.18	-0.66	-0.71	-0.28	-0.94	-2.22	-1.40	-1.71	-1.72	-1.41	-1.77	-1.18	-1.54	-1.34	-1.16	-1.77	-1.46	-1.32	-1.18	-2.19	-1.93	-1.41	0.00000100
Trp-Gly	-1.33	-1.63	-1.21	-0.98	-1.94	-1.16	-1.22	-1.41	-0.68	-1.12	-1.12	-1.51	-0.29	-0.45	-1.00	-0.89	-0.68	-1.21	-2.85	-1.84	-2.24	-2.17	-2.08	-1.95	-1.91	-1.36	-1.46	-1.57	-2.13	-1.64	-1.48	-1.48	-2.63	-2.20	-1.90	0.00000114
Ala-Trp	-1.68	-1.52	-1.05	-1.08	-1.86	-1.40	-1.33	-1.37	-0.72	-1.40	-1.03	-1.46	-0.15	-0.31	-1.00	-0.86	-0.58	-1.28	-2.48	-1.89	-2.19	-2.06	-1.69	-1.62	-1.62	-1.65	-1.80	-1.59	-1.85	-1.70	-1.89	-1.57	-2.62	-2.04	-1.74	0.00000224
Trp-Arg	-1.54	-1.63	-1.28	-1.16	-2.22	-1.34	-1.33	-1.83	-1.04	-1.32	-1.32	-1.92	-0.65	-0.69	-1.31	-1.22	-0.92	-1.59	-3.02	-2.16	-2.45	-2.41	-2.08	-1.76	-2.01	-1.86	-2.07	-1.85	-2.34	-1.75	-1.66	-1.52	-3.04	-2.37	-2.12	0.00000289
Asp-Trp	-1.57	-1.54	-1.14	-1.50	-1.93	-1.51	-1.28	-1.64	-0.68	-1.22	-1.17	-1.53	-0.25	-0.40	-1.15	-1.11	-0.74	-1.47	-2.86	-1.97	-2.33	-2.19	-2.00	-1.79	-1.90	-1.89	-2.18	-1.31	-2.29	-1.47	-1.71	-1.37	-2.73	-2.47	-2.05	0.00000456
Met-Trp	-1.58	-1.22	-1.09	-1.27	-0.63	-1.05	-1.07	-1.50	-0.65	-1.06	-1.06	-1.40	-0.09	-0.20	-0.91	-0.66	-0.36	-0.92	-2.47	-1.73	-1.63	-1.58	-1.26	-1.73	-1.43	-1.62	-1.38	-1.20	-1.69	-1.44	-1.40	-1.39	-2.27	-1.80	-1.52	0.00000652
Glu-Trp	-1.19	-1.40	-1.08	-1.12	-1.55	-1.16	-1.04	-1.74	-0.40	-0.92	-0.95	-1.07	-0.03	-0.22	-1.04	-1.06	-0.36	-1.10	-2.76	-1.81	-2.02	-1.76	-1.52	-1.73	-1.48	-1.64	-1.97	-0.86	-2.13	-1.74	-1.43	-1.08	-2.62	-2.38	-1.60	0.00001172
Trp-Tyr	-0.45	-1.45	-1.01	-1.42	-2.01	-1.27	-1.37	-1.46	-0.83	-1.28	-1.28	-1.75	-0.24	-0.54	-0.95	-1.12	-0.68	-1.43	-2.92	-1.72	-2.24	-1.93	-1.57	-1.98	-1.69	-1.29	-1.55	-1.65	-2.21	-1.77	-1.68	-1.49	-2.67	-2.32	-1.80	0.00001418
Trp-Glu	-1.29	-1.40	-1.01	-1.17	-1.93	-1.13	-1.14	-1.39	-0.53	-0.97	-0.97	-1.43	-0.31	-0.45	-1.18	-1.30	-0.89	-1.77	-2.91	-1.85	-2.30	-2.15	-1.88	-1.63	-1.66	-1.19	-1.99	-1.79	-2.11	-1.50	-1.56	-1.06	-2.82	-2.47	-1.97	0.00001459
Trp-Ala	-1.23	-1.60	-1.15	-1.01	-1.99	-1.04	-1.09	-1.40	-0.76	-1.07	-1.07	-1.59	-0.22	-0.36	-1.04	-0.87	-0.67	-1.34	-2.66	-1.87	-2.17	-2.09	-1.60	-1.67	-1.54	-1.19	-1.53	-1.27	-1.94	-1.54	-1.61	-1.51	-2.48	-1.91	-1.69	0.00001853
Trp-Ser	0.10	-1.61	-1.34	-1.06	-2.02	-1.21	-1.25	-1.45	-0.82	-1.20	-1.20	-1.93	-0.25	-0.43	-0.85	-0.96	-0.71	-1.35	-2.59	-1.66	-2.11	-1.97	-1.75	-2.15	-1.63	-1.43	-1.33	-1.69	-1.86	-1.73	-1.77	-1.65	-2.24	-1.95	-1.78	0.00002311
Arg-Trp	-1.82	-1.56	-1.25	-1.67	-2.40	-1.68	-1.60	-1.78	-1.01	-1.78	-1.63	-2.07	-0.83	-0.72	-1.54	-1.30	-1.16	-1.83	-2.98	-2.47	-2.84	-2.56	-2.20	-1.55	-2.29	-2.01	-1.54	-1.89	-2.55	-2.43	-1.73	-1.77	-2.91	-2.34	-2.11	0.00003795
Trp-Phe	-1.00	-1.38	-1.11	-1.10	-1.38	-1.16	-1.16	-1.68	-1.03	-1.63	-1.63	-1.96	-0.80	-0.97	-1.12	-0.94	-0.86	-1.23	-2.47	-1.49	-1.77	-1.70	-1.26	-1.98	-1.74	-1.27	-1.44	-1.77	-1.87	-1.68	-1.74	-1.47	-2.48	-1.82	-1.76	0.00005340
Trp-Leu	-1.47	-1.49	-1.22	-1.22	-1.89	-0.91	-1.29	-1.66	-0.72	-1.24	-1.24	-1.65	-0.44	-0.63	-1.40	-0.80	-0.57	-1.18	-2.36	-1.60	-2.14	-1.82	-1.38	-2.05	-1.75	-1.47	-1.11	-1.58	-1.42	-1.64	-2.18	-1.64	-2.64	-1.92	-1.67	0.00006259
Leu-Trp	-1.08	-1.54	-1.02	-1.14	-1.89	-0.99	-1.02	-1.32	-0.57	-1.15	-1.15	-1.44	-0.28	-0.37	-1.02	-0.62	-0.51	-0.66	-2.55	-1.62	-2.29	-1.73	-1.26	-1.90	-1.63	-1.56	-1.08	-1.48	-1.19	-1.50	-1.55	-1.52	-1.92	-1.06	-1.64	0.00006689
Trp-Asp	-1.34	-1.76	-1.36	-1.29	-2.20	-1.33	-1.30	-1.73	-0.93	-1.26	-1.26	-1.90	-0.53	-0.67	-1.13	-1.09	-0.76	-1.76	-3.01	-1.96	-2.55	-2.34	-1.87	-1.53	-2.03	-1.34	-1.95	-1.84	-2.29	-1.66	-1.49	-1.34	-2.93	-2.73	-2.16	0.00007259
Phe-Trp	-1.27	-1.41	-1.31	-1.37	-1.55	-1.05	-1.33	-1.82	-1.09	-1.53	-1.53	-1.89	-0.98	-0.99	-1.49	0.32	-0.76	-1.31	-2.40	-2.13	-1.93	-2.06	-1.75	-1.94	-1.87	-1.32	-1.56	-1.64	-1.97	-1.51	-1.38	-1.49	-2.49	-2.14	-1.80	0.00016845
Trp-Val	-0.78	-1.57	-1.30	-2.00	-2.15	-1.13	-1.22	-1.43	-0.57	-0.99	-0.99	-1.56	-0.18	-0.37	-1.27	-1.04	-0.57	-1.49	-2.65	-1.69	-2.43	-1.87	-1.56	-2.01	-1.57	-1.28	-1.81	-1.52	-1.95	-1.50	-1.54	-1.26	-2.50	-2.23	-1.69	0.00016939
Tyr-Trp	-1.55	-1.73	-1.48	-1.55	-2.06	-1.65	-1.63	-1.97	-1.16	-1.68	-1.68	-2.24	-1.00	-1.16	-1.25	-1.20	-0.83	-1.42	-3.04	-2.51	-2.64	-2.33	-2.04	-2.37	-2.37	-1.47	-1.11	-1.82	-1.72	-2.07	-1.94	-1.69	-2.98	-2.42	-2.12	0.00020691
Gly-Trp	-1.79	-1.59	-1.10	-1.11	-3.05	-1.21	-1.18	-1.51	-0.74	-1.19	-1.19	-1.40	-0.13	-0.31	-1.02	-0.89	-0.46	-1.16	-2.86	-1.75	-1.98	-1.66	-1.52	-1.46	-1.49	-1.86	-1.59	-1.69	-1.82	-1.78	-1.80	-1.63	-2.65	-2.26	-1.74	0.00058018
Ile-Trp	-1.66	-1.23	-0.83	-1.05	-2.72	-1.05	-1.01	-1.35	-0.47	-0.96	-0.96	-1.07	0.04	-0.05	-0.90	-0.72	-0.41	-1.09	-2.59	-1.69	-2.02	-1.77	-1.52	-1.66	-1.42	-1.46	-1.49	-1.08	-1.94	-1.53	-1.22	-0.86	-1.92	-1.78	-1.63	0.00072739
Tyr-Gly	-1.26	-1.89	-2.13	-1.73	-2.99	-2.11	-2.09	-2.20	-1.46	-1.91	-1.91	-2.55	-1.05	-1.18	-1.50	-1.33	-1.32	-1.91	-3.29	-2.86	-3.28	-2.90	-2.69	-2.09	-2.77	-1.70	-1.89	-1.94	-2.70	-1.98	-2.04	-1.62	-3.22	-3.88	-2.51	0.00074275
Trp-Lys	-1.86	-1.64	-1.67	-1.56	-2.40	-1.12	-1.31	-2.00	-0.96	-1.32	-1.32	-1.81	-0.41	-0.55	-1.13	-0.98	-0.71	-1.50	-2.81	-1.48	-2.10	-1.68	-1.41	-2.32	-1.39	-1.60	-1.65	-1.84	-2.32	-1.84	-1.77	-1.61	-2.82	-2.44	-1.84	0.00111199
His-Trp	-2.32	-1.30	-0.94	-1.09	-3.54	-1.53	-1.57	-2.01	-0.80	-1.58	-1.58	-1.96	-0.67	-0.83	-1.63	-0.93	-0.58	-1.28	-2.97	-2.05	-2.49	-2.15	-2.02	-1.72	-2.13	-2.17	-1.97	-1.65	-2.35	-2.06	-1.76	-1.81	-2.51	-2.34	-2.01	0.00136297
Ser-Tyr	-1.88	-1.74	-1.81	-1.47	-2.42	-2.63	-2.13	-2.26	-1.34	-1.62	-1.62	-2.14	-1.03	-1.14	-1.53	-1.29	-1.19	-1.63	-2.65	-2.64	-3.13	-2.74	-2.60	-2.32	-2.34	-1.70	-1.48	-1.85	-2.05	-2.04	-1.91	-1.68	-2.90	-2.09	-2.24	0.00149068
Gly-Tyr	-2.29	-2.01	-2.11	-1.61	-3.41	-2.34	-2.17	-2.21	-1.39	-1.76	-1.76	-2.18	-1.06	-1.13	-1.54	-1.45	-1.28	-1.87	-3.56	-2.59	-2.93	-2.54	-2.55	-1.85	-2.43	-2.10	-2.12	-2.07	-2.59	-2.12	-2.19	-1.69	-3.17	-2.47	-2.40	0.00257219
Tyr-Val	-1.69	-1.89	-2.15	-2.40	-2.84	-2.06	-2.10	-2.49	-1.26	-1.69	-1.69	-2.12	-0.81	-1.06	-1.36	-1.79	-1.38	-2.20	-3.54	-2.88	-3.21	-2.62	-2.54	-1.83	-2.31	-1.56	-2.31	-1.82	-3.18	-1.92	-1.92	-1.61	-3.49	-2.82	-2.70	0.00258897
Glycerol Phosphate	-1.17	-0.20	-0.15	1.35	-1.31	-0.75	0.17	-1.01	-0.33	-1.01	-0.92	-1.17	-0.57	-0.16	-0.34	-0.72	-0.49	-1.03	-1.29	-1.61	-0.95	-1.99	-2.08	-1.29	-2.02	-0.68	-1.43	-1.01	-1.16	-1.21	-1.02	0.80	-1.59	-1.69	-1.19	0.00267850
Pro-Trp	-1.30	-1.52	-1.28	-0.94	-1.60	-1.17	-1.03	-1.45	-0.58	-1.06	-1.06	-1.45	-0.16	-0.34	-0.93	-0.52	-0.50	-1.16	-2.20	-1.52	-2.30	-1.39	-1.30	-0.86	-1.37	-1.03	-1.64	-0.81	-2.17	-1.49	-0.98	-1.38	-2.37	-1.22	-1.57	0.00294258
Tyr-Ile	-0.45	-1.69	-1.85	-1.80	-2.80	-2.02	-2.31	-2.25	-1.05	-1.46	-1.46	-2.04	-1.00	-1.02	-1.44	-1.34	-1.08	-1.92	-2.97	-2.64	-3.29	-2.37	-2.11	-1.97	-2.11	-1.56	-2.10	-1.63	-3.02	-1.77	-1.43	-1.35	-2.56	-2.85	-2.33	0.00313330
Glycogen	0.04	0.51	0.78	0.56	0.08	0.69	1.06	0.35	0.32	0.07	0.46	-0.26	1.21	1.24	0.75	0.73	0.79	0.79	-0.38	0.39	0.36	0.38	0.39	-0.42	-0.84	0.81	0.23	0.07	0.42	0.04	-0.14	0.46	-0.51	0.16	0.49	0.00348345
Ile-Tyr	-1.98	-1.55	-1.89	-1.37	-2.77	-2.07	-1.99	-2.37	-0.96	-1.51	-1.51	-1.95	-0.93	-0.87	-1.55	-1.20	-0.99	-2.01	-3.34	-2.61	-3.32	-2.73	-2.43	-1.49	-2.42	-1.90	-2.40	-1.69	-2.34	-1.97	-1.66	-1.05	-2.29	-2.14	-2.26	0.00364710
Met-Tyr	-2.33	-1.59	-1.89	-1.65	-3.16	-2.14	-2.04	-2.05	-1.21	-1.55	-1.55	-2.00	-0.93	-0.98	-1.38	-1.16	-1.09	-1.69	-2.94	-2.34	-2.99	-2.29	-1.95	-1.99	-2.11	-2.02	-2.10	-1.86	-2.67	-1.77	-1.80	-1.48	-3.18	-2.15	-2.22	0.00391300
Tyr-Ala	-0.44	-1.97	-2.20	-2.24	-2.71	-1.95	-2.05	-2.22	-1.46	-1.78	-1.78	-2.29	-1.00	-1.12	-1.61	-1.47	-1.48	-1.93	-2.89	-2.65	-3.15	-2.77	-2.63	-1.93	-2.47	-1.56	-1.78	-2.05	-2.20	-1.96	-1.76	-1.70	-3.25	-2.27	-2.32	0.00403727
Val-Tyr	-1.53	-1.82	-2.11	-2.20	-2.56	-2.16	-2.15	-2.31	-1.27	-1.46	-1.46	-2.13	-1.00	-1.10	-1.50	-0.56	-1.17	-2.16	-3.35	-2.70	-3.24	-2.85	-2.44	-1.70	-2.36	-1.39	-1.98	-1.56	-3.00	-1.90	-1.51	-1.54	-3.17	-2.60	-2.42	0.00442918
Ala-Tyr	-2.13	-1.89	-2.28	1.75	-2.80	-2.12	-2.13	-2.34	-1.41	-2.01	-1.85	-2.10	-1.15	-1.15	-1.60	-1.29	-1.34	-1.91	-3.06	-2.87	-3.38	-2.80	-2.73	-1.71	-2.52	-2.06	-2.04	-1.81	-2.58	-2.07	-2.13	-1.72	-3.52	-2.31	-2.30	0.00543125
Tyr-Tyr	-1.19	-1.79	-1.92	-1.78	-2.61	-2.15	-1.95	-1.99	-1.30	-1.60	-1.60	-2.02	-0.76	-1.01	-1.30	-3.52	-1.27	-1.99	-3.31	-2.36	-3.40	-2.50	-2.20	-1.79	-2.30	-1.20	-2.11	-1.97	-2.95	-2.11	-2.05	-1.74	-3.31	-2.78	-2.44	0.00569310
Pro-Tyr	-2.72	-1.81	-2.11	1.75	-2.75	-1.95	-2.29	-2.31	-1.20	-1.50	-1.50	-2.12	-0.97	-1.02	-1.53	-1.47	-1.28	-2.13	-3.17	-2.85	-3.13	-2.55	-2.56	-2.45	-2.36	-1.42	-2.01	-1.91	-2.71	-2.15	-2.03	-1.40	-2.88	-3.00	-2.33	0.00603237
Tyr-Phe	-1.77	-2.24	-2.35	-2.20	-2.98	-2.31	-2.11	-2.57	-1.52	-1.74	-1.74	-2.43	-1.40	-1.43	-1.75	-2.07	-1.56	-2.06	-3.44	-2.81	-3.37	-2.74	-2.44	-2.09	-2.61	-1.60	-1.95	-2.05	-2.75	-1.93	-2.14	-1.66	-3.05	-2.84	-2.79	0.00908217
Gly-Lys	-2.45	-2.08	-2.87	-1.99	-3.16	-2.71	-2.59	-2.73	-1.58	-2.41	-2.41	-2.78	-2.29	-2.31	-2.42	-1.54	-1.61	-2.34	-3.25	-3.41	-3.63	-3.15	-3.21	-2.11	-3.46	-2.48	-2.20	-2.75	-2.95	-2.40	-2.69	-1.46	-3.44	-2.99	-2.68	0.00915288
Lys-Lys	-1.99	-1.72	-2.73	-2.13	-2.22	-2.52	-2.70	-2.83	-1.22	-1.78	-1.78	-2.59	-1.80	-1.93	-2.10	-1.51	-1.44	-2.46	-3.38	-3.43	-3.72	-3.29	-3.12	-2.04	-3.29	-2.27	-2.38	-1.87	-3.10	-2.12	-1.54	-1.17	-3.49	-2.55	-2.73	0.01013284
Tyr-His	-0.87	-1.61	-2.08	-2.10	-3.16	-2.37	-2.44	-2.91	-1.52	-1.95	-1.95	-2.66	-1.53	-1.68	-1.40	-1.23	-1.27	-2.11	-3.61	-3.06	-3.74	-3.15	-2.63	-1.91	-2.88	-1.68	-1.67	-1.93	-3.07	-2.08	-1.75	-1.69	-3.43	-2.49	-2.57	0.01066969
His-Tyr	-2.60	-1.64	-2.00	-1.73	-3.56	-2.57	-2.36	-2.74	-1.45	-1.98	-1.98	-2.50	-1.56	-1.69	-2.21	-1.55	-1.40	-2.18	-3.44	-3.16	-3.46	-2.97	-2.94	-1.80	-3.00	-1.87	-2.67	-2.07	-2.12	-2.38	-1.78	-1.99	-3.29	-2.97	-2.64	0.01076823
Methyl Succinate	0.88	0.51	0.73	1.12	0.63	1.09	1.27	0.69	1.13	0.73	0.83	0.60	1.09	1.21	0.93	1.01	1.37	0.90	0.31	0.62	1.08	0.36	0.73	-0.49	0.07	0.96	0.63	0.50	0.79	0.27	0.56	1.18	-1.80	0.76	0.86	0.01251673
Succinamic Acid	0.66	0.09	0.38	0.92	0.58	0.92	0.93	0.40	0.98	0.48	0.73	0.40	0.88	1.09	0.66	0.77	1.28	0.84	0.06	0.58	1.04	0.17	0.56	-0.66	-0.44	0.67	0.34	0.38	0.59	-0.04	0.23	1.03	-1.86	0.43	0.72	0.01307307
Lys-Gly	-2.43	-1.90	-2.60	-1.66	-3.10	-2.40	-2.56	-2.92	-1.49	-2.05	-2.05	-2.73	-2.13	-2.10	-2.06	-1.42	-1.67	-2.38	-3.76	-3.29	-3.61	-3.38	-3.31	-2.00	-3.34	-2.12	-1.97	-2.18	-3.13	-2.16	-2.09	-1.47	-3.40	-2.67	-2.69	0.01420400
MethyMannoside	0.82	-1.06	-1.62	-0.22	-2.13	-0.53	0.11	-1.44	-0.78	-1.39	-1.10	-1.91	-0.81	-0.50	-1.39	-0.69	-0.57	-1.19	-3.08	-2.54	-2.75	-2.21	-1.32	-0.93	-2.18	-0.48	-1.23	-2.01	-1.31	-1.30	-0.44	-0.68	-2.54	-0.79	-1.37	0.01422551
His-Val	-2.90	-1.86	-2.42	-1.95	-2.50	-2.56	-2.63	-3.40	-1.37	-1.77	-1.77	-2.83	-1.92	-2.23	-2.51	-1.68	-1.77	-2.66	-3.64	-3.59	-3.80	-3.31	-3.47	-1.84	-3.54	-2.33	-2.99	-1.94	-3.43	-2.06	-1.97	-1.42	-2.70	-3.27	-2.93	0.01548688
Tween 20	-3.84	-3.35	-3.47	-3.01	-3.51	-2.88	-2.64	-3.74	-3.13	-3.21	-3.02	-3.44	-3.05	-3.84	-3.47	-3.80	-3.34	-3.68	-3.82	-3.44	-3.74	-3.78	-3.72	-4.29	-4.11	-3.46	-3.99	-3.82	-3.47	-3.90	-4.06	-3.59	-2.29	-3.88	-3.56	0.01549583
Turanose	0.69	-0.36	1.24	-1.67	-2.61	-2.36	0.13	-2.17	-0.82	-1.51	-1.23	-1.72	-1.11	-0.92	-1.63	-0.89	-0.71	-1.76	-2.89	-2.86	-3.40	-1.86	-1.46	-1.68	-2.41	-0.74	-1.54	-1.06	-2.37	-1.38	-0.72	-0.79	-2.97	-2.01	-1.51	0.01810746
Butyric Acid	-0.52	-2.23	-2.47	-0.99	-2.47	-0.87	-1.09	-2.63	-1.59	-2.67	-2.06	-2.71	-1.99	-1.59	-2.02	-1.43	-1.24	-1.63	-3.15	-3.13	-3.17	-2.65	-3.41	-3.24	-2.67	-2.40	-2.15	-1.24	-1.68	-2.60	-1.66	-1.79	-1.41	-1.82	-1.99	0.01932953
His-Ala	-2.11	-2.21	-2.58	-2.01	-0.78	-2.51	-2.48	-2.85	-1.91	-2.46	-2.46	-3.06	-2.72	-2.38	-2.25	-1.45	-1.62	-1.99	-3.41	-3.27	-3.80	-3.40	-3.41	-1.63	-3.32	-2.32	-2.17	-2.02	-2.73	-2.24	-2.02	-1.97	-3.44	-2.47	-2.67	0.01972845
Glu-Tyr	-1.57	-1.45	-1.94	-1.77	-2.56	-2.33	-2.17	-2.35	-1.11	-1.46	-1.94	-1.47	-0.99	-1.17	-1.74	-1.67	-1.31	-1.91	-3.13	-2.56	-3.01	-2.43	-2.01	-1.46	-2.01	-1.87	-2.81	-0.90	-2.27	-1.69	-1.59	-1.17	-3.27	-3.02	-2.23	0.02338762
Serine	-2.54	-1.88	-2.39	-2.06	-3.21	-2.41	-2.42	-2.48	-1.47	-2.51	-2.50	-2.71	-2.01	-1.98	-2.04	-1.45	-1.44	-2.07	-3.49	-3.34	-3.63	-3.35	-2.88	-2.06	-3.20	-2.62	-2.10	-2.27	-2.07	-2.29	-2.10	-1.53	-3.13	-2.24	-2.60	0.02455322
Val-Gln	0.31	-0.84	-1.04	0.16	-2.01	-0.52	-0.68	-1.45	0.16	-0.44	-0.44	-0.61	-0.12	-0.44	-0.49	-0.76	-0.54	-0.38	-1.81	-0.39	-2.70	-3.80	-0.58	-1.74	-0.71	-0.58	-0.63	-0.72	-0.56	-0.50	-0.54	-1.38	-1.10	-0.69	-1.40	0.02791521
Dextrin	0.22	0.29	0.46	0.49	0.17	0.15	0.79	0.64	-0.04	-0.41	0.16	-0.66	0.98	1.14	0.76	0.38	0.73	0.56	-0.39	0.21	-0.09	-0.06	0.26	-0.29	-0.68	0.79	0.41	0.16	0.47	0.03	0.06	0.15	-1.99	0.03	0.28	0.02979466
Gly-Ala	-1.89	-2.18	-2.67	-2.21	-2.94	-2.68	-2.63	-3.07	-1.74	-2.40	-2.32	-2.84	-2.18	-2.29	-2.24	-1.43	-1.51	-2.08	-3.05	-3.22	-3.66	-3.00	-2.69	-2.18	-3.22	-2.51	-2.48	-2.07	-2.62	-2.22	-2.11	-1.90	-3.44	-2.56	-2.47	0.03035781
His-Asp	-2.46	-2.02	-2.12	-2.04	-2.94	-2.56	-2.61	-2.73	-1.37	-1.74	-1.74	-2.54	-1.73	-2.21	-2.09	-1.56	-1.54	-2.40	-3.57	-3.38	-3.47	-3.22	-3.24	-2.16	-2.92	-2.20	-2.34	-1.56	-2.37	-2.02	-1.91	-1.41	-2.98	-2.26	-2.82	0.03092895
Ile-Gln	-0.34	-0.30	0.20	0.68	-1.53	0.09	0.28	-0.34	0.50	-0.10	-0.10	-0.83	0.53	0.31	0.04	0.34	-0.31	0.05	-1.38	-0.32	-0.88	-0.68	-0.03	-1.23	-0.51	-0.24	-0.34	-0.26	-0.07	-0.03	0.12	-0.16	-0.34	0.02	-0.71	0.03102117
Glutamine	0.41	-0.30	-0.06	0.67	-0.78	-0.96	-0.29	0.00	0.10	-0.23	-0.26	-0.24	0.45	0.14	0.10	-0.13	0.20	-0.54	-1.56	-0.04	-1.39	-0.64	-0.36	-1.14	-0.41	0.29	-0.27	-0.56	-0.31	-0.33	-0.36	0.41	-1.84	0.13	0.04	0.04147509
Phe-Tyr	-1.91	-1.73	-2.16	-2.15	-3.29	-2.33	-2.92	-2.63	-1.45	-1.70	-1.70	-2.34	-1.40	-1.50	-2.63	0.28	-1.38	-2.03	-3.10	-2.41	-3.11	-3.00	-2.56	-1.95	-2.49	-1.65	-2.37	-1.70	-3.05	-1.85	-1.77	-1.40	-3.38	-3.32	-2.55	0.04223635
His-Gly	-2.34	-1.87	-2.34	-1.71	-2.88	-2.40	-2.63	-3.17	-1.63	-2.00	-2.00	-2.69	-2.18	-2.30	-2.16	-1.43	-1.40	-2.29	-3.40	-3.40	-3.74	-3.31	-3.17	-1.55	-3.29	-2.43	-2.15	-1.90	-2.06	-2.06	-2.10	-1.61	-3.32	-2.43	-2.75	0.04274891
Gly-Val	-2.95	-2.34	-2.66	-1.82	-2.88	-2.45	-2.69	-3.01	-1.51	-1.91	-1.91	-2.60	-2.06	-2.41	-2.26	-1.99	-1.63	-2.51	-3.49	-3.43	-3.59	-3.31	-3.37	-1.91	-3.12	-2.43	-2.35	-1.96	-2.99	-2.23	-1.60	-1.49	-3.28	-3.02	-2.92	0.04292257
Tween 40	-4.13	-3.28	-3.72	-3.10	-3.70	-2.95	-3.07	-3.74	-3.32	-3.43	-3.16	-3.80	-3.12	-3.68	-3.34	-3.90	-3.46	-3.68	-4.24	-3.68	-3.94	-3.80	-3.86	-4.72	-4.06	-3.92	-4.35	-3.70	-3.56	-4.16	-4.21	-3.56	-1.82	-3.82	-3.69	0.04375550
Ala-Gly	-2.05	-2.05	-2.51	-1.91	-2.72	-2.37	-2.19	-2.59	-1.34	-2.47	-1.76	-2.52	-2.02	-2.18	-1.86	-1.38	-1.38	-2.08	-2.94	-3.25	-3.78	-3.20	-3.07	-1.72	-2.95	-1.94	-1.92	-1.57	-3.38	-1.88	-2.20	-1.51	-2.79	-2.08	-2.25	0.04505564
Lys-Val	-3.11	-2.10	-2.87	-1.97	-2.28	-2.55	-2.67	-3.47	-1.21	-1.88	-1.88	-2.51	-1.91	-2.10	-1.89	-1.85	-1.73	-2.55	-3.78	-3.66	-3.78	-3.34	-3.21	-2.15	-3.25	-2.10	-2.47	-1.63	-3.34	-2.15	-1.61	-1.26	-3.11	-2.78	-2.90	0.04733986
Maltitol	-1.95	-1.28	0.51	-2.11	-3.13	-2.50	-1.51	-3.41	-1.33	-2.24	-1.92	-2.22	-1.13	-1.74	-2.26	-1.47	-1.33	-2.33	-3.38	-2.82	-3.41	-2.81	-2.15	-1.47	-2.34	-1.88	-2.60	-1.95	-3.27	-2.04	-1.50	-0.77	-3.61	-3.32	-1.99	0.04898343
Inosine	0.51	0.76	0.98	1.26	0.52	1.06	0.96	0.62	0.42	-0.05	0.54	-0.20	0.87	1.02	0.88	0.75	0.58	1.13	0.56	0.84	1.05	0.34	0.51	-0.79	-0.61	0.82	0.66	0.66	0.86	0.34	0.33	0.32	-1.85	0.53	0.34	0.04915403
Lys-Trp	-2.94	-1.82	-2.75	-1.93	-2.46	-2.63	-2.73	-3.07	-1.39	-2.05	-2.05	-2.71	-2.04	-1.98	-2.09	-1.48	-1.55	-2.43	-3.84	-3.51	-3.70	-3.07	-3.02	-2.53	-2.90	-2.15	-2.40	-1.44	-2.80	-2.39	-1.75	-1.64	-2.95	-2.05	-2.96	0.05030216
Gly-Ile	-2.70	-2.08	-2.59	-1.89	-3.34	-2.52	-2.42	-3.32	-1.42	-1.95	-1.95	-2.56	-2.08	-2.13	-2.27	-1.70	-1.68	-2.62	-3.25	-3.25	-3.66	-3.20	-3.24	-1.87	-2.77	-2.29	-2.51	-2.18	-2.70	-2.08	-2.50	-1.27	-3.13	-2.81	-2.87	0.05045070
Glycine	-2.44	-2.19	-2.57	-2.05	-2.94	-2.41	-2.56	-2.29	-1.69	-2.27	-2.11	-2.48	-2.10	-1.98	-1.99	-1.43	-1.47	-2.11	-3.29	-3.41	-3.66	-3.12	-3.07	-1.97	-3.13	-2.13	-2.21	-2.28	-2.61	-2.21	-2.04	-1.40	-2.26	-1.74	-2.64	0.05135768
His-Ser	-2.36	-2.05	-2.58	-1.65	-3.22	-2.64	-2.77	-3.02	-1.65	-2.01	-2.01	-2.91	-2.28	-2.21	-2.09	-1.36	-1.58	-1.83	-3.61	-3.51	-3.37	-3.29	-3.32	-2.15	-3.34	-2.63	-2.13	-1.88	-2.40	-2.30	-1.97	-1.80	-2.87	-1.67	-2.72	0.05299848
Tween 80	-4.29	-3.11	-3.25	-3.06	-3.34	-2.95	-2.85	-3.70	-3.28	-3.46	-3.15	-3.47	-2.94	-3.61	-3.37	-3.46	-3.21	-3.37	-3.94	-3.28	-3.59	-3.54	-3.59	-4.29	-3.78	-4.16	-4.08	-3.38	-3.46	-4.16	-3.61	-3.46	-2.05	-3.80	-3.39	0.05398302
Valine	-3.01	-2.10	-2.88	-2.40	-3.20	-2.51	-2.84	-3.16	-1.38	-2.10	-2.16	-2.31	-2.10	-2.15	-2.37	-1.56	-1.42	-2.58	-3.47	-3.46	-4.38	-3.20	-3.20	-2.31	-2.97	-2.89	-2.78	-1.53	-3.54	-1.83	-1.81	-1.16	-3.84	-3.35	-2.62	0.05476540
Tyr-Glu	0.35	-1.55	-2.24	-1.73	-3.12	-2.24	-2.31	-2.74	-1.10	-2.14	-2.14	-1.84	-1.22	-1.55	-2.06	-1.89	-1.60	-2.61	-3.54	-3.02	-3.46	-3.16	-2.94	-2.31	-2.95	-1.84	-2.35	-2.06	-2.91	-1.82	-1.68	-1.24	-3.94	0.08	-2.82	0.05532775
Leu-Ile	0.03	-1.83	-1.96	-0.97	-1.86	-0.49	-0.82	-1.70	-0.99	-1.35	-1.35	-2.42	-1.62	-1.58	-1.51	-0.92	-1.16	-1.10	-2.58	-3.11	-3.63	-2.27	-1.91	-1.29	-2.10	-1.71	-1.43	-1.31	-0.86	-1.57	-1.01	-1.01	-1.34	-0.90	-2.26	0.05553918
Fructose-6-P	-1.12	-0.96	-0.72	-0.47	-3.08	-1.12	0.61	-0.86	-0.56	-1.34	-0.97	-1.55	-0.04	0.02	-0.75	-0.51	-0.38	-1.20	-2.63	-2.10	-2.36	-2.50	-0.48	-0.87	-1.82	-0.48	-1.09	-0.79	-1.20	-1.19	-0.84	-0.29	-3.06	-0.55	-1.22	0.05696071
Ile-Asn	-3.16	-1.58	-2.74	-1.90	-3.20	-2.48	-2.35	-3.12	-0.85	-1.63	-1.63	-1.99	-1.87	-2.52	-2.61	-1.81	-1.50	-2.70	-3.41	-3.70	-3.66	-3.46	-3.18	-1.99	-2.99	-2.40	-2.31	-1.83	-3.15	-1.64	-2.18	-1.01	-2.68	-2.85	-3.13	0.05790653
Gly-Thr	-2.40	-1.91	-2.64	-2.22	-3.16	-2.71	-2.82	-3.15	-1.69	-2.06	-2.06	-2.69	-2.13	-2.34	-1.99	-1.58	-1.59	-2.08	-3.18	-2.95	-3.44	-2.82	-2.86	-2.05	-2.88	-2.52	-2.37	-2.10	-2.79	-2.25	-1.84	-1.72	-3.15	-2.83	-2.63	0.06069040
Alaninamide	-2.29	-2.40	-2.94	-2.40	-3.16	-2.51	-3.02	-3.00	-1.79	-2.47	-2.33	-2.89	-2.24	-2.35	-2.05	-1.57	-1.69	-2.27	-2.73	-3.15	-3.44	-3.00	-2.72	-2.48	-3.38	-2.27	-2.32	-2.37	-2.78	-2.15	-2.98	-2.03	-2.59	-2.80	-2.56	0.06415266
His-Glu	-3.02	-1.81	-2.47	-1.65	-3.70	-2.66	-2.80	-3.37	-1.20	-2.13	-2.13	-1.92	-2.40	-2.95	-2.82	-1.85	-1.70	-2.70	-3.61	-3.43	-3.80	-3.38	-3.25	-2.10	-3.11	-2.68	-2.50	-2.32	-3.52	-2.05	-1.98	-1.03	-3.44	-3.24	-3.23	0.06492404
Val-Gly	-1.76	-2.28	-2.95	-3.52	-3.44	-2.70	-2.64	-2.58	-1.58	-2.13	-2.13	-2.58	-2.30	-2.20	-1.81	-0.96	-1.80	-2.55	-3.16	-3.17	-3.35	-2.95	-2.84	-3.05	-2.77	-1.84	-2.27	-2.14	-3.28	-2.32	-2.15	-1.49	-3.22	-3.32	-2.82	0.06731545
Arg-Ser	-1.13	-2.00	-2.37	-1.85	-3.10	-2.76	-2.87	-2.47	-1.51	-2.56	-1.86	-2.80	-2.14	-2.08	-1.86	-1.36	-1.53	-2.31	-3.22	-3.57	-3.90	-3.16	-3.13	-1.54	-3.13	-2.21	-1.82	-2.11	-2.02	-2.55	-1.88	-1.65	-2.92	-2.04	-2.52	0.06974765
Gly-Phe	-2.70	-2.43	-2.99	-2.82	-2.98	-2.73	-2.63	-3.25	-2.40	-2.74	-2.74	-3.29	-2.78	-3.16	-2.58	-1.60	-2.01	-2.26	-3.35	-3.59	-3.84	-3.15	-3.21	-2.27	-3.63	-2.99	-2.13	-2.99	-3.57	-2.20	-2.48	-1.67	-3.61	-3.54	-3.10	0.07183979
Alanine	-2.24	-2.12	-2.70	-1.93	-2.98	-2.48	-2.92	-2.66	-1.28	-2.43	-1.61	-2.53	-1.83	-2.19	-1.96	-1.41	-1.40	-2.25	-2.95	-3.32	-3.97	-2.94	-2.50	-2.40	-2.97	-2.43	-2.13	-1.66	-2.48	-1.80	-1.84	-1.56	-2.81	-2.82	-2.35	0.07236729
Ile-Ser	-2.30	-1.84	-2.40	-1.34	-1.03	-2.57	-2.45	-2.92	-1.16	-1.93	-1.93	-2.34	-1.86	-1.75	-1.94	-1.58	-1.69	-2.56	-3.13	-3.22	-3.64	-3.21	-3.21	-1.64	-2.82	-2.37	-1.81	-1.40	-2.43	-2.21	-1.36	-1.12	-2.21	-2.13	-2.60	0.07478478
Val-Ile	-1.35	-1.99	-3.04	-2.26	-3.25	-2.49	-2.40	-3.59	-1.21	-1.72	-1.72	-2.24	-1.68	-1.56	-1.78	-1.06	-1.98	-2.58	-3.76	-3.41	-3.64	-3.08	-2.82	-2.16	-2.48	-1.87	-1.80	-1.27	-3.32	-1.94	-1.77	-1.24	-2.79	-3.56	-3.04	0.07534649
Val-Ala	-1.15	-2.33	-2.76	-1.90	-3.06	-2.47	-2.58	-2.88	-1.50	-1.97	-1.97	-2.65	-2.08	-2.10	-1.95	-2.12	-1.62	-1.99	-3.27	-3.41	-3.84	-3.12	-3.05	-1.88	-3.13	-1.93	-1.75	-1.85	-2.55	-2.03	-1.90	-1.64	-2.97	-2.43	-2.53	0.07546570
Phe-Pro	-2.47	-2.29	-3.13	-3.08	-3.49	-2.69	-2.94	-3.22	-1.89	-2.28	-2.28	-3.06	-2.94	-2.55	-2.81	0.21	-2.10	-2.64	-3.00	-3.46	-3.76	-3.40	-3.34	-1.94	-3.44	-2.36	-3.04	-2.65	-3.43	-2.05	-2.56	-1.65	-4.06	-3.84	-3.06	0.07672437
His-Lys	-2.51	-1.76	-2.16	-1.88	-2.63	-2.85	-2.61	-2.75	-1.47	-1.97	-1.97	-2.65	-1.77	-2.15	-2.34	-1.27	-1.16	-2.11	-3.34	-3.51	-3.76	-2.72	-2.51	-1.93	-2.97	-2.52	-2.18	-1.71	-2.90	-1.98	-1.23	-1.46	-2.41	-2.63	-2.57	0.07770285
Ser-Asp	-2.40	-1.93	-2.47	-1.59	-3.41	-2.46	-2.73	-2.65	-1.35	-2.02	-2.02	-2.54	-2.03	-2.11	-1.98	-1.47	-1.52	-1.95	-3.21	-3.41	-3.68	-3.38	-3.37	-2.34	-2.59	-1.81	-2.03	-1.56	-2.09	-2.27	-1.92	-1.29	-3.25	-2.37	-2.55	0.07913741
Ser-Val	-1.89	-2.01	-2.34	-1.65	-3.11	-2.73	-2.63	-3.00	-1.42	-2.00	-2.00	-2.48	-2.11	-2.22	-2.04	-1.57	-1.56	-2.05	-2.94	-3.28	-3.80	-3.57	-3.29	-2.24	-3.20	-2.06	-1.95	-1.23	-3.11	-1.97	-1.92	-1.38	-2.74	-2.07	-2.66	0.07954767
Gly-Asn	-1.92	-1.91	-2.47	-2.37	-3.24	-2.78	-2.75	-2.65	-1.37	-2.04	-1.99	-2.56	-1.94	-1.95	-1.70	-1.35	-1.27	-2.13	-3.02	-3.22	-3.82	-2.65	-2.84	-2.22	-2.85	-2.35	-1.92	-1.29	-2.95	-2.02	-1.73	-1.46	-3.32	-2.81	-2.27	0.07973624
Threonine	-2.60	-2.04	-2.47	-1.91	-3.20	-2.44	-2.55	-2.36	-1.24	-2.08	-1.90	-2.37	-1.91	-2.02	-1.66	-1.32	-1.21	-2.15	-3.52	-3.25	-3.64	-3.29	-2.97	-1.46	-3.01	-2.15	-2.25	-1.97	-1.61	-1.80	-2.04	-1.16	-3.70	-2.14	-2.51	0.08007210
Gly-His	-2.57	-1.93	-2.47	-2.02	-3.70	-2.63	-2.57	-3.22	-1.63	-2.33	-2.33	-2.68	-2.32	-2.31	-2.59	-1.55	-1.88	-2.37	-3.54	-3.59	-3.76	-3.31	-3.15	-1.56	-3.28	-2.08	-2.48	-2.10	-3.00	-2.41	-2.17	-1.63	-3.63	-2.67	-2.81	0.08133143
Lys-Pro	-2.86	-1.80	-2.75	-1.85	-2.98	-2.61	-2.75	-2.95	-1.36	-1.83	-1.83	-2.42	-1.91	-1.91	-2.36	-1.40	-1.37	-2.27	-3.25	-3.46	-3.80	-3.05	-2.73	-2.31	-2.48	-2.50	-1.97	-1.76	-2.85	-1.94	-1.32	-1.35	-3.86	-2.63	-2.66	0.08399005
Val-Lys	-1.42	-1.91	-2.65	-2.91	-3.18	-2.59	-2.48	-3.66	-1.32	-1.94	-1.94	-2.46	-1.82	-1.91	-2.02	-0.91	-1.77	-2.75	-3.76	-3.38	-3.76	-3.07	-2.97	-1.91	-3.05	-2.02	-2.59	-0.92	-3.07	-2.03	-1.79	-1.48	-2.87	-3.52	-3.07	0.08530150
Hexanoic Acid	-0.98	-1.61	-2.27	-1.37	-1.55	-1.29	-1.00	-1.89	-1.23	-2.26	-1.65	-2.66	-1.57	-1.62	-1.46	-0.79	-0.93	-0.88	-1.58	-0.84	-1.17	-1.44	-0.66	-1.93	-2.53	-0.24	-0.87	-0.80	-0.29	-1.42	-0.55	-1.56	-1.50	-1.07	-1.36	0.08602816
Ile-Val	-2.68	-1.92	-2.66	-2.42	-2.67	-2.47	-2.43	-3.37	-1.24	-1.99	-1.99	-2.21	-2.13	-2.26	-2.60	-1.63	-1.48	-2.67	-3.63	-3.52	-3.80	-3.29	-3.20	-1.89	-2.71	-2.21	-2.23	-1.93	-3.15	-1.89	-2.22	-0.91	-2.97	-2.68	-3.03	0.08711778
Gly-Pro	-2.51	-2.04	-2.74	-2.72	-3.29	-2.64	-2.69	-2.89	-1.33	-1.93	-1.93	-2.64	-1.92	-2.29	-2.17	-1.52	-1.47	-2.08	-3.15	-3.49	-3.90	-2.69	-2.75	-2.40	-2.93	-2.28	-1.68	-2.38	-3.18	-2.07	-1.65	-1.36	-3.76	-2.80	-2.56	0.08751112
Palatinose	0.66	-1.46	-2.27	-2.20	-2.84	-2.19	-2.74	-2.37	-1.23	-2.02	-1.85	-2.34	-1.74	-1.73	-2.43	-1.33	-1.27	-2.19	-3.56	-3.00	-3.43	-2.34	-2.04	-1.93	-2.82	-2.31	-2.10	-1.83	-2.86	-1.81	-0.89	-1.13	-3.11	-2.15	-2.10	0.08773236
Fructose	1.06	1.16	1.36	1.50	1.03	1.32	1.45	1.10	1.07	0.29	1.09	0.14	1.40	1.52	1.23	1.35	1.57	1.35	0.62	1.37	1.04	1.08	1.33	0.29	0.43	1.45	1.14	0.80	1.06	0.64	0.91	1.04	-1.66	1.09	1.28	0.08903532
Melezitose	0.58	-1.65	-2.58	1.74	-3.29	-2.41	-1.60	-2.56	-1.61	-2.37	-1.83	-2.59	-1.93	-1.94	-2.28	-1.70	-1.29	-2.15	-3.59	-3.21	-3.37	-2.42	-2.22	-1.39	-2.61	-1.85	-2.49	-2.15	-2.56	-1.81	-1.39	-0.98	-3.08	-2.25	-2.17	0.08916243
Cyclodextrin	-1.83	-1.99	-2.97	-2.28	-3.38	-2.73	-1.55	-3.25	-1.60	-2.22	-2.20	-2.81	-1.51	-1.98	-2.23	-1.75	-1.63	-2.63	-3.49	-3.10	-3.40	-3.00	-2.66	-1.47	-2.74	-2.66	-2.93	-1.62	-3.05	-2.40	-2.63	-1.21	-2.90	-2.72	-2.46	0.09121427
Gly-Gly	-2.79	-2.11	-3.11	-2.05	-3.51	-2.56	-2.79	-2.80	-1.43	-2.27	-2.27	-2.63	-2.19	-2.18	-2.47	-1.57	-1.70	-2.43	-3.27	-3.37	-3.63	-3.08	-3.07	-1.51	-3.38	-2.56	-2.29	-2.24	-2.94	-2.51	-2.01	-1.55	-3.51	-2.69	-2.73	0.09561772
Asn-Val	-1.76	-1.72	-2.62	-2.68	-3.20	-2.54	-2.63	-2.39	-1.29	-1.99	-1.62	-2.29	-1.84	-2.04	-2.04	-1.43	-1.23	-2.48	-3.04	-2.05	-4.06	-2.95	-3.21	-1.95	-2.88	-2.12	-2.33	-1.78	-2.52	-2.06	-1.60	-0.99	-2.95	-3.21	-2.38	0.09867663
Proline	-1.99	-2.03	-2.52	-2.02	-3.24	-2.65	-2.88	-2.43	-1.49	-2.11	-1.89	-2.65	-1.94	-1.99	-1.84	-1.27	-1.15	-1.96	-2.98	-3.35	-3.64	-3.18	-3.17	-1.92	-2.88	-2.14	-1.91	-2.08	-2.14	-1.83	-1.61	-1.43	-3.17	-2.13	-2.40	0.09896933
Val-Asn	-1.10	-2.15	-3.05	-2.47	-3.57	-2.51	-2.66	-3.56	-1.01	-1.46	-1.46	-2.13	-1.60	-1.82	-2.02	-2.48	-1.51	-2.63	-3.61	-3.82	-4.04	-3.35	-3.29	-2.14	-3.28	-1.89	-2.01	-1.80	-3.25	-1.70	-1.56	-1.14	-2.20	-3.01	-3.02	0.09949936
Gln-Gln	0.09	-0.44	-0.38	0.43	-0.63	-1.03	-0.11	-0.83	0.29	-0.35	-0.02	-0.31	0.28	0.35	-0.26	-0.03	0.18	-0.30	-1.44	-0.06	-0.99	-0.85	-0.24	-1.13	-0.44	-0.17	-0.58	-0.17	-0.29	-0.27	-0.47	0.37	-0.82	0.06	0.21	0.10196729
Pro-Val	-3.04	-1.87	-2.93	-2.10	-3.38	-2.50	-2.65	-3.54	-1.12	-1.86	-1.86	-2.32	-2.16	-2.32	-2.23	-2.11	-1.84	-2.70	-3.88	-3.37	-3.74	-3.24	-3.20	-2.48	-3.27	-1.78	-2.75	-1.80	-3.59	-1.91	-1.87	-1.21	-3.44	-2.50	-3.08	0.10232523
Pro-Gly	-2.56	-2.11	-2.54	1.73	-3.88	-2.73	-2.56	-3.15	-1.38	-2.00	-2.00	-2.57	-1.92	-1.83	-2.13	-1.66	-1.67	-2.34	-3.56	-3.43	-3.64	-3.22	-3.02	-1.79	-2.99	-2.02	-2.30	-2.05	-2.51	-2.10	-1.43	-1.30	-3.18	-2.80	-2.88	0.10645293
Ser-Ala	-2.09	-2.24	-2.47	1.78	-2.68	-2.42	-2.60	-2.64	-1.53	-2.05	-2.05	-2.57	-2.13	-2.16	-1.88	-1.38	-1.45	-1.68	-2.98	-3.16	-3.64	-3.17	-3.20	-1.88	-3.17	-1.97	-1.71	-1.40	-2.09	-1.91	-1.86	-1.56	-3.11	-1.60	-2.24	0.10985522
Tyr-Leu	-1.28	-1.60	-1.78	-1.38	-1.86	-1.51	-1.60	-1.62	-1.39	-1.61	-1.61	-2.15	-0.76	-1.02	-1.30	-0.96	-1.14	-0.86	-2.51	-1.97	-2.81	-1.75	-1.08	-1.56	-1.79	-0.85	-1.16	-1.65	-0.82	-1.73	-1.61	-1.69	-2.38	-1.13	-2.02	0.11289113
Lys-Asp	-2.32	-1.72	-2.73	-1.88	-3.47	-2.51	-2.52	-2.88	-1.23	-1.72	-1.72	-2.53	-1.84	-1.84	-2.31	-1.53	-1.53	-2.34	-3.70	-3.22	-3.41	-3.08	-2.84	-2.15	-3.17	-2.21	-2.03	-1.64	-2.86	-2.14	-1.34	-1.32	-2.99	-1.80	-2.73	0.11532054
Ala-Gln	0.37	-0.30	-0.30	0.41	-1.03	-0.96	-0.57	-0.69	0.16	-0.78	-0.43	-0.79	0.21	-0.11	-0.46	-0.18	-0.11	-0.69	-2.01	-0.41	-1.18	-1.24	-0.70	-1.15	-0.64	0.03	-0.40	-0.62	-0.48	-0.40	-0.69	0.18	-0.78	-0.11	0.03	0.11549434
Gly-Ser	-2.43	-1.87	-2.63	-2.41	-3.32	-2.74	-2.84	-2.58	-1.68	-2.29	-2.29	-2.60	-2.21	-2.40	-2.29	-1.33	-1.28	-1.80	-2.93	-3.18	-3.74	-2.51	-2.33	-2.60	-3.02	-2.62	-1.97	-2.07	-2.87	-2.20	-2.00	-1.51	-3.63	-2.43	-2.31	0.11565616
Phe-Met	-2.50	-1.98	-2.93	-2.67	-3.43	-2.89	-2.78	-3.92	-2.24	-2.73	-2.73	-3.04	-3.08	-2.72	-2.61	0.27	-2.10	-2.56	-3.07	-3.54	-3.78	-2.98	-3.15	-2.56	-3.34	-2.77	-3.15	-2.62	-3.38	-2.12	-2.08	-1.71	-4.08	-3.35	-3.22	0.11720980
Phe-Ser	-2.21	-2.27	-3.04	1.59	-3.59	-2.71	-2.85	-3.25	-2.34	-2.60	-2.60	-3.10	-2.80	-2.80	-2.63	0.18	-2.01	-2.36	-3.21	-3.56	-3.82	-3.40	-3.31	-2.08	-3.54	-2.64	-2.42	-2.15	-3.02	-2.18	-1.95	-1.93	-3.63	-2.60	-2.82	0.11731636
Butyric Acid	-2.02	-2.10	-2.77	-3.18	-3.11	-2.69	-2.15	-2.91	-2.74	-3.24	-3.11	-3.59	-2.51	-2.67	-2.37	-1.52	-2.24	-2.85	-3.63	-2.79	-2.38	-3.57	-2.99	-4.61	-2.70	-3.47	-1.93	-3.35	-2.57	-3.16	-3.94	-3.01	-2.05	-1.91	-2.95	0.11760631
Lys-Ala	-1.94	-1.91	-2.76	-2.31	-2.07	-2.71	-2.64	-2.97	-1.84	-2.10	-2.10	-2.82	-2.40	-2.56	-2.14	-1.52	-1.76	-2.30	-3.52	-3.13	-3.41	-2.79	-2.52	-1.86	-3.05	-2.44	-1.92	-1.91	-2.90	-2.38	-1.87	-1.70	-3.22	-2.08	-2.55	0.12346361
Thr-Ala	-1.94	-1.97	-2.63	-1.89	-2.76	-2.62	-2.56	-2.75	-1.46	-1.89	-1.89	-2.83	-1.98	-2.06	-1.97	-1.35	-1.39	-1.73	-2.64	-3.17	-3.74	-2.87	-2.92	-2.03	-3.16	-2.17	-1.61	-1.55	-2.41	-1.85	-1.42	-1.75	-2.83	-2.40	-2.34	0.12373488
Phe-Ile	-2.46	-2.40	-2.94	-2.55	-3.12	-2.57	-2.52	-3.74	-1.73	-2.45	-2.45	-3.02	-2.81	-2.41	-2.98	0.23	-2.15	-2.88	-3.51	-3.37	-3.74	-3.05	-3.94	-2.48	-3.11	-2.52	-3.07	-1.71	-3.15	-2.38	-2.22	-1.52	-2.82	-3.18	-3.40	0.12446334

Maltose	0.98	0.40	0.18	1.36	1.07	1.35	1.40	1.06	0.95	0.23	0.89	0.08	1.43	1.44	1.01	1.11	1.25	1.16	-0.26	1.28	1.27	0.86	1.13	-0.09	0.15	1.24	0.75	0.63	0.99	0.51	0.89	0.84	-3.05	0.72	1.17	0.12498816
Val-His	-1.34	-1.65	-2.71	-2.68	-3.34	-2.63	-2.53	-3.44	-1.20	-1.82	-1.82	-2.53	-2.06	-2.00	-1.78	-0.96	-1.68	-2.81	-3.74	-3.21	-3.64	-3.13	-2.97	-2.38	-3.08	-1.71	-1.69	-1.35	-3.44	-2.01	-1.59	-1.33	-2.58	-2.99	-2.86	0.12570018
Homoserine	-2.31	-1.79	-2.37	-1.92	-2.88	-2.58	-2.43	-2.78	-1.45	-2.18	-1.72	-2.43	-1.73	-1.75	-1.64	-1.17	-1.17	-1.74	-2.66	-2.90	-3.88	-2.45	-2.83	-1.51	-2.70	-2.13	-1.93	-1.89	-2.68	-1.60	-1.71	-1.29	-2.41	-2.72	-2.03	0.12689756
Val-Pro	-1.46	-2.06	-2.79	-2.98	-3.64	-2.51	-2.72	-3.54	-1.16	-2.05	-2.05	-2.27	-2.01	-2.04	-1.83	-1.15	-1.61	-2.86	-3.64	-3.38	-3.64	-3.27	-3.10	-1.30	-2.94	-1.99	-2.31	-1.29	-3.70	-1.99	-2.15	-1.35	-3.40	-3.22	-2.92	0.12975857
Adenosine	0.74	0.87	1.06	1.24	0.46	0.94	1.12	0.79	0.40	0.04	0.54	-0.24	0.86	0.96	0.77	0.62	0.48	1.09	0.01	0.95	1.14	0.12	0.49	-1.29	-0.31	1.03	0.85	0.76	1.11	0.52	0.40	0.58	0.16	0.64	0.33	0.13175032
Val-Val	-1.51	-2.16	-3.07	-3.02	-3.24	-2.64	-2.81	-3.57	-1.36	-1.76	-1.76	-2.46	-1.84	-1.86	-1.83	-1.04	-1.42	-2.41	-3.22	-3.43	-3.82	-3.20	-2.93	-2.24	-3.16	-2.18	-1.85	-1.18	-3.56	-1.91	-1.55	-1.34	-3.02	-3.21	-2.65	0.13291773
Tyrosine	-2.33	-1.61	-2.22	-2.51	-3.56	-2.54	-2.71	-2.81	-1.37	-2.11	-2.35	-2.13	-1.92	-1.98	-2.79	-1.61	-1.49	-2.59	-3.52	-3.47	-3.72	-3.11	-3.01	-2.09	-2.91	-2.18	-2.61	-1.62	-2.39	-1.81	-1.67	-1.29	-3.63	-2.63	-2.61	0.13312399
Lys-Thr	-1.25	-1.37	-1.47	-1.55	-3.13	-1.56	-1.32	-1.93	-0.99	-1.34	-1.34	-1.70	-0.49	-0.58	-1.13	-0.64	-0.58	-0.87	-2.47	-1.81	-2.19	-1.47	-1.01	-1.11	-1.51	-2.12	-1.35	-0.85	-1.13	-1.76	-0.85	-1.72	-2.65	-1.26	-1.83	0.13405520
Ile-Gly	-3.51	-2.16	-2.56	-2.24	-3.56	-2.43	-2.47	-3.16	-1.22	-2.05	-2.05	-2.64	-2.01	-2.20	-2.34	-1.56	-1.61	-1.98	-3.49	-3.63	-3.97	-3.46	-3.17	-2.29	-3.01	-2.44	-1.86	-2.13	-3.01	-1.94	-1.53	-1.46	-2.61	-2.78	-2.72	0.13428212
Sorbitol	-2.09	-1.43	-2.56	-1.47	-3.04	-2.73	-2.06	-2.11	-1.25	-1.80	-1.60	-2.25	-1.89	-1.43	-2.06	-0.81	-0.89	-2.18	-3.31	-3.12	-3.57	-2.40	-2.24	-1.69	-2.40	-1.66	-1.87	-2.00	-2.74	-1.72	-1.04	-0.86	-3.40	-2.10	-1.87	0.13479594
Serine	-2.52	-1.87	-2.15	-1.34	-2.50	-2.40	-2.66	-2.40	-1.35	-2.25	-1.82	-2.44	-2.08	-1.99	-1.92	-1.34	-1.41	-1.92	-2.86	-3.35	-3.59	-3.02	-3.27	-1.70	-2.80	-2.61	-1.81	-1.12	-1.73	-2.10	-1.69	-1.25	-2.63	-2.05	-2.21	0.13547829
Thymidine	-0.64	-1.47	-1.40	-1.19	-1.82	-1.03	-0.65	-1.54	-2.60	-3.15	-2.17	-3.22	-1.86	-1.96	-1.56	-1.03	-2.66	-0.87	-2.03	-1.27	-1.61	-1.45	-1.18	-1.69	-1.99	-0.66	-0.33	-1.12	-0.67	-1.77	-0.52	-1.54	-2.44	-0.57	-2.02	0.13732318
Ile-Pro	-3.08	-1.90	-2.60	-1.84	-3.44	-2.51	-2.34	-3.02	-1.15	-1.67	-1.67	-2.16	-1.87	-2.37	-2.18	-1.52	-1.63	-2.39	-3.49	-3.49	-3.68	-3.16	-2.94	-1.44	-2.73	-2.14	-2.47	-1.82	-2.91	-2.37	-1.61	-1.13	-2.43	-2.43	-2.80	0.13871120
Ile-Ala	-2.50	-2.01	-2.43	-1.79	-3.21	-2.33	-2.40	-2.62	-1.33	-1.78	-1.78	-2.57	-1.91	-2.03	-2.13	-1.47	-1.70	-1.99	-3.13	-3.44	-3.74	-2.95	-3.02	-1.87	-2.84	-2.03	-2.02	-1.24	-2.20	-1.87	-1.86	-1.62	-2.34	-2.37	-2.39	0.14397550
Lys-Ser	-2.38	-1.81	-2.69	-2.26	-3.61	-2.75	-2.64	-3.04	-1.66	-2.10	-2.10	-2.83	-2.27	-2.16	-2.01	-1.41	-1.41	-2.13	-3.49	-3.51	-4.06	-2.62	-2.20	-2.29	-2.97	-2.45	-1.95	-1.99	-2.86	-2.27	-1.43	-1.73	-3.68	-2.52	-2.56	0.14567608
Met-Glu	-3.08	-1.60	-2.99	-2.70	-3.44	-2.70	-2.67	-3.72	-1.31	-2.34	-2.34	-2.20	-2.87	-2.51	-2.82	-2.49	-2.15	-2.88	-3.76	-3.61	-3.90	-3.24	-3.17	-1.94	-2.99	-3.12	-3.04	-2.07	-3.37	-2.11	-2.27	-1.37	-3.74	-2.80	-3.40	0.14690202
Val-Met	-1.15	-2.02	-3.15	-2.29	-3.24	-2.56	-2.74	-3.78	-1.54	-2.16	-2.16	-2.68	-2.40	-2.02	-1.92	-1.06	-1.88	-2.56	-3.31	-3.72	-3.94	-3.17	-2.99	-1.67	-3.04	-2.40	-1.86	-1.78	-3.38	-1.81	-1.59	-1.56	-3.35	-2.90	-2.99	0.14727041
Thr-Asp	-2.82	-1.81	-2.77	-1.99	-3.51	-2.73	-2.77	-3.16	-1.28	-1.92	-1.92	-2.71	-2.03	-1.95	-2.29	-1.49	-1.45	-2.20	-3.59	-3.41	-3.64	-3.22	-3.13	-2.19	-3.32	-2.18	-2.27	-1.54	-2.67	-2.00	-1.16	-1.42	-3.17	-2.90	-2.76	0.14736791
Tyr-Lys	-0.67	-1.85	-2.18	-1.65	-2.65	-1.89	-1.54	-1.86	-1.55	-1.82	-1.82	-2.37	-1.12	-1.23	-1.49	-1.28	-1.37	-1.34	-2.95	-2.51	-2.84	-1.93	-1.38	-1.37	-2.06	-1.00	-2.01	-1.21	-2.36	-1.82	-1.09	-1.87	-3.00	-1.26	-2.27	0.14749433
Threonine	-2.82	-1.75	-2.46	-1.91	-2.92	-2.49	-2.55	-2.24	-1.36	-2.06	-1.87	-2.36	-2.01	-1.77	-1.93	-1.48	-1.31	-2.20	-3.22	-3.34	-3.51	-3.29	-3.07	-1.53	-2.93	-2.12	-2.33	-0.88	-2.16	-1.83	-1.73	-1.30	-2.87	-2.34	-2.50	0.14809682
Ala-Ile	-2.08	-1.87	-2.40	-1.83	-2.67	-2.28	-1.99	-2.81	-1.42	-2.54	-1.88	-2.51	-1.97	-2.20	-1.96	-1.28	-1.47	-1.56	-2.56	-2.91	-4.13	-2.46	-2.13	-2.53	-2.16	-2.92	-1.63	-1.45	-1.98	-2.03	-1.77	-1.83	-2.50	-2.08	-2.24	0.14903186
Ala-His	-2.10	-2.02	-2.44	-1.94	-2.77	-2.35	-2.26	-2.66	-1.65	-2.55	-1.84	-3.07	-2.18	-2.29	-2.15	-1.23	-1.37	-1.99	-3.08	-3.41	-3.74	-3.20	-3.28	-1.84	-3.29	-2.03	-2.10	-1.60	-1.42	-2.12	-1.36	-1.86	-3.10	-2.24	-2.37	0.15945105
Lactic Acid	-0.23	-1.39	-1.68	-0.95	-1.14	-0.42	-0.72	-1.25	-0.82	-1.74	-1.11	-2.05	-1.10	-1.40	-0.80	-0.07	-0.20	0.05	-1.11	-0.81	-0.21	-0.59	-0.12	-0.50	-1.65	-0.77	-0.28	-0.33	-0.04	-0.84	-0.18	-0.60	-2.34	-0.13	-0.61	0.16012946
Asp-Lys	-1.96	-1.97	-2.58	-2.12	-3.34	-2.60	-2.74	-2.70	-1.22	-2.18	-1.95	-2.35	-1.84	-2.02	-2.47	-1.47	-1.80	-2.67	-3.35	-3.44	-3.84	-3.18	-2.87	-1.55	-2.78	-2.10	-3.08	-1.39	-2.59	-1.51	-1.83	-1.38	-3.34	-2.21	-2.82	0.16837037
Ser-Ser	-3.13	-1.87	-2.29	-1.68	-2.94	-2.87	-2.41	-2.66	-1.40	-1.66	-1.66	-2.55	-1.96	-1.85	-1.78	-1.55	-1.41	-1.83	-2.66	-3.34	-3.57	-3.21	-3.24	-1.83	-3.10	-2.16	-1.52	-1.40	-1.97	-1.94	-1.97	-1.51	-2.69	-1.99	-2.39	0.17440440
Glutamic Acid	-2.34	-2.18	-2.43	-1.84	-2.88	-2.52	-2.74	-2.55	-1.61	-2.51	-1.90	-2.83	-2.02	-2.06	-2.03	-1.38	-1.45	-2.16	-3.28	-3.49	-3.74	-3.15	-2.87	-1.54	-3.05	-1.77	-2.40	-1.54	-2.68	-2.09	-1.85	-1.64	-2.32	-2.13	-2.44	0.17578596
Ser-Asn	-2.22	-1.78	-2.13	-1.49	-3.15	-2.41	-2.51	-2.72	-1.22	-1.76	-1.76	-2.41	-1.86	-2.03	-1.45	-1.43	-1.37	-1.76	-2.93	-3.44	-3.54	-3.32	-3.27	-1.48	-2.90	-1.70	-1.69	-1.33	-2.02	-1.94	-1.46	-1.17	-2.53	-1.97	-2.21	0.17766537
Thr-Phe	-2.56	-2.40	-3.15	-2.03	-3.22	-2.79	-2.54	-3.37	-2.32	-2.51	-2.51	-3.35	-3.00	-2.79	-2.03	-1.83	-2.47	-2.36	-3.27	-3.61	-3.46	-3.28	-3.29	-2.17	-3.66	-2.40	-2.45	-2.43	-3.12	-2.34	-1.87	-1.83	-3.63	-2.95	-3.20	0.17768100
Thr-Arg	-2.41	-1.91	-2.67	-2.10	-3.63	-2.60	-2.61	-3.11	-1.62	-2.01	-2.01	-3.02	-2.36	-2.27	-2.40	-1.73	-1.85	-2.09	-3.41	-3.68	-4.01	-3.51	-3.32	-1.92	-3.28	-1.94	-1.95	-1.41	-2.69	-2.08	-1.79	-1.63	-3.08	-2.92	-2.87	0.17889944
Glu-Gly	-2.09	-1.81	-2.87	-2.70	-3.54	-2.60	-2.82	-3.25	-1.47	-2.01	-2.18	-2.16	-2.41	-2.60	-2.57	-1.63	-1.40	-2.75	-3.31	-4.47	-3.84	-2.46	-2.18	-2.56	-2.77	-2.95	-2.71	-1.67	-3.12	-1.80	-1.83	-1.28	-2.86	-3.52	-2.80	0.18084051
AcetyMannosamine	0.56	-1.83	-2.83	-2.03	-3.43	-2.47	-2.10	-2.40	-1.33	-2.21	-1.83	-2.40	-1.97	-1.75	-2.50	-1.69	-1.19	-2.40	-3.37	-3.07	-3.24	-2.87	-2.82	-1.31	-2.90	-1.54	-2.70	-2.22	-2.63	-1.92	-1.37	-0.97	-2.53	-2.27	-2.14	0.18168657
Histidine	-2.20	-1.66	-2.45	-1.95	-3.70	-3.01	-2.69	-2.72	-1.43	-2.24	-1.84	-2.49	-1.84	-1.72	-2.23	-1.50	-1.24	-2.40	-3.02	-3.49	-3.78	-3.37	-3.13	-1.95	-2.90	-2.01	-2.56	-1.24	-3.10	-1.99	-1.77	-1.17	-2.73	-1.72	-2.78	0.18202605
Phe-Gly	-2.25	-2.47	-2.98	-2.94	-3.38	-2.80	-2.64	-3.44	-2.30	-2.78	-2.78	-3.25	-3.02	-2.61	-2.61	0.12	-2.52	-2.85	-3.46	-3.28	-3.59	-3.10	-3.88	-2.45	-3.56	-2.46	-2.32	-2.30	-3.05	-2.57	-2.22	-1.84	-3.92	-3.10	-3.23	0.18225771
Thr-Pro	-2.81	-1.73	-2.71	-1.71	-3.46	-2.51	-2.78	-2.56	-1.20	-1.82	-1.82	-2.51	-2.03	-1.97	-2.01	-1.76	-1.69	-2.37	-3.46	-3.41	-3.78	-3.18	-3.06	-2.11	-2.55	-1.98	-2.54	-1.60	-1.64	-1.88	-1.58	-1.37	-2.98	-2.53	-2.80	0.18260184
Asp-Gly	-1.86	-2.06	-2.74	-1.78	-3.70	-2.87	-2.89	-2.66	-1.40	-2.36	-2.10	-2.55	-1.93	-1.86	-1.76	-1.67	-1.69	-2.47	-3.22	-3.41	-3.35	-3.11	-2.98	-1.57	-2.90	-2.18	-2.44	-1.78	-2.94	-1.72	-1.63	-1.55	-3.34	-2.24	-2.75	0.18317455
Phe-Phe	-2.74	-2.37	-3.32	-2.91	-3.54	-2.92	-2.93	-3.90	-2.43	-2.69	-2.69	-3.15	-3.10	-2.86	-2.86	0.06	-2.62	-2.63	-3.41	-3.64	-3.82	-3.29	-3.21	-1.69	-3.52	-2.80	-3.49	-2.70	-3.31	-2.31	-2.60	-1.79	-3.59	-3.90	-3.42	0.19042976
Lys-Met	-2.63	-1.76	-2.77	-1.85	-3.06	-2.54	-2.81	-3.02	-1.64	-2.09	-2.09	-2.85	-2.22	-2.51	-2.26	-1.43	-1.58	-1.88	-3.22	-3.66	-3.86	-3.08	-3.01	-1.98	-3.04	-2.00	-1.92	-2.19	-2.98	-1.65	-1.77	-1.33	-3.52	-2.06	-2.54	0.19075343
Met-Phe	-3.32	-2.06	-2.99	-2.54	-2.56	-2.91	-2.61	-3.54	-2.28	-2.40	-2.40	-2.94	-2.84	-2.88	-2.81	-2.10	-2.15	-2.64	-3.35	-3.40	-3.61	-3.25	-3.16	-2.23	-3.37	-2.95	-3.02	-1.80	-3.34	-2.46	-2.12	-1.63	-3.72	-2.91	-3.10	0.19089008
Lys-Phe	-3.20	-1.94	-3.18	-3.35	-3.20	-2.70	-2.84	-3.22	-2.25	-2.51	-2.51	-3.08	-2.89	-2.76	-2.67	-1.79	-1.98	-2.38	-3.78	-3.78	-3.74	-3.43	-3.37	-2.17	-3.54	-2.67	-2.48	-2.24	-3.61	-1.99	-2.28	-1.65	-3.51	-3.20	-3.01	0.19090501
His-His	-2.67	-1.75	-2.63	-2.06	-3.24	-2.75	-2.64	-3.12	-1.34	-2.06	-2.06	-2.34	-1.94	-2.28	-2.31	-1.51	-1.38	-2.53	-2.86	-3.49	-3.94	-3.41	-3.29	-2.22	-2.82	-2.72	-1.78	-1.35	-2.74	-1.92	-1.75	-1.07	-3.13	-2.38	-2.76	0.19217823
Butyric Acid	-0.83	-1.51	-2.02	-1.60	-1.68	-1.38	-0.63	-1.78	-1.24	-1.97	-1.27	-2.34	-1.69	-1.71	-1.26	-0.72	-0.87	-0.62	-1.76	-0.36	-2.34	-1.01	-0.21	-1.10	-1.97	-0.91	-0.75	-0.80	-0.59	-1.44	-0.42	-1.16	-2.33	-0.84	-1.28	0.19544719
Trehalose	-2.10	-1.15	-2.51	-3.05	-3.15	-2.66	-1.05	-2.86	-1.07	-1.94	-1.45	-1.88	-0.71	-1.48	-1.62	-1.03	-0.92	-1.97	-3.02	-2.75	-3.35	-2.35	-1.93	-1.49	-2.29	-1.99	-1.79	-1.87	-1.99	-1.46	-1.10	-0.98	-3.78	-2.69	-1.74	0.20145088
Ala-Lys	-2.34	-2.04	-2.68	-2.26	-2.97	-2.51	-2.80	-2.73	-1.61	-2.54	-2.13	-2.59	-2.31	-2.34	-1.94	-1.35	-1.44	-2.11	-2.94	-2.72	-3.94	-2.69	-2.76	-2.26	-2.61	-2.04	-2.20	-1.86	-2.72	-1.97	-1.89	-1.74	-3.18	-2.38	-2.36	0.20249953
Arg-Asp	-3.00	-1.90	-2.53	-2.27	-3.43	-2.45	-2.74	-2.84	-1.34	-2.08	-1.84	-2.40	-1.98	-1.95	-2.73	-1.32	-1.41	-2.55	-3.40	-3.37	-3.74	-3.25	-2.87	-1.38	-2.80	-1.86	-2.40	-1.83	-3.12	-1.88	-1.72	-1.02	-3.21	-3.25	-2.74	0.20278337
Val-Asp	-2.40	-2.13	-3.04	-2.15	-3.57	-2.47	-2.66	-3.57	-1.33	-1.65	-1.65	-2.48	-2.08	-1.87	-2.07	-2.45	-1.56	-2.44	-4.04	-3.66	-3.88	-3.44	-3.15	-2.36	-2.74	-1.63	-1.97	-2.00	-2.71	-1.86	-1.35	-1.22	-3.76	-2.24	-3.11	0.20589451
Ala-Asp	-2.53	-2.18	-2.77	-1.94	-3.32	-2.51	-2.43	-2.86	-1.45	-2.53	-2.00	-2.81	-2.03	-2.27	-2.07	-1.76	-1.57	-2.27	-3.25	-3.44	-3.66	-3.18	-3.17	-1.63	-2.93	-2.22	-2.29	-1.52	-2.05	-2.30	-1.78	-1.79	-3.32	-2.39	-2.50	0.20909473
Glucose-6-P	-2.32	-0.97	-1.61	-0.70	-3.29	-2.78	-0.78	-2.13	-1.37	-1.56	-1.23	-1.70	-0.46	-0.42	-1.00	-0.69	-0.57	-1.24	-2.15	-1.86	-3.59	-2.72	-1.80	-1.14	-2.08	-0.96	-1.35	-0.75	-1.30	-1.22	-1.30	-0.94	-2.73	-1.21	-2.14	0.21223791
Val-Phe	-1.17	-2.42	-3.06	-3.20	-3.56	-2.75	-2.63	-4.01	-2.18	-2.38	-2.38	-2.94	-2.85	-2.49	-2.40	-1.19	-2.19	-3.28	-3.61	-3.61	-3.68	-3.25	-3.10	-1.92	-3.37	-2.99	-2.33	-1.99	-3.78	-1.89	-2.21	-1.55	-3.90	-3.41	-3.34	0.21245516
Asp-Phe	-2.63	-2.31	-3.12	1.66	-3.27	-2.90	-2.99	-3.64	-2.10	-2.47	-2.51	-3.01	-2.80	-2.68	-2.71	-2.10	-2.27	-3.12	-3.54	-3.63	-3.59	-3.34	-3.27	-1.83	-3.11	-2.23	-3.38	-2.24	-3.34	-1.72	-2.63	-1.51	-3.28	-3.20	-3.37	0.21806549
Pro-Hyp	-2.47	-2.04	-2.46	-1.89	-3.35	-2.59	-2.63	-2.93	-1.42	-1.97	-1.97	-2.53	-1.86	-1.76	-2.24	-1.45	-1.48	-2.17	-3.40	-3.44	-3.90	-3.22	-2.91	-1.67	-3.08	-1.77	-2.19	-1.33	-2.70	-1.93	-1.34	-1.37	-3.11	-2.01	-2.71	0.21902036
Glucose	0.26	0.07	-0.27	0.64	0.21	0.93	0.66	-0.04	0.24	0.09	0.09	-0.26	0.43	0.89	0.30	0.62	0.54	0.95	0.23	0.59	0.48	0.32	0.71	-0.22	-0.21	0.80	0.47	-0.11	0.07	0.16	0.18	-0.24	-0.40	-0.03	0.55	0.22444059
Asp-Ala	-2.49	-2.17	-2.86	-2.35	-2.98	-2.68	-2.70	-3.01	-1.60	-2.55	-2.07	-2.86	-2.18	-2.36	-2.17	-1.44	-1.36	-2.15	-3.21	-3.38	-3.68	-2.75	-2.78	-2.30	-2.63	-2.02	-1.74	-2.38	-2.29	-2.16	-1.72	-1.76	-3.82	-2.69	-2.45	0.22664070
Met-His	-2.84	-1.97	-2.67	-2.30	-3.59	-2.70	-2.65	-3.11	-1.73	-2.10	-2.10	-3.06	-2.46	-2.78	-2.40	-1.41	-1.53	-1.83	-3.24	-3.76	-3.56	-3.06	-3.17	-2.20	-3.12	-2.30	-2.05	-1.92	-3.11	-1.83	-1.98	-1.55	-3.82	-2.18	-2.64	0.22887734
Mannitol	0.65	-2.01	-2.58	-1.88	-3.25	-2.35	-1.16	-2.34	-1.41	-2.05	-1.85	-2.22	-1.86	-1.71	-2.21	-1.47	-1.24	-2.24	-3.44	-3.10	-3.52	-2.90	-2.44	-1.25	-2.51	-1.59	-2.01	-1.49	-2.04	-1.95	-0.90	-0.98	-1.90	-2.71	-2.22	0.23042904
Sucrose	0.51	-1.78	-2.99	-2.32	-3.51	-2.52	-2.37	-2.91	-1.24	-2.40	-2.08	-2.46	-1.99	-1.78	-2.06	-1.38	-1.34	-2.40	-3.54	-3.40	-3.72	-2.53	-2.31	-1.95	-2.71	-1.84	-2.47	-2.22	-2.88	-1.81	-1.25	-0.98	-2.34	-2.45	-2.15	0.23589497
Ile-Met	-3.17	-1.95	-2.92	-1.68	-3.25	-2.79	-2.37	-3.63	-1.47	-1.91	-1.91	-2.58	-2.04	-2.34	-2.39	-1.98	-1.77	-2.90	-3.44	-3.46	-3.64	-2.99	-3.13	-1.78	-2.76	-2.10	-2.59	-1.81	-3.40	-2.10	-1.65	-1.28	-3.74	-2.70	-2.89	0.23787056
Ile-Leu	-1.44	-1.64	-2.47	-1.44	-2.69	-1.45	-1.42	-2.47	-1.02	-1.84	-1.84	-2.29	-1.75	-1.71	-1.56	-0.95	-1.30	-1.30	-2.70	-2.89	-3.38	-2.26	-2.92	-1.40	-1.97	-2.02	-1.63	-1.30	-1.50	-1.72	-0.99	-1.16	-2.47	-0.67	-2.31	0.24449794
Val-Leu	-1.46	-1.65	-2.41	-1.41	-2.10	-0.76	-1.30	-2.47	-1.26	-1.83	-1.83	-2.48	-1.92	-2.01	-1.53	-0.86	-1.56	-1.01	-2.73	-3.11	-3.84	-2.30	-1.84	-1.14	-2.36	-1.42	-1.14	-1.47	-1.10	-1.81	-0.98	-1.60	-2.27	-1.37	-2.28	0.24811495
Ornithine	-3.04	-2.01	-2.63	-1.84	-3.22	-2.56	-2.81	-2.94	-1.40	-2.22	-1.92	-2.54	-1.86	-1.90	-2.26	-1.55	-1.45	-2.22	-3.38	-3.18	-3.63	-3.10	-2.82	-1.48	-2.65	-2.19	-2.62	-1.87	-1.91	-2.09	-1.83	-1.45	-3.22	-2.14	-2.74	0.24932450
Ile-His	-3.24	-2.01	-2.70	-1.76	-3.20	-2.62	-2.53	-3.68	-1.19	-1.91	-1.91	-2.55	-2.28	-2.53	-2.76	-1.69	-1.68	-2.54	-3.66	-3.68	-3.88	-3.27	-3.46	-1.89	-2.41	-2.27	-2.92	-1.58	-3.51	-1.85	-1.21	-1.25	-2.68	-3.24	-2.85	0.24997532
Ser-Pro	-2.85	-1.79	-2.59	-2.01	-2.95	-2.62	-2.63	-2.53	-1.24	-1.95	-1.95	-2.21	-2.05	-1.99	-2.03	-1.69	-1.63	-2.27	-3.32	-3.40	-3.51	-3.11	-3.11	-2.01	-2.56	-1.71	-1.94	-1.48	-1.62	-2.20	-1.60	-1.47	-3.07	-2.22	-2.60	0.25153566
Leu-Phe	-1.25	-2.24	-3.01	-2.56	-2.32	-2.02	-2.10	-3.37	-2.16	-2.53	-2.53	-3.32	-2.80	-2.81	-2.38	-1.05	-2.05	-1.20	-2.88	-2.86	-3.40	-2.42	-1.94	-1.27	-3.27	-1.73	-1.34	-1.87	-0.93	-1.79	-1.42	-1.75	-2.27	-1.10	-2.40	0.25196901
Ser-Gly	-2.18	-2.10	-2.56	-1.79	-3.38	-2.63	-2.65	-2.62	-1.74	-2.15	-2.15	-2.85	-2.08	-2.10	-1.95	-1.38	-1.43	-1.70	-2.94	-3.27	-3.74	-3.32	-2.66	-1.74	-2.61	-2.08	-1.68	-1.78	-2.46	-2.05	-1.87	-1.62	-3.04	-1.88	-2.43	0.25898974
Val-Ser	-1.20	-1.94	-2.77	-2.97	-3.25	-2.59	-2.50	-2.91	-1.30	-2.20	-2.20	-2.66	-2.01	-2.10	-1.97	-1.20	-1.52	-2.19	-3.18	-3.41	-3.66	-4.92	-3.15	-1.67	-2.94	-2.13	-1.68	-1.51	-2.41	-1.95	-1.27	-1.47	-2.78	-2.08	-2.43	0.26067167
His-Pro	-2.10	-1.65	-2.41	-2.17	-3.21	-2.71	-2.55	-3.51	-1.24	-1.86	-1.86	-2.47	-2.11	-2.18	-2.27	-1.45	-1.57	-2.42	-3.25	-3.04	-3.70	-2.89	-2.87	-1.39	-2.60	-2.19	-2.35	-1.56	-2.84	-2.24	-1.60	-1.51	-2.72	-2.06	-2.74	0.27151724
Ala-Arg	-2.21	-1.95	-2.73	-2.64	-2.69	-2.26	-2.09	-3.01	-1.82	-2.56	-2.08	-2.93	-2.37	-2.35	-2.08	-1.23	-1.57	-1.57	-2.47	-2.34	-3.41	-2.16	-1.45	-1.24	-2.27	-2.37	-1.75	-1.14	-1.51	-2.18	-1.21	-1.99	-2.89	-1.70	-2.25	0.27303821
Ala-Glu	-3.01	-1.84	-2.78	-2.10	-3.44	-2.53	-2.56	-2.89	-1.26	-1.83	-2.13	-2.04	-2.16	-2.28	-2.62	-2.66	-2.26	-2.76	-3.49	-3.40	-3.64	-3.20	-3.18	-2.08	-3.17	-2.20	-2.28	-1.51	-2.38	-2.61	-1.75	-1.19	-3.40	-2.58	-2.77	0.27476645
Leu-Tyr	-1.20	-1.82	-1.97	-1.43	-3.63	-1.54	-1.58	-1.94	-1.22	-1.68	-1.68	-2.30	-1.08	-1.14	-1.27	-0.76	-0.96	-0.99	-2.69	-2.19	-3.20	-1.93	-1.49	-2.03	-2.33	-1.30	-1.37	-1.46	-0.71	-1.70	-1.37	-1.67	-2.30	-0.94	-2.02	0.27785694
Galactose	0.44	0.45	0.56	0.91	-0.06	1.13	0.97	0.42	0.32	-0.69	-0.04	-1.14	0.64	0.71	0.41	0.49	0.30	0.37	-0.29	0.31	0.81	0.49	0.77	-0.14	-0.10	0.42	0.70	0.37	0.63	-0.47	0.53	-0.12	-1.51	0.32	-0.55	0.27867668
Met-Ile	-3.27	-1.94	-2.79	-2.45	-3.52	-2.58	-2.36	-3.61	-1.29	-1.80	-1.80	-2.48	-2.11	-2.29	-2.34	-1.69	-1.38	-2.37	-3.24	-3.70	-3.78	-2.78	-3.10	-2.70	-2.27	-2.56	-2.35	-1.63	-3.49	-1.72	-1.31	-1.17	-3.64	-2.51	-2.52	0.27872959
Arg-Ile	-0.68	-1.66	-2.18	-1.25	-2.13	-1.82	-1.60	-2.22	-1.38	-1.79	-1.80	-2.59	-1.70	-1.65	-1.68	-0.95	-1.18	-1.70	-2.40	-2.06	-3.37	-1.74	-1.23	-2.92	-2.03	-2.86	-1.54	-0.67	-0.91	-1.63	-1.51	-1.23	-2.57	-1.61	-1.94	0.28138782
Met-Met	-3.17	-2.13	-3.07	-3.20	-1.55	-2.48	-2.64	-3.56	-1.72	-2.18	-2.18	-2.86	-2.40	-2.45	-2.32	-1.43	-1.59	-2.18	-3.00	-3.24	-3.68	-3.02	-3.04	-1.95	-2.78	-2.81	-2.16	-1.54	-3.18	-2.18	-1.82	-1.57	-3.59	-2.43	-2.75	0.28184387
Met-Val	-3.59	-1.98	-3.06	-2.40	-2.94	-2.61	-2.87	-3.76	-1.69	-2.10	-2.10	-2.53	-2.34	-2.47	-2.15	-1.61	-1.56	-2.84	-3.10	-3.68	-3.78	-3.21	-3.13	-1.75	-2.72	-2.37	-2.38	-2.05	-3.46	-1.88	-2.18	-1.34	-3.82	-2.75	-2.86	0.28216001
Arg-Phe	-1.43	-2.18	-2.92	-2.05	-2.75	-2.44	-2.33	-2.78	-2.05	-2.77	-2.44	-3.21	-2.56	-2.65	-2.06	-1.25	-1.95	-1.87	-2.51	-2.84	-3.20	-2.60	-1.70	-1.15	-2.74	-2.04	-1.43	-1.66	-1.27	-2.55	-1.02	-1.85	-2.97	-1.51	-2.51	0.28585685
Uridine	-0.73	-0.87	-1.40	-0.72	-1.74	-1.18	-0.53	-0.78	-0.66	-1.73	-1.14	-1.96	-1.00	-0.53	-1.16	-0.80	-0.60	-1.05	-3.08	-1.42	-2.53	-2.06	-0.11	-1.26	-0.92	-0.88	-1.23	-1.49	-0.93	-1.19	-0.56	-0.25	-2.08	-0.62	-0.95	0.28848555
Ala-Val	-2.13	-2.04	-2.69	-1.73	-3.06	-2.42	-2.54	-2.50	-1.52	-2.27	-1.79	-2.48	-2.11	-2.11	-1.89	-1.46	-1.61	-2.10	-3.06	-3.38	-3.72	-3.40	-2.90	-1.88	-3.34	-1.91	-1.86	-1.35	-2.01	-1.86	-1.15	-1.44	-2.68	-2.00	-2.42	0.28930423
Phe-Val	-2.55	-1.93	-2.86	-3.24	-3.47	-2.72	-3.84	-3.84	-2.09	-1.79	-1.79	-2.95	-2.94	-2.85	-2.46	0.30	-1.75	-2.50	-2.84	-3.52	-4.32	-3.10	-3.17	-2.04	-2.92	-2.72	-2.40	-2.35	-3.06	-2.02	-2.09	-1.56	-3.97	-3.27	-2.64	0.29013009
Tyr-Gln	-0.49	-0.38	-1.25	-0.71	-2.43	-1.21	-0.92	-1.38	-0.01	-1.20	-1.20	-0.99	-0.17	-0.50	-1.49	-0.62	-1.25	-1.15	-2.06	-1.22	-2.89	-1.34	-0.71	-1.33	-0.63	-0.20	-0.99	-0.59	-0.85	-0.70	-0.66	-1.86	-1.68	-1.19	-1.28	0.29236542
Lys-Ile	-1.60	-1.51	-1.93	-0.97	-2.40	-0.64	-0.63	-1.86	-1.14	-1.52	-1.52	-2.49	-1.42	-1.35	-1.80	-0.97	-1.39	-1.81	-2.78	-2.73	-3.43	-2.20	-1.51	-1.18	-1.93	-1.98	-0.93	-0.66	-1.41	-1.91	-0.66	-1.01	-1.92	-1.22	-2.07	0.29426746
His-Met	-2.47	-1.68	-2.56	-2.27	-3.07	-2.75	-2.64	-3.72	-1.75	-2.20	-2.20	-2.81	-2.40	-2.83	-2.31	-1.15	-1.30	-1.77	-2.97	-3.49	-3.80	-2.27	-2.59	-2.01	-2.76	-2.81	-2.13	-2.27	-3.47	-1.81	-1.28	-1.43	-3.31	-3.05	-2.29	0.29608481
Butyric Acid	-0.32	-1.82	-1.69	-0.22	-1.24	-1.79	0.16	-1.28	-0.32	-1.63	-0.80	-1.78	-1.30	-0.64	-0.56	0.07	-0.01	-0.09	-1.80	-0.58	-1.88	-1.12	-0.43	-1.96	-1.62	-1.51	-0.81	-0.73	-0.66	-0.95	-0.73	-0.07	-1.83	-0.87	-0.78	0.30180096
Asn-Glu	-1.93	-1.45	-2.63	-2.67	-3.06	-2.56	-2.53	-3.15	-0.89	-1.53	-1.74	-1.73	-1.98	-2.12	-2.82	-1.67	-1.42	-2.73	-3.40	-2.27	-3.97	-3.06	-2.99	-1.91	-2.54	-1.29	-2.63	-2.05	-2.45	-1.86	-1.44	-0.94	-3.27	-1.63	-3.31	0.30359472
Glu-Val	-1.34	-1.50	-2.32	-2.38	-3.38	-2.54	-2.57	-3.52	-1.66	-1.75	-3.13	-1.54	-2.56	-2.55	-2.53	-2.43	-2.40	-2.61	-3.37	-3.35	-3.78	-3.00	-2.77	-1.62	-2.52	-2.83	-3.21	-0.66	-3.07	-2.90	-1.43	-1.12	-3.11	-2.93	-3.29	0.30443931
Phenylalanine	-3.38	-2.42	-3.17	1.70	-3.54	-2.95	-2.80	-3.41	-2.28	-3.08	-2.81	-3.12	-3.11	-2.90	-2.92	-2.16	-2.30	-2.66	-3.82	-3.41	-3.51	-3.52	-3.52	-2.05	-3.32	-3.15	-2.83	-1.92	-3.13	-2.44	-2.02	-1.79	-3.68	-3.07	-3.11	0.30501790
Phe-Ala	-2.69	-2.65	-3.21	-2.67	-2.33	-2.80	-2.76	-3.35	-2.60	-2.59	-2.59	-3.41	-2.99	-3.10	-2.65	-1.65	-2.24	-2.50	-3.18	-3.72	-3.78	-3.06	-3.27	-2.04	-3.32	-3.17	-2.15	-2.29	-3.13	-2.21	-2.24	-2.18	-3.57	-2.90	-2.92	0.30809131
Gly-Asp	-1.82	-1.95	-2.78	-2.40	-3.40	-2.70	-2.91	-2.95	-1.35	-2.40	-2.04	-2.39	-2.28	-2.31	-2.40	-1.42	-1.36	-2.33	-2.81	-3.27	-3.80	-2.51	-2.13	-2.62	-2.56	-2.44	-2.25	-1.42	-3.18	-2.08	-1.37	-1.41	-3.80	-2.74	-2.37	0.31544213
Ala-Ser	-2.90	-2.32	-2.60	-1.97	-2.89	-2.43	-2.78	-2.74	-1.63	-2.57	-2.11	-2.78	-2.19	-2.22	-1.94	-1.38	-1.57	-2.11	-2.66	-3.08	-3.56	-3.07	-3.07	-1.49	-3.07	-2.39	-1.96	-1.71	-1.94	-2.13	-1.93	-1.85	-3.64	-2.23	-2.42	0.31818856
Pro-Ser	-2.93	-1.99	-2.52	-1.83	-3.04	-2.61	-2.63	-2.84	-1.37	-1.97	-1.97	-2.56	-1.93	-2.14	-2.04	-1.54	-1.63	-2.30	-3.34	-3.40	-3.57	-3.15	-3.07	-2.71	-2.74	-2.04	-1.95	-1.21	-2.39	-1.97	-1.45	-1.46	-2.17	-1.87	-2.77	0.32316691
Ile-Arg	-1.42	-1.56	-1.72	-1.18	-2.92	-0.54	-1.04	-1.57	-1.05	-1.73	-1.73	-2.49	-1.74	-1.51	-1.50	-0.94	-1.34	-1.52	-2.72	-2.76	-3.29	-1.86	-1.20	-1.26	-2.11	-1.78	-1.29	-0.65	-1.82	-1.55	-0.70	-1.25	-1.80	-1.42	-2.17	0.32467294
Proline	-3.41	-1.89	-2.54	-1.73	-3.56	-2.54	-2.60	-2.46	-1.28	-2.16	-2.06	-2.47	-1.85	-1.77	-2.11	-1.71	-1.57	-2.37	-3.51	-3.31	-3.70	-3.29	-3.34	-1.67	-2.57	-2.24	-2.27	-1.13	-2.13	-2.15	-1.46	-1.31	-3.25	-1.96	-2.69	0.32664453
Gly-Met	-2.79	-2.11	-2.94	-2.39	-3.16	-2.92	-2.62	-3.20	-1.98	-2.43	-2.43	-2.89	-2.59	-2.69	-2.49	-1.58	-1.77	-2.18	-2.88	-3.24	-4.01	-2.88	-3.17	-1.76	-3.01	-2.53	-2.20	-2.13	-3.27	-1.96	-1.99	-1.48	-3.74	-3.08	-2.71	0.33228762
Gly-Leu	-1.71	-2.01	-2.68	-1.85	-1.95	-2.04	-1.89	-2.50	-1.91	-2.27	-2.27	-2.70	-2.42	-2.22	-1.93	-1.08	-1.53	-1.29	-2.50	-2.56	-3.13	-2.34	-1.82	-1.75	-3.05	-2.16	-1.62	-2.06	-1.52	-2.00	-2.01	-1.73	-2.41	-1.80	-2.33	0.33471208
Aspartic Acid	-2.25	-1.94	-2.59	-2.02	-3.05	-2.60	-2.68	-2.61	-1.62	-2.34	-2.02	-2.63	-2.02	-2.12	-2.13	-1.51	-1.56	-1.93	-2.94	-3.28	-3.61	-3.16	-3.00	-1.60	-2.89	-2.16	-2.38	-1.42	-2.49	-2.03	-1.42	-1.58	-1.92	-2.28	-2.47	0.33711856
Thr-Gln	-0.28	-0.56	-1.36	-0.49	-2.37	-1.58	-1.62	-1.51	-0.17	-0.52	-0.52	-0.64	-0.48	-0.51	-0.84	-0.34	-0.94	-0.97	-1.76	-0.52	-2.70	-1.33	-1.31	-1.78	-0.87	-0.51	-0.69	-0.77	-0.63	-0.63	-0.93	-0.61	-1.18	-0.60	-1.31	0.33730544
Glucose	0.40	0.68	0.05	0.63	0.03	0.70	0.77	0.02	0.21	-0.57	0.01	-0.54	0.39	0.36	0.39	0.76	0.60	0.68	0.45	0.47	0.32	0.64	0.81	-0.35	0.11	0.48	0.20	-0.21	0.20	0.21	0.22	-0.01	-1.12	0.04	0.43	0.34170035
Maltotriose	0.57	0.46	0.27	-0.97	0.81	1.10	1.33	0.79	0.45	-0.11	0.74	-0.20	1.32	1.32	0.80	0.62	0.80	0.71	0.24	0.88	1.04	0.33	0.80	-0.17	-0.08	1.06	0.61	0.27	0.76	0.30	0.55	0.67	-2.40	0.51	0.94	0.34264016
Ser-Phe	-2.51	-2.21	-3.28	-2.48	-3.37	-3.00	-2.80	-3.00	-2.31	-2.37	-2.37	-3.02	-3.05	-2.83	-2.21	-1.94	-2.37	-2.11	-3.15	-3.49	-3.63	-3.31	-3.66	-2.92	-3.57	-2.52	-1.86	-1.81	-2.71	-2.14	-2.40	-2.10	-2.99	-2.27	-3.06	0.34428822
Arg-Tyr	-0.70	-1.54	-2.04	-1.59	-2.20	-1.92	-1.60	-2.00	-1.35	-2.08	-1.80	-2.29	-1.13	-1.20	-1.53	-0.91	-1.13	-1.44	-2.53	-2.04	-3.21	-2.03	-1.31	-1.25	-2.20	-1.40	-1.27	-1.18	-1.11	-2.28	-0.56	-1.43	-2.60	-1.51	-2.16	0.34676365
Stachyose	-2.09	-1.65	-2.39	-2.10	-3.01	-2.61	-0.46	-2.11	-1.23	-2.02	-1.69	-2.50	-1.94	-1.81	-2.04	-1.28	-1.38	-2.17	-3.41	-3.16	-3.44	-2.27	-1.54	-1.33	-2.21	-1.76	-1.91	-1.90	-2.70	-1.91	-1.16	-0.97	-2.36	-2.05	-2.11	0.34743725
Met-Thr	-3.31	-1.97	-3.00	-1.88	-3.16	-2.77	-2.56	-3.38	-1.75	-2.21	-2.21	-2.62	-2.32	-2.16	-2.32	-1.42	-2.03	-2.33	-2.74	-3.41	-3.72	-3.10	-2.85	-1.99	-2.69	-2.55	-2.18	-1.85	-3.04	-2.01	-1.81	-1.60	-3.41	-2.56	-2.67	0.35078984
Ala-Thr	-3.27	-2.16	-2.84	-1.71	-2.74	-2.46	-2.61	-2.61	-1.69	-2.58	-2.06	-2.74	-1.81	-1.92	-1.74	-1.36	-1.42	-1.89	-2.81	-3.00	-3.52	-2.79	-2.89	-1.70	-2.76	-2.18	-1.75	-1.79	-2.06	-1.98	-1.86	-1.64	-3.13	-2.29	-2.28	0.35726808
Chondroitin Sulfate	0.60	-1.90	-2.56	-2.11	-3.02	-2.43	-1.84	-2.51	-1.50	-2.39	-2.05	-2.56	-1.93	-1.91	-2.13	-1.56	-1.42	-2.31	-3.49	-3.12	-3.34	-2.72	-2.67	-1.15	-2.84	-2.10	-2.63	-1.57	-1.87	-2.25	-1.19	-1.20	-1.98	-1.37	-2.16	0.35777418
Leu-Val	-1.28	-1.93	-2.47	-1.45	-2.13	-1.04	-1.83	-2.56	-1.28	-1.83	-1.83	-2.75	-2.05	-2.02	-1.88	-1.02	-1.16	-1.15	-2.63	-2.77	-3.64	-1.80	-1.58	-2.26	-2.51	-1.73	-1.49	-1.35	-1.30	-1.69	-1.06	-1.38	-2.51	-1.30	-2.19	0.36175326
Cellobiose	-2.34	-1.88	-3.12	-2.80	-3.35	-2.61	-1.43	-3.28	-1.43	-2.31	-1.50	-2.56	-1.42	-2.16	-2.15	-1.28	-1.44	-2.38	-3.37	-3.06	-3.66	-2.16	-1.86	-1.73	-2.47	-2.03	-2.24	-2.19	-2.74	-2.05	-1.16	-1.32	-4.04	-3.12	-1.99	0.36467105
Gelatin	-2.06	-1.76	-1.98	-1.88	-1.95	-2.39	-2.42	-1.79	-1.49	-1.56	-1.64	-1.96	-1.84	-2.15	-1.42	-1.41	-1.71	-1.67	-2.89	-3.22	-2.94	-3.21	-2.66	-2.05	-2.43	-1.88	-1.17	-1.10	-1.22	-1.73	-1.10	-1.54	-1.46	-1.35	-2.40	0.36646971
MethyGlucoside	-1.99	-1.80	-2.74	-2.22	-3.06	-1.64	-1.70	-2.76	-1.15	-2.18	-1.79	-2.21	-1.94	-1.68	-2.06	-1.40	-1.19	-2.32	-3.44	-2.88	-3.35	-2.60	-2.68	-1.08	-2.82	-1.75	-2.05	-1.75	-2.11	-1.99	-1.28	-0.82	-2.07	-2.57	-1.99	0.36725100
Met-Gln	-0.10	-0.35	-0.61	0.24	-1.93	-1.41	-0.97	-1.07	0.19	-0.32	-0.32	-0.92	-0.32	-0.31	-0.31	-0.33	-1.15	-0.88	-2.08	-0.52	-1.95	-0.91	-0.33	-1.06	-0.68	0.25	-0.69	-0.34	-0.33	-0.21	-0.61	-1.82	-1.09	-0.06	-1.07	0.36834555
AcetyGlucosamine	-2.34	-1.70	-2.47	-2.10	-3.17	-2.56	-0.44	-2.41	-1.30	-2.11	-1.93	-2.40	-1.74	-1.91	-2.11	-1.26	-1.29	-2.16	-3.28	-2.93	-3.59	-2.10	-1.78	-1.60	-2.35	-2.04	-2.38	-1.54	-2.12	-1.98	-0.89	-1.16	-3.56	-1.97	-1.85	0.36878711
Alanine	-2.37	-2.29	-2.94	-2.24	-3.16	-2.62	-2.84	-3.08	-1.69	-2.37	-2.20	-2.72	-2.28	-2.63	-2.29	-1.48	-1.53	-2.28	-2.51	-3.01	-3.94	-2.82	-2.72	-1.92	-2.90	-2.51	-2.30	-2.35	-2.46	-2.06	-2.53	-1.79	-2.43	-2.52	-2.33	0.37888270
Acetic Acid	-1.04	-1.19	-1.87	-1.37	-1.93	-1.46	-0.61	-2.14	-0.98	-1.75	-1.36	-2.42	-1.49	-1.60	-1.75	-0.77	-1.17	-1.51	-2.16	-1.74	-2.79	-1.06	-0.44	-2.06	-1.96	-0.75	-0.87	-0.65	-0.48	-1.24	-0.34	-0.82	-2.06	-0.85	-1.50	0.37898271
Leu-Glu	-0.16	-1.32	-1.67	1.60	-3.21	-0.99	-1.23	-1.70	-0.44	-1.27	-1.27	-1.90	-1.01	-1.52	-1.68	-0.81	-1.31	-0.63	-2.28	-2.40	-3.46	-1.38	-0.72	-1.16	-2.29	-0.51	-0.71	-0.83	-0.59	-1.55	-0.91	-0.76	-1.59	-0.19	-2.83	0.38247205
Pro-Ala	-2.94	-1.85	-2.90	-2.18	-3.08	-3.00	-2.80	-3.38	-1.49	-1.94	-1.94	-2.47	-2.32	-2.01	-2.46	-1.72	-1.89	-2.31	-3.02	-2.89	-3.51	-2.78	-2.71	-1.63	-2.58	-1.92	-2.29	-1.75	-3.66	-2.36	-1.95	-1.82	-3.38	-2.37	-2.63	0.38725938
Acetoacetic Acid	-0.91	-1.61	-2.24	1.19	-2.09	-1.83	-1.51	-1.77	-1.67	-2.41	-1.66	-2.75	-1.33	-1.35	-1.52	-0.76	-1.05	-0.96	-1.77	-1.55	-2.58	-1.08	-0.81	-1.18	-1.80	-1.03	-0.81	-0.78	-0.34	-1.44	-0.43	-1.89	-1.63	-0.21	-1.70	0.38955644
Fucose	-1.10	-1.81	-2.24	-1.78	-2.87	-2.63	-0.66	-2.05	-1.56	-2.27	-2.00	-2.63	-1.90	-1.65	-1.78	-1.25	-1.53	-2.08	-3.12	-3.02	-3.49	-2.19	-1.24	-1.27	-2.37	-2.11	-1.94	-1.62	-2.22	-2.15	-0.95	-1.30	-2.03	-1.77	-2.27	0.39195218
Xylitol	-0.72	-1.23	-1.83	-0.27	-1.87	-2.34	-0.06	-1.02	-0.81	-1.45	-0.75	-1.80	-0.79	-0.70	-1.29	-0.73	-0.77	-1.15	-3.20	-1.84	-3.20	-2.68	-1.63	-0.69	-1.56	-0.32	-0.78	-0.75	-0.54	-1.21	-0.51	0.44	-2.24	-0.72	-1.23	0.39928321
Val-Glu	-1.63	-1.55	-3.18	-2.61	-3.47	-2.45	-2.58	-3.32	-1.02	-2.83	-2.83	-1.90	-2.59	-2.40	-2.47	-2.64	-2.22	-2.92	-3.76	-3.59	-3.78	-3.25	-2.90	-2.12	-2.73	-2.57	-1.99	-1.40	-3.47	-1.79	-1.40	-1.17	-3.56	-3.18	-3.24	0.40535094
Gln-Glu	-0.60	-0.51	-0.99	-0.08	-3.16	-2.66	-2.12	-3.18	-1.20	-2.99	-3.06	-3.20	-2.15	-2.20	-2.40	-1.50	-2.40	-2.31	-1.79	-2.45	-3.80	-1.19	-0.98	-1.14	-0.92	-1.49	-2.74	-0.46	-0.91	-0.95	-0.92	-2.80	-2.70	-2.20	-2.56	0.40890074
Lys-Glu	-2.92	-1.53	-2.91	-1.35	-2.75	-2.60	-2.56	-3.43	-0.88	-1.79	-1.79	-1.83	-2.05	-2.37	-2.61	-1.66	-1.64	-2.69	-3.46	-3.25	-3.32	-3.07	-2.85	-1.95	-3.08	-1.93	-2.02	-1.68	-2.92	-2.06	-1.36	-0.88	-2.18	-1.66	-2.99	0.40908960
Glu-Ala	-1.85	-1.92	-2.58	-2.65	-3.52	-2.57	-2.78	-2.74	-1.32	-1.87	-1.98	-2.13	-2.27	-2.25	-2.31	-1.91	-1.64	-2.47	-3.29	-3.13	-3.63	-3.07	-2.88	-2.22	-2.82	-1.79	-2.05	-1.93	-2.07	-1.47	-1.80	-1.36	-3.21	-2.10	-2.60	0.41031796
MethyGlucoside	-2.54	-1.96	-2.72	-2.58	-3.38	-2.86	-1.94	-2.97	-1.37	-2.24	-1.87	-2.53	-1.97	-1.91	-2.24	-1.51	-1.32	-2.54	-3.41	-3.20	-3.78	-3.02	-2.81	-1.03	-2.86	-1.99	-2.72	-1.99	-2.91	-2.10	-1.07	-0.95	-2.97	-2.64	-2.19	0.41292541
Pro-Asn	-2.83	-1.83	-2.76	-1.83	-3.66	-2.83	-2.77	-3.44	-1.19	-1.84	-1.84	-2.35	-1.76	-1.98	-2.32	-1.79	-1.65	-2.42	-3.61	-3.24	-3.78	-3.01	-2.94	-1.80	-2.62	-1.95	-2.17	-1.56	-2.55	-2.10	-1.67	-1.30	-2.88	-2.02	-2.89	0.41687421
Ser-Glu	-2.68	-1.62	-2.47	-2.01	-3.37	-2.70	-2.60	-3.51	-1.11	-1.81	-1.81	-2.24	-2.04	-2.34	-2.16	-2.10	-1.98	-2.45	-3.51	-3.27	-3.74	-3.43	-3.31	-1.81	-2.85	-1.61	-2.33	-1.88	-2.38	-1.81	-1.55	-1.07	-2.29	-2.06	-3.17	0.41879058
Met-Gly	-2.76	-2.19	-2.95	-2.37	-3.52	-2.59	-2.81	-2.91	-1.71	-2.24	-2.24	-2.85	-2.39	-2.36	-2.39	-1.51	-1.67	-1.97	-3.22	-3.76	-3.88	-3.15	-3.20	-1.82	-2.88	-2.30	-1.98	-1.69	-2.63	-1.79	-2.03	-1.46	-3.17	-2.40	-2.64	0.42188403
Pro-Asp	-2.85	-1.98	-2.79	-1.86	-3.41	-2.58	-2.58	-3.21	-1.24	-1.78	-1.78	-2.22	-1.74	-2.51	-2.31	-1.90	-1.83	-2.43	-3.51	-3.35	-3.80	-3.00	-2.92	-1.44	-2.97	-1.60	-2.64	-1.21	-2.37	-2.28	-1.44	-1.35	-2.97	-2.34	-2.83	0.42192299
Pro-Gln	-0.14	-0.63	-0.37	0.31	-1.97	-1.14	-0.59	-0.99	0.09	-0.21	-0.21	-0.88	0.07	-0.27	-0.85	-0.35	-1.17	-1.05	-1.77	-0.57	-2.72	-1.14	-0.47	-0.86	-0.62	0.21	-0.40	-0.85	-0.63	-0.30	-0.40	-0.36	-0.78	-0.11	-0.91	0.42313285
Leu-Gly	-0.72	-2.28	-2.68	-1.83	-2.89	-1.81	-2.08	-2.40	-1.64	-2.25	-2.25	-3.05	-2.31	-2.32	-1.93	-1.04	-1.52	-1.06	-2.95	-2.80	-3.49	-2.45	-1.81	-1.60	-2.92	-2.16	-1.35	-2.11	-1.77	-2.18	-1.90	-1.87	-2.21	-1.07	-2.32	0.42426667

Gln-Gly	-0.65	-0.59	-1.18	-1.07	-3.16	-2.21	-1.96	-3.59	-2.66	-2.74	-3.01	-3.28	-2.53	-2.15	-2.13	-2.21	-2.46	-2.71	-2.29	-2.06	-3.74	-1.70	-1.75	-1.28	-0.75	-1.86	-3.22	-0.57	-1.22	-0.94	-1.20	-2.88	-2.90	-2.46	-3.02	0.42553893
Thr-Met	-3.07	-2.18	-2.91	-2.18	-3.24	-2.54	-2.63	-3.27	-1.66	-2.14	-2.14	-3.06	-2.28	-2.23	-1.91	-1.37	-1.92	-1.99	-2.87	-3.51	-3.70	-3.07	-3.05	-2.06	-2.63	-2.14	-1.88	-1.59	-2.84	-1.92	-1.90	-1.50	-3.25	-2.43	-2.84	0.43079478
Glucose-1-P	-3.49	-2.01	-1.58	-0.17	-3.31	-1.74	-0.81	-1.98	-0.80	-1.65	-1.50	-1.72	-0.50	-0.99	-1.14	-0.72	-0.67	-1.18	-2.47	-2.32	-3.44	-2.25	-1.20	-0.70	-2.32	-0.96	-1.36	-1.33	-1.14	-1.36	-1.47	-1.02	-1.82	-1.26	-1.74	0.43193394
Lactulose	-1.25	-1.78	-2.83	-2.36	-3.47	-2.88	-1.60	-2.59	-1.35	-2.29	-1.92	-2.24	-2.06	-2.02	-2.15	-1.67	-1.49	-2.46	-3.46	-3.38	-3.68	-2.30	-1.55	-1.84	-2.81	-1.91	-2.36	-2.07	-2.61	-2.01	-1.04	-1.13	-2.68	-2.46	-2.05	0.43505513
Leu-Pro	-1.12	-2.05	-2.24	-1.61	-2.02	-2.04	-1.72	-2.25	-1.33	-1.71	-1.71	-2.73	-1.97	-2.05	-1.72	-0.91	-1.19	-1.18	-2.79	-2.81	-3.80	-2.19	-1.84	-1.16	-2.86	-1.58	-1.21	-1.79	-0.89	-1.61	-1.10	-1.42	-2.29	-1.22	-2.22	0.43668022
Ser-Met	-3.38	-2.06	-2.89	-2.06	-3.01	-2.70	-3.02	-2.93	-1.87	-2.34	-2.34	-2.91	-2.47	-2.43	-1.95	-1.62	-1.94	-1.98	-2.94	-3.51	-3.64	-3.27	-3.17	-2.38	-2.77	-2.04	-1.79	-1.72	-2.65	-2.08	-2.15	-1.81	-3.21	-2.24	-2.66	0.43740592
Sorbose	-1.14	-1.97	-2.54	-3.57	-3.22	-2.63	-1.00	-2.33	-1.37	-2.31	-1.84	-2.58	-1.86	-1.85	-1.74	-1.37	-1.47	-2.16	-3.49	-3.24	-3.29	-2.71	-1.56	-1.40	-2.40	-2.18	-2.54	-1.22	-2.47	-2.22	-1.33	-1.29	-2.06	-2.26	-2.40	0.43788361
Rhamnose	-1.28	-1.89	-2.64	-2.10	-3.31	-2.47	-1.65	-2.40	-1.37	-2.05	-1.86	-2.43	-1.84	-1.89	-1.80	-1.40	-1.46	-2.40	-3.54	-2.83	-3.51	-2.46	-1.49	-0.97	-2.79	-1.87	-2.22	-1.72	-2.53	-2.13	-1.37	-1.29	-1.71	-2.44	-2.24	0.43975587
Ile-Phe	-3.22	-2.12	-2.72	-2.22	-3.32	-2.82	-2.81	-3.80	-1.89	-2.27	-2.27	-3.05	-2.75	-2.75	-2.65	-2.18	-2.30	-2.69	-3.49	-3.51	-3.63	-3.17	-3.43	-2.24	-3.40	-2.88	-2.43	-2.24	-2.93	-2.31	-2.10	-1.48	-2.10	-3.18	-3.21	0.44320487
Pro-Ile	-3.12	-1.96	-2.81	-1.87	-3.64	-2.54	-2.47	-3.37	-1.22	-2.02	-2.02	-3.74	-2.36	-2.34	-2.60	-2.00	-1.81	-2.59	-3.92	-3.54	-4.04	-3.44	-3.08	-1.95	-2.46	-2.40	-3.11	-1.53	-3.17	-1.81	-2.13	-1.06	-2.13	-2.41	-3.25	0.44561849
Ala-Met	-2.31	-2.29	-3.07	-2.46	-2.82	-2.51	-2.70	-3.18	-1.95	-2.50	-2.42	-3.24	-2.40	-2.55	-2.18	-1.30	-1.41	-2.02	-2.70	-3.05	-3.78	-2.56	-2.66	-2.24	-2.97	-2.63	-1.99	-1.97	-2.87	-1.96	-1.56	-1.86	-3.44	-2.76	-2.37	0.44716849
Glucuronic Acid	0.52	-1.92	-2.71	-2.18	-3.22	-2.45	-2.31	-2.61	-1.57	-2.41	-2.03	-2.56	-2.02	-2.04	-2.51	-1.65	-1.43	-2.27	-3.31	-3.06	-3.40	-3.00	-2.40	-1.14	-2.49	-2.24	-2.68	-1.50	-2.43	-2.33	-1.01	-1.44	-1.96	-1.89	-2.32	0.44755970
Asp-Asp	-3.06	-1.95	-2.86	-2.47	-3.61	-2.85	-2.84	-2.85	-1.58	-2.10	-1.84	-2.45	-1.97	-2.20	-2.26	-1.38	-1.25	-2.02	-2.78	-2.92	-4.01	-2.27	-1.94	-2.44	-2.27	-2.46	-2.17	-1.79	-2.71	-2.13	-1.60	-1.46	-3.88	-2.98	-2.31	0.45413710
MethyXyloside	-1.85	-1.98	-2.77	-0.61	-3.35	-2.64	-1.48	-2.66	-1.51	-2.23	-1.94	-2.57	-1.88	-1.93	-2.27	-1.53	-1.57	-2.13	-3.24	-3.24	-3.57	-2.82	-2.65	-1.33	-2.85	-2.23	-2.73	-1.37	-2.18	-2.22	-1.23	0.07	-2.09	-2.25	-2.29	0.45588007
Fucose	-0.61	-1.87	-2.77	-1.93	-3.27	-2.47	-0.87	-2.33	-1.16	-2.20	-1.73	-2.46	-1.99	-2.01	-1.69	-1.35	-1.53	-2.27	-3.21	-3.13	-3.46	-2.56	-1.37	-0.95	-2.57	-1.63	-2.29	-1.86	-2.33	-1.89	-0.97	-1.06	-2.13	-2.13	-2.15	0.45608190
Succinic Acid	-2.93	-1.30	-1.96	-1.63	-3.06	-2.62	-0.94	-2.27	-0.60	-1.68	-2.51	-1.66	-1.69	-1.55	-2.34	-1.63	-1.45	-2.25	-2.98	-2.74	-3.05	-1.95	-2.05	-1.01	-2.13	-1.11	-2.56	-0.61	-0.51	-1.65	-0.75	-0.13	-2.26	-1.06	-2.16	0.45705584
Malic Acid	-0.86	-1.86	-2.71	-2.53	-3.22	-3.29	-2.72	-3.05	-1.36	-2.22	-2.17	-2.34	-2.03	-2.29	-2.36	-1.42	-1.28	-2.51	-3.31	-2.51	-3.38	-1.99	-1.95	-1.82	-2.89	-1.63	-2.65	-1.13	-1.54	-1.94	-0.94	-0.77	-2.63	-1.63	-2.14	0.45720935
Ile-Ile	-3.01	-4.92	-2.89	-2.63	-2.92	-2.51	-2.38	-3.59	-1.10	-1.61	-1.61	-2.10	-1.87	-2.37	-2.55	-1.45	-1.29	-2.11	-3.00	-3.64	-3.80	-2.70	-2.50	-2.63	-2.31	-2.17	-2.37	-2.15	-3.40	-1.83	-1.50	-1.14	-3.08	-3.41	-2.38	0.47039647
Pro-Pro	-3.17	-1.82	-2.85	-2.44	-3.41	-2.55	-2.81	-3.34	-1.25	-1.86	-1.86	-2.34	-2.12	-2.14	-2.04	-1.67	-1.85	-1.93	-3.41	-3.12	-3.56	-2.74	-2.65	-1.79	-2.15	-1.97	-1.95	-1.80	-2.56	-2.28	-1.55	-1.53	-3.17	-2.66	-2.87	0.47301511
Methyl Glucose	-1.97	-1.61	-2.47	-1.79	-2.97	-3.06	-1.78	-3.13	-1.10	-1.86	-1.49	-2.10	-1.71	-1.70	-1.95	-1.17	-1.22	-1.89	-3.28	-3.05	-3.12	-2.52	-2.48	-0.95	-2.24	-1.53	-2.40	-1.64	-1.65	-1.87	-0.83	-1.06	-2.90	-2.50	-1.91	0.47324557
Pro-Leu	-1.49	-2.09	-2.30	-1.61	-2.24	-1.79	-1.86	-2.43	-1.21	-2.03	-2.03	-2.49	-1.87	-1.83	-1.76	-0.92	-1.28	-1.09	-2.77	-2.52	-3.47	-2.37	-1.64	-1.56	-2.78	-1.47	-1.06	-1.71	-1.07	-1.67	-1.60	-1.63	-2.10	-1.25	-2.24	0.47732452
Pyruvic Acid	-0.12	-1.55	-0.93	0.09	-1.29	-0.38	-0.16	-1.06	-0.23	-1.42	-1.05	-1.55	-0.82	-0.69	-1.18	-1.28	-0.54	-0.45	-1.27	-0.63	-0.45	-1.00	-1.04	-1.11	-2.19	-0.43	-0.78	-0.83	-0.63	-2.15	-0.75	-0.23	-1.35	-0.70	-0.47	0.47997517
Arg-Gln	-0.86	-0.28	-0.51	0.05	-1.89	-1.40	-1.31	-0.90	-0.12	-1.08	-1.01	-1.21	-0.29	-0.54	-0.53	-0.34	-0.66	-1.07	-1.91	-0.43	-3.21	-0.95	-0.38	-1.19	-0.60	-0.33	-0.57	-0.61	-0.54	-0.13	-0.36	-1.08	-2.01	-0.46	-1.16	0.48153988
Phe-Asp	-3.28	-2.14	-3.15	-2.86	-3.24	-3.06	-2.79	-3.56	-2.23	-2.51	-2.51	-3.01	-3.02	-3.04	-3.29	-1.92	-2.03	-2.93	-3.56	-3.78	-3.78	-3.54	-3.16	-2.22	-2.93	-3.11	-2.20	-2.31	-3.78	-2.06	-2.08	-1.56	-4.21	-2.73	-3.36	0.49054228
Thr-Ser	-3.24	-1.81	-2.13	-1.35	-2.98	-2.59	-2.65	-2.55	-1.46	-1.73	-1.73	-2.69	-1.94	-1.86	-1.75	-1.31	-1.43	-1.74	-2.83	-3.37	-3.56	-3.12	-2.95	-1.40	-2.66	-1.77	-1.61	-1.15	-1.47	-1.86	-1.35	-1.41	-2.89	-1.93	-2.36	0.49235607
Raffinose	-2.04	-1.82	-2.59	-2.20	-3.20	-2.53	-0.45	-2.40	-1.24	-2.04	-1.64	-2.40	-1.95	-1.72	-2.03	-1.29	-1.36	-2.29	-3.22	-3.40	-3.35	-2.34	-1.52	-1.39	-2.75	-1.99	-2.31	-1.79	-2.32	-1.77	-1.02	-0.76	-1.76	-2.27	-2.05	0.49390805
Met-Asp	-3.28	-2.08	-2.99	-2.07	-3.27	-2.60	-2.89	-3.34	-1.40	-2.22	-2.22	-2.72	-2.36	-2.36	-2.22	-1.72	-1.87	-2.55	-3.27	-3.40	-3.86	-3.17	-3.12	-1.92	-2.92	-2.37	-2.18	-1.29	-2.90	-2.07	-1.45	-1.45	-3.92	-2.16	-3.07	0.49456436
Leu-Asn	-1.53	-1.99	-2.34	-1.43	-2.82	-1.21	-1.54	-2.28	-1.16	-1.97	-1.97	-2.72	-2.05	-1.95	-1.52	-0.87	-1.14	-1.04	-2.44	-2.56	-3.78	-2.05	-1.54	-1.81	-2.74	-1.93	-1.16	-1.55	-1.07	-1.67	-1.19	-1.41	-2.10	-1.18	-2.15	0.49461700
Met-Arg	-1.74	-1.80	-2.72	-1.59	-3.34	-2.11	-2.01	-2.74	-1.76	-2.14	-2.14	-3.16	-2.51	-2.44	-2.04	-1.03	-1.77	-1.45	-2.59	-2.64	-3.49	-1.89	-1.62	-1.14	-2.64	-2.23	-1.57	-1.18	-1.62	-1.78	-1.13	-1.62	-2.70	-1.64	-2.37	0.49521873
Glucose	0.49	0.17	-0.13	0.72	0.28	0.80	0.82	0.20	0.25	-0.51	0.08	-0.95	0.32	0.41	0.51	0.85	0.84	0.94	0.31	0.97	0.78	0.38	0.91	0.52	-0.45	0.58	-0.05	-0.55	0.35	0.04	0.21	0.39	-2.30	0.27	0.83	0.50106448
Ala-Ala	-2.74	-2.15	-3.00	-2.26	-2.92	-2.40	-4.06	-3.02	-1.58	-2.36	-2.24	-2.83	-2.33	-2.30	-2.32	-1.41	-1.29	-1.89	-2.38	-2.90	-5.01	-2.71	-2.21	-2.62	-2.92	-2.48	-2.27	-2.13	-2.31	-2.18	-1.45	-1.83	-3.32	-2.56	-2.22	0.50150989
Glutamic Acid	-2.88	-1.25	-2.77	-2.06	-3.41	-2.60	-2.65	-3.37	-1.98	-2.08	-3.13	-2.13	-2.32	-2.47	-2.50	-2.45	-2.51	-2.82	-3.20	-2.93	-3.46	-3.02	-2.89	-1.63	-2.89	-1.86	-3.05	-1.20	-1.97	-2.53	-1.43	-1.23	-1.95	-2.27	-2.86	0.50456088
Pro-Glu	-3.04	-1.78	-2.52	-1.79	-3.54	-2.58	-2.47	-3.34	-0.99	-2.02	-2.02	-1.87	-2.50	-2.48	-2.98	-2.23	-2.47	-2.65	-3.56	-3.12	-3.88	-3.05	-2.95	-1.63	-2.88	-1.84	-2.94	-1.36	-3.12	-1.93	-1.67	-0.99	-3.61	-2.15	-3.12	0.50485069
Arg-Met	-2.00	-2.08	-2.59	-1.82	-3.47	-2.71	-2.66	-3.01	-1.82	-2.68	-2.42	-2.95	-2.40	-2.39	-2.22	-1.66	-1.58	-2.20	-3.15	-3.38	-3.76	-3.17	-3.01	-1.60	-2.84	-2.22	-2.02	-1.67	-2.16	-2.65	-1.71	-1.63	-2.63	-2.16	-2.74	0.52213618
Gentiobiose	-1.86	-1.80	-2.84	-3.04	-3.08	-2.67	-2.09	-3.10	-1.34	-2.10	-1.93	-2.47	-1.63	-2.08	-1.92	-1.36	-1.59	-2.37	-3.08	-2.78	-3.57	-1.99	-1.46	-2.40	-2.20	-2.60	-2.31	-1.16	-2.93	-2.06	-1.07	-1.29	-3.90	-2.98	-1.92	0.52885605
Ala-Leu	-1.48	-2.04	-2.56	1.58	-2.31	-1.83	-1.84	-2.51	-1.54	-2.59	-2.15	-2.79	-2.18	-2.18	-1.82	-0.87	-1.15	-1.01	-2.51	-2.01	-2.79	-2.16	-1.60	-1.45	-2.71	-1.96	-1.03	-1.33	-1.10	-1.76	-1.81	-2.01	-2.47	-1.39	-2.26	0.54280687
Isoleucine	-2.73	-1.97	-2.76	-2.81	-3.37	-2.46	-2.49	-3.61	-1.30	-2.05	-1.95	-2.29	-2.03	-2.12	-2.87	-1.59	-1.35	-2.70	-3.31	-3.24	-3.72	-3.06	-2.97	-2.17	-1.84	-2.25	-2.40	-1.68	-3.15	-1.82	-1.75	-1.05	-2.31	-2.98	-2.79	0.54509329
Leu-Arg	-1.20	-1.98	-2.41	-1.83	-2.97	-1.39	-1.42	-2.16	-1.58	-2.11	-2.11	-2.97	-2.42	-2.27	-1.85	-0.74	-1.30	-0.89	-2.47	-1.89	-2.77	-1.90	-1.07	-1.50	-2.71	-1.70	-1.06	-1.64	-1.27	-1.54	-1.29	-1.60	-2.28	-1.03	-2.01	0.56296119
Asp-Glu	-2.63	-1.44	-2.61	-2.56	-3.13	-2.87	-2.95	-2.93	-1.19	-1.70	-2.58	-1.56	-2.43	-2.40	-2.87	-1.97	-2.29	-2.68	-2.88	-2.77	-3.44	-2.58	-2.49	-1.25	-2.27	-2.49	-3.16	-1.14	-1.86	-1.09	-1.62	-0.92	-3.29	-1.74	-3.08	0.57443617
Glucose	-2.79	-2.08	-2.84	-2.20	-3.37	-2.73	-2.37	-3.10	-1.56	-2.21	-2.00	-2.62	-1.94	-2.12	-2.05	-1.57	-1.43	-2.35	-3.54	-3.15	-3.29	-2.93	-2.90	-0.92	-2.37	-2.22	-2.72	-1.65	-3.04	-2.32	-2.01	-1.10	-2.70	-1.87	-2.40	0.57692714
Val-Arg	-1.16	-1.77	-2.06	-1.31	-3.41	-1.32	-1.22	-2.15	-1.34	-1.79	-1.79	-2.80	-1.69	-2.11	-1.80	-1.00	-1.37	-1.25	-2.84	-2.59	-3.59	-1.83	-1.07	-1.39	-2.51	-1.25	-1.58	-1.03	-2.80	-1.63	-0.69	-1.34	-2.40	-1.15	-2.23	0.57852265
Met-Pro	-3.47	-1.83	-2.74	-2.23	-3.38	-2.58	-2.61	-3.72	-1.53	-2.23	-2.23	-2.59	-2.08	-2.63	-2.72	-2.00	-1.89	-2.56	-3.41	-3.46	-3.59	-3.28	-2.95	-1.69	-2.52	-2.47	-2.98	-1.51	-2.78	-2.30	-1.38	-1.31	-3.56	-2.51	-3.01	0.58247781
Met-Leu	-1.81	-1.96	-2.84	-1.98	-3.54	-2.04	-2.01	-2.90	-1.76	-2.06	-2.06	-2.93	-2.42	-2.58	-2.10	-0.95	-1.27	-1.29	-2.54	-2.60	-3.63	-2.06	-1.58	-1.77	-2.71	-1.97	-1.59	-1.54	-1.47	-1.73	-1.29	-1.72	-2.76	-1.23	-2.14	0.58726660
Glycerol	-2.56	-2.12	-2.76	-1.67	-3.32	-2.73	-1.91	-3.11	-1.30	-2.22	-1.78	-2.31	-1.76	-2.06	-1.94	-1.42	-1.33	-2.45	-3.31	-3.21	-3.82	-2.52	-2.37	-1.75	-2.95	-1.71	-2.18	-2.14	-2.80	-1.91	-1.18	0.28	-3.08	-2.42	-2.03	0.58732253
Thr-Glu	-2.86	-1.69	-2.90	-2.42	-3.24	-2.80	-2.79	-3.63	-0.98	-1.89	-1.89	-1.89	-2.74	-2.48	-2.69	-1.77	-2.12	-2.62	-3.59	-3.47	-3.64	-3.35	-2.86	-2.00	-2.90	-2.10	-2.18	-1.35	-3.13	-1.63	-1.22	-0.97	-3.38	-2.34	-3.34	0.58905799
Methionine	-3.07	-2.01	-2.95	-2.71	-3.21	-2.63	-2.91	-3.66	-2.05	-2.25	-2.31	-2.67	-2.53	-2.55	-2.21	-1.75	-1.69	-2.24	-2.51	-2.99	-3.52	-2.90	-2.70	-1.53	-2.57	-2.99	-2.79	-2.12	-2.87	-2.22	-1.62	-1.57	-3.47	-3.47	-2.80	0.60135062
Arg-Glu	-2.47	-1.60	-2.58	-2.08	-3.63	-2.55	-2.66	-3.08	-1.10	-1.60	-1.86	-1.75	-1.94	-2.22	-2.76	-1.16	-1.20	-2.62	-2.91	-2.93	-4.08	-2.76	-2.55	-1.52	-2.54	-1.76	-2.24	-1.51	-2.24	-1.65	-1.29	-0.88	-2.86	-2.48	-2.74	0.60327784
Butanone	-2.52	-1.89	-2.51	-2.40	-3.01	-2.37	-1.62	-2.37	-1.40	-2.08	-1.93	-2.51	-1.99	-1.74	-2.16	-1.29	-1.41	-2.34	-3.11	-0.64	-3.74	-2.04	-1.85	-1.72	-2.30	-2.15	-2.41	-1.21	-1.74	-1.92	-1.14	-1.09	-2.15	-2.34	-2.06	0.61239943
Glucosaminic Acid	0.57	-1.72	-2.54	-2.24	-3.20	-2.74	-2.23	-3.00	-1.86	-2.25	-2.14	-2.47	-2.08	-1.91	-2.25	-1.48	-1.43	-2.31	-3.32	-2.90	-3.56	-2.61	-2.35	-1.19	-2.51	-2.32	-2.67	-1.30	-2.01	-2.28	-1.24	-1.38	-1.99	-1.42	-2.40	0.61290413
Met-Lys	-3.31	-1.64	-2.74	-2.48	-3.16	-2.42	-2.63	-3.12	-1.80	-2.10	-2.10	-2.76	-2.27	-2.35	-2.28	-1.63	-1.66	-2.26	-3.08	-2.85	-3.63	-2.75	-2.51	-1.85	-2.34	-2.37	-2.15	-1.74	-3.29	-2.15	-1.45	-1.47	-3.51	-2.34	-2.60	0.61297486
Tartaric Acid	-0.73	-1.81	-2.66	1.16	-3.28	-2.82	-2.84	-2.85	-1.64	-2.32	-2.07	-2.69	-2.31	-2.50	-2.62	-1.45	-1.43	-2.25	-3.02	-2.79	-3.41	-2.08	-1.85	-1.92	-2.62	-2.10	-2.50	-1.02	-2.87	-2.25	-1.00	-1.14	-3.02	-1.78	-2.24	0.61647785
Amino Butyric Acid	-3.10	-1.39	-2.45	-1.91	-3.27	-2.76	-2.10	-3.47	-1.42	-2.11	-1.79	-2.34	-1.79	-1.80	-2.24	-1.43	-1.62	-2.54	-3.11	-1.70	-3.80	-2.60	-4.32	-1.30	-2.38	-2.15	-2.90	-1.42	-2.86	-2.18	-1.08	-1.03	-1.82	-2.49	-2.42	0.62772673
Ser-His	-1.65	-1.62	-2.38	-1.60	-3.37	-2.10	-2.48	-2.63	-1.74	-2.18	-2.18	-2.90	-2.37	-2.16	-1.78	-1.18	-1.26	-1.44	-2.66	-2.49	-3.51	-2.31	-1.46	-2.11	-2.61	-1.67	-2.15	-1.45	-3.00	-2.17	-1.42	-1.72	-2.10	-1.68	-2.08	0.62799285
acetylneuraminic acid	-1.93	-1.84	-2.35	-1.61	-3.07	-2.51	-1.22	-2.30	-1.22	-2.30	-1.71	-2.24	-1.76	-1.86	-1.71	-1.31	-1.41	-2.14	-3.28	-3.04	-3.59	-2.85	-2.41	-1.16	-1.98	-1.53	-2.06	-1.39	-1.33	-1.76	-1.30	-1.04	-1.60	-1.82	-2.27	0.62898976
Tricarballylic Acid	-1.21	-1.93	-2.79	-2.72	-3.38	-2.77	-2.84	-3.40	-1.49	-2.32	-1.81	-2.53	-2.28	-2.33	-2.46	-1.77	-1.49	-2.70	-3.43	-2.82	-3.41	-2.58	-2.17	-1.04	-2.51	-2.31	-3.21	-1.36	-2.10	-2.47	-1.32	-1.28	-2.01	-1.46	-2.49	0.63625276
Glucose	0.37	0.37	-0.04	0.59	0.03	0.88	0.70	-0.57	0.16	-0.03	-0.03	-0.41	0.44	0.39	0.41	0.75	0.65	0.74	0.39	0.61	0.43	0.73	1.01	-0.24	-0.03	0.79	-0.19	-0.08	0.26	0.23	0.12	-0.04	-0.52	-0.01	0.54	0.64494866
Leu-His	-0.64	-2.06	-2.46	-1.70	-2.74	-1.64	-1.83	-2.53	-1.82	-2.20	-2.20	-3.37	-2.48	-2.46	-2.06	-0.96	-1.50	-1.10	-2.74	-2.78	-3.37	-2.47	-1.86	-1.67	-3.00	-1.62	-1.33	-2.04	-1.33	-2.01	-1.53	-1.96	-2.31	-1.14	-2.34	0.64754060
Thr-Leu	-1.64	-2.14	-2.77	-1.77	-2.22	-1.49	-1.87	-2.52	-1.62	-2.13	-2.13	-2.89	-2.23	-2.17	-1.81	-0.93	-1.53	-1.11	-2.76	-2.67	-3.57	-2.30	-1.62	-1.26	-2.79	-1.77	-1.37	-1.62	-1.23	-1.87	-1.84	-1.78	-2.40	-1.28	-2.47	0.65057407
Melibionic Acid	-2.55	-1.60	-2.50	-3.11	-3.08	-3.10	-2.56	-3.56	-1.47	-2.32	-1.89	-2.38	-2.12	-2.31	-2.37	-1.57	-1.67	-2.28	-3.41	-2.90	-3.64	-2.68	-2.51	-1.36	-2.44	-2.56	-2.52	-0.94	-2.47	-2.45	-1.23	-1.46	-1.76	-1.57	-2.42	0.65571575
Thr-Gly	-2.84	-2.01	-2.84	-2.57	-3.38	-2.65	-2.71	-3.22	-1.65	-2.01	-2.01	-2.70	-2.30	-2.27	-1.91	-1.48	-1.71	-1.93	-3.08	-3.56	-3.68	-2.84	-2.72	-0.98	-2.76	-2.01	-1.94	-1.58	-2.18	-2.27	-1.69	-1.60	-3.24	-2.61	-2.80	0.65821208
Pro-Lys	-1.61	-1.81	-2.11	-1.44	-3.59	-1.66	-1.74	-2.27	-1.21	-1.69	-1.69	-2.37	-1.69	-1.70	-2.02	-0.91	-1.14	-1.33	-2.38	-2.58	-3.66	-1.93	-1.20	-1.22	-2.24	-1.01	-1.99	-0.92	-2.94	-1.64	-1.04	-1.43	-2.47	-1.22	-2.07	0.66454050
Lactitol	-2.60	-1.83	-2.56	-2.47	-3.47	-2.73	-1.80	-2.85	-1.37	-2.32	-1.96	-2.42	-1.96	-1.85	-2.18	-1.40	-1.41	-2.48	-3.49	-3.56	-3.47	-2.51	-1.86	-1.64	-2.62	-1.82	-2.51	-2.20	-2.35	-1.79	-1.04	-0.97	-2.86	-2.22	-2.22	0.66864247
Pro-Phe	-3.06	-1.95	-2.80	-3.49	-3.64	-2.90	-3.97	-3.82	-1.82	-2.25	-2.25	-2.67	-2.73	-2.86	-2.43	-1.76	-1.70	-2.25	-2.93	-3.32	-3.70	-3.31	-2.83	-1.93	-2.88	-2.68	-2.29	-2.94	-3.31	-1.91	-1.70	-1.61	-3.59	-3.56	-2.84	0.67740701
Leu-Ala	-1.37	-1.99	-2.65	-1.70	-2.31	-1.56	-1.70	-2.51	-1.67	-2.06	-2.06	-2.84	-2.10	-2.47	-1.90	-0.91	-1.36	-1.06	-2.63	-2.19	-3.52	-2.28	-1.60	-1.54	-2.83	-2.09	-1.18	-1.78	-1.37	-1.81	-1.42	-1.82	-2.43	-1.10	-2.11	0.68604709
Arg-Lys	-0.73	-1.78	-2.17	-1.55	-2.12	-1.56	-1.54	-2.14	-1.60	-2.54	-1.62	-2.67	-1.97	-1.94	-1.75	-0.99	-1.33	-1.34	-2.24	-2.40	-3.15	-1.80	-0.94	-1.26	-2.46	-1.42	-1.09	-0.73	-0.76	-2.38	-0.69	-1.69	-1.85	-1.21	-2.18	0.71018170
Lys-Leu	-0.82	-1.50	-2.29	-1.28	-2.20	-1.19	-1.44	-2.16	-1.45	-1.76	-1.76	-2.84	-1.93	-2.12	-1.48	-0.91	-1.24	-0.92	-2.28	-1.75	-2.44	-1.85	-0.89	-1.37	-2.82	-1.62	-0.81	-0.98	-1.00	-1.58	-0.99	-1.59	-1.82	-0.84	-1.83	0.71776446
Adonitol	-2.51	-1.42	-2.44	-1.43	-3.22	-2.63	-2.46	-2.95	-1.31	-2.16	-1.53	-2.46	-1.84	-1.71	-2.03	-1.30	-1.32	-2.00	-3.29	-2.91	-3.35	-2.70	-2.46	-1.04	-2.12	-1.99	-2.43	-1.04	-1.77	-1.92	-1.16	0.13	-2.13	-0.77	-2.16	0.73134150
Sedoheptulosan	-2.54	-1.62	-2.51	-1.99	-3.34	-2.61	-2.42	-2.36	-1.24	-2.03	-2.08	-2.21	-1.80	-2.06	-2.10	-1.45	-1.37	-2.13	-3.01	-2.91	-3.59	-2.68	-2.34	-1.30	-2.02	-1.70	-2.18	-1.51	-1.77	-1.74	-1.15	-0.77	-1.97	-1.81	-2.10	0.73873448
Gly-Arg	-1.61	-1.98	-2.47	-1.79	-2.33	-1.91	-1.84	-2.56	-1.80	-2.43	-2.09	-2.87	-2.28	-2.13	-1.66	-1.03	-1.25	-1.40	-2.59	-2.32	-3.80	-2.23	-1.47	-1.47	-2.73	-2.08	-1.74	-0.90	-1.45	-1.97	-1.11	-1.57	-3.01	-2.01	-2.17	0.74720615
Ser-Gln	-0.02	-0.30	-0.43	0.28	-3.37	-1.04	-0.66	-1.06	0.04	-0.66	-0.66	-1.33	-0.68	-0.43	-2.28	-1.03	-1.92	-1.05	-2.39	-0.78	-2.69	-1.63	-0.72	-1.54	-0.67	0.08	-1.55	-0.39	-1.04	-0.36	-0.40	-0.94	-0.77	-0.29	-1.12	0.74809976
Propanediol	-2.42	-1.88	-2.53	-1.71	-3.25	-3.06	-1.98	-2.75	-1.39	-2.13	-2.01	-2.38	-1.87	-1.96	-1.80	-1.41	-1.46	-2.29	-3.35	-3.34	-3.97	-2.68	-2.66	-1.02	-2.90	-1.70	-1.79	-1.64	-3.04	-1.98	-1.27	0.06	-1.78	-2.28	-2.28	0.75319827
Tagatose	-1.65	-1.61	-2.24	-2.80	-3.00	-2.56	-2.18	-3.10	-1.59	-2.30	-1.97	-2.38	-1.79	-1.93	-1.72	-1.45	-1.48	-2.00	-3.22	-2.91	-3.37	-2.37	-2.26	-1.09	-2.41	-2.18	-2.56	-0.99	-2.99	-2.01	-1.26	-1.29	-2.20	-1.30	-2.41	0.75569115
Arg-Leu	-1.70	-1.89	-2.55	1.33	-2.07	-1.43	-1.68	-2.42	-1.76	-2.47	-1.80	-2.81	-2.44	-2.18	-1.92	-0.84	-1.34	-0.97	-1.97	-1.49	-2.55	-1.47	-0.90	-1.28	-2.47	-1.62	-0.82	-1.32	-0.91	-2.31	-1.12	-1.82	-2.47	-1.31	-2.03	0.76078670
Leu-Asp	-1.43	-2.24	-2.57	-1.82	-3.47	-1.61	-1.66	-2.57	-1.36	-1.98	-1.98	-2.63	-2.00	-2.23	-1.94	-1.11	-1.29	-1.32	-2.59	-2.86	-4.44	-2.10	-1.58	-1.98	-2.76	-1.31	-1.48	-1.51	-1.01	-1.87	-1.72	-1.49	-2.25	-1.29	-2.25	0.76749425
Mannan	0.32	-1.94	-2.79	-2.04	-2.29	-2.13	-1.52	-2.44	-1.16	-2.09	-1.51	-2.37	-1.90	-2.02	-2.01	-1.37	-1.52	-1.43	-2.95	-2.13	-3.54	-1.40	-0.78	-2.39	-2.04	-1.61	-1.37	-1.24	-0.95	-1.67	-1.63	-0.90	-1.45	-1.16	-2.00	0.76783581
Glutaric Acid	-3.22	-1.33	-1.88	-1.04	-2.56	-2.56	-0.59	-1.29	-0.51	-1.37	-1.25	-1.63	-1.18	-0.73	-1.71	-0.97	-0.87	-1.54	-2.71	-2.15	-2.83	-1.67	-1.42	-0.81	-2.16	-0.88	-1.57	-0.65	-0.52	-1.40	-0.62	-0.11	-1.47	-1.04	-1.52	0.77380915
Glucose	0.32	0.28	0.04	0.99	0.37	0.90	0.75	0.00	0.24	-0.48	0.07	-0.70	0.30	0.40	0.44	0.82	0.99	1.06	0.44	1.09	0.94	0.32	0.89	0.70	-0.46	0.61	0.64	-0.09	0.44	0.09	0.29	0.52	-1.85	0.26	0.61	0.77785741
Aspartic Acid	-3.20	-1.68	-2.52	-2.32	-3.16	-2.65	-3.01	-3.68	-1.34	-1.92	-2.45	-2.14	-2.45	-2.61	-2.84	-2.08	-1.82	-2.67	-2.48	-2.74	-3.84	-2.90	-2.60	-1.83	-2.59	-2.79	-3.28	-1.09	-3.32	-2.58	-1.43	-1.19	-2.32	-3.17	-3.04	0.77793389
Glu-Asp	-0.82	-1.70	-2.57	-2.61	-3.59	-2.78	-2.66	-2.40	-0.94	-1.45	-1.69	-1.76	-2.34	-2.31	-2.50	-1.41	-1.28	-2.75	-3.29	-3.13	-3.90	-2.67	-2.72	-1.75	-2.27	-1.62	-2.37	-1.18	-1.73	-1.19	-1.33	-0.65	-2.27	-1.82	-2.87	0.78324849
Arabinose	-2.67	-1.86	-2.51	-1.36	-3.35	-2.82	-1.64	-2.49	-1.79	-2.21	-1.65	-2.46	-1.79	-1.80	-2.15	-1.44	-1.55	-2.02	-3.31	-3.10	-3.43	-3.02	-2.56	-1.02	-2.77	-1.89	-2.76	-1.59	-2.10	-2.10	-1.20	0.13	-1.77	-1.88	-2.30	0.78621269
Mannose	1.07	0.51	0.16	0.84	-0.13	1.01	0.97	0.00	0.20	-0.24	-0.11	-0.69	0.37	0.45	0.42	0.62	0.84	0.97	0.33	0.99	1.01	0.55	0.87	0.12	-0.08	0.73	0.55	-0.03	0.46	0.25	0.28	0.24	-1.17	0.26	0.70	0.78689619
Leu-Ser	-1.28	-2.00	-2.30	-1.71	-3.43	-1.79	-1.67	-2.26	-1.62	-2.09	-2.09	-2.92	-2.13	-1.88	-1.64	-0.93	-1.25	-1.10	-2.62	-2.35	-2.88	-2.09	-1.68	-2.00	-2.92	-2.09	-1.24	-1.84	-1.23	-1.69	-1.65	-1.64	-1.88	-1.17	-2.11	0.78981710
MethylGalactoside	-2.08	-1.85	-2.70	-1.88	-2.87	-2.62	-1.82	-2.66	-1.10	-2.43	-1.87	-2.40	-1.84	-1.85	-1.97	-1.40	-1.60	-2.27	-3.57	-2.95	-3.57	-3.21	-2.35	-1.53	-1.85	-1.71	-2.22	-1.61	-2.20	-1.90	-1.01	-0.93	-1.51	-1.86	-2.16	0.79500644
Lys-Tyr	-1.44	-1.61	-1.98	-1.48	-3.70	-1.79	-2.22	-1.86	-1.39	-1.75	-1.75	-2.25	-1.02	-1.14	-1.53	-0.85	-1.18	-1.17	-2.67	-2.40	-2.81	-1.86	-0.97	-1.06	-2.22	-2.02	-1.17	-0.78	-1.03	-1.88	-0.82	-1.74	-2.71	-1.18	-2.12	0.79678717
Butanediol	-2.88	-1.95	-2.65	-2.30	-3.47	-2.56	-1.25	-2.92	-1.38	-2.04	-1.92	-2.46	-2.17	-1.99	-2.19	-1.34	-1.47	-2.52	-3.56	-2.93	-3.57	-2.22	-1.84	-1.64	-2.69	-2.20	-2.47	-1.41	-2.65	-1.93	-1.11	-1.13	-2.59	-2.02	-2.28	0.80177796
Ala-Asn	-2.98	-2.05	-2.80	-2.13	-3.25	-2.61	-2.63	-2.98	-1.47	-2.31	-1.88	-2.38	-2.05	-2.20	-2.08	-1.70	-1.35	-2.10	-2.85	-3.41	-3.49	-3.10	-2.95	-1.80	-2.51	-2.20	-2.04	-1.43	-1.78	-1.98	-1.60	-1.67	-2.42	-2.02	-2.24	0.80997372
Arg-Ala	-1.73	-2.05	-2.47	-1.52	-2.32	-2.39	-1.96	-2.31	-1.84	-2.52	-1.93	-2.77	-2.36	-2.21	-1.69	-0.89	-1.47	-1.60	-2.68	-2.31	-3.41	-2.46	-1.48	-1.28	-2.48	-1.85	-1.66	-1.11	-1.18	-1.76	-1.08	-1.69	-3.07	-1.55	-2.16	0.81273162
Pro-Arg	-1.27	-1.63	-2.22	-1.41	-3.52	-1.89	-2.09	-2.40	-1.30	-1.59	-1.59	-2.77	-1.92	-2.07	-2.04	-1.02	-1.48	-1.31	-2.56	-2.59	-3.54	-1.73	-1.08	-0.92	-1.99	-1.48	-2.07	-0.77	-2.18	-1.85	-1.03	-1.51	-2.15	-1.00	-2.28	0.81318560
Leucine	-1.49	-2.15	-2.74	-1.98	-2.63	-1.67	-1.87	-2.63	-1.70	-2.47	-2.16	-2.79	-2.20	-2.37	-1.98	-0.91	-1.25	-1.22	-2.60	-2.22	-3.51	-2.39	-1.70	-1.99	-2.94	-1.95	-1.25	-1.84	-1.35	-1.70	-1.83	-1.69	-2.24	-1.50	-2.27	0.81460710
Arg-Val	-0.89	-1.96	-2.26	-1.59	-2.22	-1.62	-1.62	-1.86	-1.48	-2.32	-1.80	-2.65	-1.99	-1.83	-1.59	-0.98	-1.18	-1.61	-2.51	-2.71	-3.74	-1.99	-1.45	-1.63	-2.59	-1.60	-1.37	-0.69	-0.71	-1.94	-0.82	-1.30	-1.94	-1.45	-2.09	0.82685027
His-Leu	-1.33	-1.95	-2.19	-1.80	-3.37	-1.75	-1.88	-2.53	-1.85	-2.34	-2.34	-3.06	-2.19	-2.53	-2.10	-0.88	-1.34	-1.10	-2.82	-2.78	-3.82	-2.22	-1.93	-1.58	-3.05	-1.64	-1.47	-1.60	-1.02	-1.84	-1.66	-1.58	-2.43	-1.43	-2.49	0.82861643
Leu-Leu	-0.47	-2.11	-2.56	-1.64	-2.80	-1.38	-1.91	-2.47	-1.52	-2.04	-2.04	-3.06	-2.29	-2.47	-1.71	-0.80	-1.41	-1.07	-2.72	-2.39	-3.31	-2.22	-1.68	-1.15	-2.94	-1.77	-1.33	-1.94	-0.95	-1.68	-1.63	-1.78	-2.10	-0.96	-2.17	0.82984953
Leu-Met	-1.30	-1.97	-2.82	-1.69	-3.06	-1.89	-1.88	-2.57	-1.64	-2.12	-2.12	-3.01	-2.52	-2.51	-2.02	-0.84	-1.66	-1.26	-2.62	-2.82	-3.46	-2.26	-1.97	-1.31	-2.98	-1.76	-1.27	-1.79	-1.03	-1.78	-1.45	-1.68	-2.21	-1.29	-2.35	0.83127116
Butyric Acid	-2.67	-1.89	-2.85	-2.30	-3.32	-2.59	-1.75	-3.05	-1.28	-2.20	-1.90	-2.31	-1.81	-1.89	-2.20	-1.44	-1.39	-2.32	-3.41	-2.72	-3.72	-2.24	-2.20	-1.12	-2.68	-1.54	-2.43	-1.41	-2.11	-1.99	-1.35	-0.99	-2.44	-1.77	-2.05	0.83344267
Lactose	-2.71	-1.77	-2.78	-2.33	-3.17	-2.72	-2.30	-2.77	-1.53	-2.21	-1.86	-2.47	-1.96	-1.78	-2.12	-1.40	-1.44	-2.40	-3.25	-3.17	-3.70	-2.03	-1.55	-2.13	-2.51	-2.35	-2.11	-1.54	-2.52	-1.93	-1.02	-1.28	-2.70	-2.51	-1.97	0.83408136
Arabinose	-3.17	-1.96	-2.59	1.77	-3.17	-2.65	-2.14	-2.62	-1.56	-2.34	-1.82	-2.58	-2.04	-1.96	-2.16	-1.37	-1.37	-1.98	-3.43	-3.31	-3.59	-2.89	-2.61	-0.93	-2.02	-2.02	-2.31	-1.26	-1.87	-2.16	-1.10	0.15	-1.53	-1.62	-2.32	0.85366445
Glu-Glu	-1.03	-1.43	-2.75	-2.68	-3.52	-2.67	-2.71	-3.16	-1.04	-1.41	-2.64	-1.53	-2.39	-2.56	-2.91	-1.81	-1.57	-2.65	-2.79	-3.22	-3.72	-2.42	-2.40	-1.99	-2.54	-2.07	-1.06	-1.40	-2.81	-1.21	-0.95	-1.13	-2.59	-2.43	-2.66	0.85571707
Lys-Arg	-1.57	-1.49	-2.31	-1.36	-2.12	-2.06	-1.35	-2.20	-1.55	-1.63	-1.63	-2.72	-1.97	-2.10	-1.80	-0.88	-1.33	-1.43	-2.75	-2.33	-3.38	-1.87	-0.97	-1.44	-2.62	-1.79	-0.94	-0.92	-2.08	-1.70	-0.85	-1.49	-2.29	-0.76	-2.16	0.87126834
Asparagine	-2.87	-1.34	-2.47	-2.99	-3.32	-2.87	-3.72	-3.06	-1.14	-1.64	-1.49	-1.91	-1.64	-1.98	-2.15	-1.27	-1.20	-2.02	-2.45	-3.01	-3.84	-2.11	-2.14	-2.29	-2.30	-2.18	-2.21	-1.02	-3.00	-1.76	-1.28	-1.17	-2.44	-2.40	-2.01	0.87147832
Ser-Leu	-1.68	-1.69	-2.52	-2.13	-2.29	-1.95	-1.87	-2.56	-1.64	-2.18	-2.18	-2.73	-2.16	-2.15	-1.73	-1.15	-1.42	-0.95	-2.46	-2.26	-3.11	-1.97	-1.49	-1.58	-2.47	-1.98	-1.22	-1.69	-1.47	-1.99	-2.17	-1.83	-2.12	-1.37	-2.30	0.87298049
Pectin	-0.77	-1.56	-2.40	-1.69	-2.82	-2.40	-2.26	-1.61	-1.26	-2.05	-1.36	-2.16	-1.74	-1.78	-1.59	-1.27	-1.33	-1.16	-2.83	-2.56	-3.99	-2.59	-1.33	-1.45	-2.55	-1.58	-1.49	-1.00	-0.93	-1.73	-1.24	-0.60	-0.98	-1.09	-2.20	0.87623164
Ala-Pro	-3.15	-1.98	-2.67	-3.21	-3.21	-2.62	-2.79	-3.28	-1.53	-2.14	-2.09	-2.65	-2.09	-2.20	-1.97	-1.79	-1.62	-2.23	-2.89	-2.80	-3.46	-2.83	-2.58	-1.24	-2.14	-2.37	-2.56	-1.55	-1.62	-2.47	-1.66	-1.68	-3.56	-2.26	-2.60	0.87770363
Methyl Pyruvate	-1.82	-2.01	-2.91	-2.27	-3.28	-3.12	-2.29	-2.49	-1.33	-2.29	-1.78	-2.66	-1.96	-2.16	-2.16	-1.47	-1.44	-2.37	-3.41	-3.10	-3.32	-2.75	-2.25	-0.97	-2.69	-1.83	-2.17	-1.73	-1.92	-2.12	-1.28	-1.12	-2.18	-1.88	-2.31	0.87818853
Asp-Val	-2.02	-2.02	-3.01	-2.87	-3.44	-2.52	-2.73	-3.57	-1.31	-2.06	-2.08	-2.36	-2.25	-2.50	-2.63	-4.24	-1.60	-2.98	-3.44	-3.57	-3.84	-3.47	-3.44	-1.74	-3.11	-2.69	-2.98	-1.22	-2.55	-1.50	-2.29	-1.27	-1.99	-2.00	-3.18	0.88683910
Ala-Phe	-2.84	-2.62	-3.02	-3.06	-3.47	-3.05	-3.43	-3.20	-2.46	-3.10	-2.87	-3.25	-3.01	-3.08	-2.75	-1.57	-1.71	-2.22	-2.84	-3.08	-3.88	-2.67	-2.40	-2.87	-3.08	-3.20	-2.45	-2.40	-3.31	-2.35	-1.33	-2.20	-3.86	-2.89	-2.62	0.88990528
Aminoethanol	-2.39	-1.83	-2.90	-2.59	-3.44	-2.58	-1.64	-2.95	-1.02	-1.67	-1.43	-1.98	-1.61	-1.58	-1.78	-1.06	-1.11	-1.81	-3.13	-2.79	-3.78	-2.05	-2.36	-1.35	-2.66	-1.72	-1.78	-1.00	-2.27	-1.70	-1.17	0.14	-2.18	-2.29	-1.93	0.89492198
Arginine	-2.66	-1.93	-2.64	-2.35	-3.29	-2.66	-3.28	-2.89	-1.70	-2.30	-1.90	-2.55	-2.10	-2.22	-2.12	-1.34	-1.28	-1.95	-2.50	-3.24	-3.97	-2.50	-1.85	-2.32	-2.69	-2.04	-1.92	-1.12	-2.52	-1.82	-1.57	-1.47	-2.02	-2.62	-2.29	0.90969501
Propionic Acid	-2.18	-0.78	-2.89	-3.00	-3.44	-2.36	-2.05	-3.11	-0.69	-1.22	-1.30	-1.27	-1.54	-2.08	-1.43	-0.42	-1.08	-2.34	-2.31	-1.53	-3.47	-1.70	-1.01	-3.22	-1.28	-1.65	-0.97	-1.50	-2.67	-1.04	-1.61	-0.86	-2.18	-3.17	-1.71	0.91346721
Erythritol	-2.20	-1.87	-2.50	-1.51	-3.32	-2.61	-2.28	-2.64	-1.21	-1.99	-1.65	-2.34	-1.83	-1.82	-1.91	-1.54	-1.51	-2.38	-3.11	-3.24	-3.38	-2.74	-2.41	-0.99	-2.60	-1.93	-2.40	-1.50	-2.54	-1.92	-0.99	0.08	-1.92	-1.69	-2.23	0.91421921
Glu-Ser	-1.80	-1.59	-2.92	-2.50	-3.16	-2.75	-2.75	-3.08	-1.65	-1.98	-2.09	-2.19	-2.28	-3.70	-2.39	-1.12	-1.48	-2.57	-2.63	-2.80	-3.61	-2.97	-2.68	-1.79	-2.34	-2.43	-3.01	-1.02	-2.44	-2.29	-1.19	-1.17	-2.71	-1.91	-3.11	0.91504856
Inositol	-2.30	-2.06	-2.39	-2.14	-3.21	-2.90	-1.73	-2.39	-1.38	-2.11	-1.67	-2.42	-1.89	-1.80	-2.19	-1.48	-1.49	-2.23	-3.32	-3.18	-3.76	-2.69	-2.72	-0.95	-2.58	-1.76	-2.14	-1.55	-1.63	-2.07	-1.01	0.10	-1.91	-1.87	-2.20	0.92041799
Asp-Leu	-1.27	-2.02	-2.29	-1.56	-2.28	-1.54	-1.80	-2.20	-1.23	-2.31	-1.79	-2.65	-1.94	-2.07	-1.95	-1.06	-1.39	-1.22	-2.66	-2.63	-3.46	-1.92	-1.25	-1.31	-2.56	-1.38	-1.15	-1.46	-0.82	-1.37	-1.16	-1.60	-2.31	-1.06	-2.31	0.92206732
Phe-Glu	-3.29	-1.97	-3.20	-3.21	-3.40	-2.88	-2.90	-3.80	-2.06	-3.00	-3.00	-2.05	-3.11	-3.02	-3.22	-2.29	-2.56	-3.02	-3.84	-3.70	-3.72	-3.07	-2.89	-1.90	-2.83	-3.38	-2.24	-1.89	-3.59	-1.82	-1.84	-1.41	-4.21	-3.35	-3.05	0.93004261
Salicin	-1.94	-2.02	-2.82	-2.23	-3.10	-2.49	-1.15	-2.94	-0.72	-1.43	-1.28	-1.76	-1.87	-1.80	-1.91	-1.43	-1.36	-2.49	-2.66	-2.56	-3.37	-2.13	-1.85	-0.74	-2.45	-2.01	-2.39	-1.52	-2.28	-2.05	-0.66	-0.62	-2.24	-1.80	-1.83	0.93107699
Lysine	-2.91	-1.43	-2.64	-2.82	-3.07	-2.58	-3.46	-3.43	-1.45	-1.64	-2.65	-1.85	-2.45	-2.64	-2.69	-1.55	-1.44	-2.74	-2.93	-2.91	-3.78	-2.38	-2.05	-2.65	-2.01	-3.04	-2.48	-1.75	-2.83	-1.52	-1.16	-1.06	-2.12	-3.52	-2.54	0.94350965
Asp-Gln	0.48	-0.64	-0.40	0.39	-2.46	-1.37	-1.13	-0.75	0.10	-1.00	-0.51	-0.94	-0.07	-0.26	-0.31	-0.13	-1.21	-0.97	-1.49	-0.33	-1.80	-0.56	-0.21	-0.99	-0.50	-0.06	-0.63	-0.30	-0.44	0.12	-0.47	-0.35	-0.86	-0.22	-1.22	0.94440379
Malic Acid	-2.42	-1.62	-2.17	-2.50	-3.13	-2.97	-1.66	-2.61	-1.15	-2.02	-2.00	-2.31	-1.85	-1.67	-2.32	-1.34	-1.11	-2.31	-3.34	-2.75	-3.25	-2.13	-2.27	-1.44	-2.31	-1.53	-2.43	-1.09	-1.84	-1.84	-1.28	-0.54	-2.37	-2.29	-2.13	0.94463497
Glucose	0.38	0.24	-0.06	0.85	0.29	0.87	0.95	-0.08	0.24	-0.90	-0.14	-0.75	0.20	-0.52	0.47	0.62	0.70	0.84	0.49	1.02	0.95	0.38	0.84	0.45	-0.35	0.55	0.55	-0.66	0.11	-0.16	0.24	0.30	-1.52	0.27	0.74	0.94638013
Arg-Arg	-1.13	-1.57	-1.99	-1.38	-2.02	-1.48	-1.36	-1.86	-1.40	-2.04	-1.56	-2.47	-1.91	-1.97	-1.60	-0.77	-1.25	-1.38	-2.48	-2.31	-3.52	-1.89	-0.87	-1.00	-2.37	-1.56	-0.95	-0.91	-0.79	-1.53	-0.50	-1.27	-2.58	-1.31	-1.93	0.94728633
Citric Acid	-2.80	-1.74	-2.43	-1.31	-2.82	-2.37	-2.17	-1.82	-1.34	-1.75	-1.51	-1.78	-1.73	-1.63	-1.88	-1.05	-1.28	-2.11	-2.58	-2.75	-3.32	-1.20	-1.14	-1.78	-1.99	-1.84	-1.98	-1.34	-2.68	-1.49	-0.91	0.59	-3.11	-2.22	-1.73	0.96608903
MethyGalactoside	-2.77	-1.89	-2.65	-1.90	-2.97	-2.67	-1.68	-2.24	-1.37	-2.21	-1.77	-2.26	-1.85	-1.98	-1.94	-1.45	-1.59	-2.29	-3.43	-3.15	-3.34	-2.76	-2.59	-1.04	-2.20	-1.78	-2.29	-1.72	-1.66	-1.97	-1.09	-1.14	-1.63	-2.08	-1.64	0.97546206
MethylLactate	-2.58	-2.01	-2.73	-2.34	-2.87	-2.77	-2.59	-2.44	-1.38	-2.42	-1.87	-2.43	-1.76	-1.91	-2.03	-1.49	-1.57	-2.23	-3.46	-3.15	-2.97	-2.71	-2.70	-0.95	-2.71	-1.99	-2.23	-1.30	-2.24	-2.13	-1.18	-1.17	-1.74	-2.30	-2.39	0.97990768
Melibiose	-3.25	-2.01	-2.49	-2.15	-3.31	-2.90	-2.51	-2.86	-1.38	-2.20	-2.06	-2.57	-1.93	-2.07	-2.11	-1.45	-1.62	-2.34	-3.46	-3.18	-3.37	-2.97	-2.59	-1.53	-2.66	-2.04	-2.25	-1.01	-2.49	-2.31	-1.24	-1.38	-1.59	-2.47	-2.39	0.99802010
