# Supplementary material for: Decreased tryptophan metabolism in patients with autism spectrum disorders
Source: Mol Autism. 2013 Jun 3;4:16. doi: 10.1186/2040-2392-4-16 (PMC3680090; doi:10.1186/2040-2392-4-16)
Supplement: Additional file 3: Table S3 — Absorbance data of PM-M1 to M4 plates for the first 18 controls (C1-C18) and 15 patients with different conditions characterized by ID (N1-N15). Notes. The data were log-transformed before undergoing statistical analyses. The wells containing tryptophan are indicated in red. The wells with P value <0.05 are in bold. [file 2040-2392-4-16-S3.pdf]

Additional Table 3. Absorbance data of PM-M1 to M4 plates for the first 18 controls (C1-C18) and 15 patients with different conditions characterized by ID (N1-N15).

Substrate	C1	C2	C3	C4	C5	C6	C7	C8	C9	C10	C11	C12	C13	C14	C15	C16	C17	C18	N1	N2	N3	N4	N5	N6	N7	N8	N9	N10	N11	N12	N13	N14	N15	*P* value
Thr-Ser	-3.24	-1.81	-2.13	-1.35	-2.98	-2.59	-2.65	-2.55	-1.46	-1.73	-1.73	-2.69	-1.94	-1.86	-1.75	-1.31	-1.43	-1.74	-1.45	-1.73	-1.31	-2.22	-0.88	-0.47	-1.50	-0.28	-0.95	-1.33	-1.36	-1.50	-0.54	-1.14	-0.77	0.000064
Val-Ser	-1.20	-1.94	-2.77	-2.97	-3.25	-2.59	-2.50	-2.91	-1.30	-2.20	-2.20	-2.66	-2.01	-2.10	-1.97	-1.20	-1.52	-2.19	-1.68	-1.51	-0.66	-2.53	-1.79	-0.41	-1.52	-1.04	-1.10	-1.38	-1.46	-1.43	-0.75	-0.97	-1.29	0.000084
Gln-Gly	-0.65	-0.59	-1.18	-1.07	-3.16	-2.21	-1.96	-3.59	-2.66	-2.74	-3.01	-3.28	-2.53	-2.15	-2.13	-2.21	-2.46	-2.71	-0.80	-2.31	-0.59	-2.71	-1.72	0.10	-0.73	-0.61	-0.30	-1.03	-1.40	-0.60	0.40	-0.73	0.12	0.000090
Thr-Pro	-2.81	-1.73	-2.71	-1.71	-3.46	-2.51	-2.78	-2.56	-1.20	-1.82	-1.82	-2.51	-2.03	-1.97	-2.01	-1.76	-1.69	-2.37	-1.75	-1.87	-1.38	-2.55	-1.09	-0.66	-1.62	-1.22	-1.45	-1.56	-1.71	-1.56	-0.64	-1.53	-0.89	0.000228
Arg-Phe	-1.43	-2.18	-2.92	-2.05	-2.75	-2.44	-2.33	-2.78	-2.05	-2.77	-2.44	-3.21	-2.56	-2.65	-2.06	-1.25	-1.95	-1.87	-1.49	-2.11	-1.13	-3.16	-1.98	-0.99	-1.99	-0.10	-0.94	-1.06	-1.33	-1.65	-0.66	-0.66	-1.11	0.000298
Arg-Ala	-1.73	-2.05	-2.47	-1.52	-2.32	-2.39	-1.96	-2.31	-1.84	-2.52	-1.93	-2.77	-2.36	-2.21	-1.69	-0.89	-1.47	-1.60	-1.25	-1.61	-1.37	-2.63	-1.63	-0.68	-1.77	0.18	-0.49	-1.18	-1.37	-1.28	-0.45	-0.58	-0.83	0.000309
Ser-Asn	-2.22	-1.78	-2.13	-1.49	-3.15	-2.41	-2.51	-2.72	-1.22	-1.76	-1.76	-2.41	-1.86	-2.03	-1.45	-1.43	-1.37	-1.76	-1.30	-1.42	-0.89	-2.43	-1.91	-0.35	-1.67	-0.42	-1.03	-1.22	-1.36	-1.49	-0.42	-1.08	-0.94	0.000363
Gln-Glu	-0.60	-0.51	-0.99	-0.08	-3.16	-2.66	-2.12	-3.18	-1.20	-2.99	-3.06	-3.20	-2.15	-2.20	-2.40	-1.50	-2.40	-2.31	-1.08	-2.31	-0.66	-2.67	-2.37	0.69	-0.29	0.24	0.54	-0.17	-0.92	-0.08	0.91	-0.10	0.25	0.000417
Ser-Val	-1.89	-2.01	-2.34	-1.65	-3.11	-2.73	-2.63	-3.00	-1.42	-2.00	-2.00	-2.48	-2.11	-2.22	-2.04	-1.57	-1.56	-2.05	-1.40	-1.60	-1.13	-2.51	-2.70	-0.72	-1.76	-0.81	-1.16	-1.38	-1.53	-1.44	-0.56	-1.20	-1.04	0.000467
Ala-Arg	-2.21	-1.95	-2.73	-2.64	-2.69	-2.26	-2.09	-3.01	-1.82	-2.56	-2.08	-2.93	-2.37	-2.35	-2.08	-1.23	-1.57	-1.57	-1.66	-2.11	-1.43	-2.71	-2.15	-0.86	-2.13	-0.14	-0.86	-1.25	-1.43	-1.44	-0.78	-0.96	-1.19	0.000515
Ser-His	-1.65	-1.62	-2.38	-1.60	-3.37	-2.10	-2.48	-2.63	-1.74	-2.18	-2.18	-2.90	-2.37	-2.16	-1.78	-1.18	-1.26	-1.44	-1.51	-2.13	-1.28	-2.51	-1.60	-0.67	-1.80	-0.88	-0.72	-1.24	-1.43	-1.40	-0.74	-0.90	-0.76	0.000625
Ser-Glu	-2.68	-1.62	-2.47	-2.01	-3.37	-2.70	-2.60	-3.51	-1.11	-1.81	-1.81	-2.24	-2.04	-2.34	-2.16	-2.10	-1.98	-2.45	-1.71	-1.03	-1.08	-2.42	-2.92	-0.39	-1.82	-1.19	-1.60	-1.31	-1.76	-1.53	-0.59	-1.49	-1.16	0.000750
Thr-Met	-3.07	-2.18	-2.91	-2.18	-3.24	-2.54	-2.63	-3.27	-1.66	-2.14	-2.14	-3.06	-2.28	-2.23	-1.91	-1.37	-1.92	-1.99	-1.73	-2.10	-2.03	-2.64	-1.90	-1.59	-1.92	-1.99	-1.67	-1.74	-1.64	-1.61	-0.77	-1.68	-1.00	0.000824
Ser-Met	-3.38	-2.06	-2.89	-2.06	-3.01	-2.70	-3.02	-2.93	-1.87	-2.34	-2.34	-2.91	-2.47	-2.43	-1.95	-1.62	-1.94	-1.98	-1.93	-2.22	-1.78	-2.85	-2.81	-1.15	-1.76	-1.70	-1.47	-1.60	-1.74	-1.62	-1.26	-1.64	-1.20	0.000825
Thymidine	-0.64	-1.47	-1.40	-1.19	-1.82	-1.03	-0.65	-1.54	-2.60	-3.15	-2.17	-3.22	-1.86	-1.96	-1.56	-1.03	-2.66	-0.87	-0.91	-1.81	-0.67	-2.54	-0.75	-0.38	-0.85	0.17	0.00	-0.46	-1.03	-0.94	-0.24	0.28	-0.87	0.000876
Ser-Gln	-0.02	-0.30	-0.43	0.28	-3.37	-1.04	-0.66	-1.06	0.04	-0.66	-0.66	-1.33	-0.68	-0.43	-2.28	-1.03	-1.92	-1.05	0.21	0.03	0.15	-0.95	-0.68	1.01	-0.22	0.00	0.53	-0.13	-0.71	-0.07	1.00	-0.20	0.11	0.001017
Leu-Phe	-1.25	-2.24	-3.01	-2.56	-2.32	-2.02	-2.10	-3.37	-2.16	-2.53	-2.53	-3.32	-2.80	-2.81	-2.38	-1.05	-2.05	-1.20	-1.24	-1.91	-2.58	-3.24	-1.54	-0.83	-2.15	-0.20	-0.84	-0.79	-1.25	-1.50	-0.62	-0.58	-1.11	0.001162
Ala-His	-2.10	-2.02	-2.44	-1.94	-2.77	-2.35	-2.26	-2.66	-1.65	-2.55	-1.84	-3.07	-2.18	-2.29	-2.15	-1.23	-1.37	-1.99	-1.55	-1.79	-1.93	-2.57	-2.62	-0.30	-1.79	-0.48	-0.92	-1.06	-1.38	-1.43	-0.54	-1.44	-0.96	0.001212
Lysine	-2.91	-1.43	-2.64	-2.82	-3.07	-2.58	-3.46	-3.43	-1.45	-1.64	-2.65	-1.85	-2.45	-2.64	-2.69	-1.55	-1.44	-2.74	-1.74	-1.43	-1.22	-2.02	-2.77	-0.77	-1.62	-2.43	-1.67	-1.70	-1.92	-1.56	-0.65	-2.00	-1.14	0.001235
Arg-Arg	-1.13	-1.57	-1.99	-1.38	-2.02	-1.48	-1.36	-1.86	-1.40	-2.04	-1.56	-2.47	-1.91	-1.97	-1.60	-0.77	-1.25	-1.38	-1.11	-1.59	-0.87	-2.40	-1.02	-0.31	-1.51	0.47	-0.06	-0.64	-1.17	-1.23	-0.19	-0.33	-0.74	0.001262
Ser-Asp	-2.40	-1.93	-2.47	-1.59	-3.41	-2.46	-2.73	-2.65	-1.35	-2.02	-2.02	-2.54	-2.03	-2.11	-1.98	-1.47	-1.52	-1.95	-1.47	-1.25	-1.66	-2.56	-2.63	-0.55	-1.79	-0.75	-1.21	-1.30	-1.58	-1.60	-0.74	-1.50	-1.16	0.001290
Succinic Acid	-2.93	-1.30	-1.96	-1.63	-3.06	-2.62	-0.94	-2.27	-0.60	-1.68	-2.51	-1.66	-1.69	-1.55	-2.34	-1.63	-1.45	-2.25	-0.43	-2.01	-0.52	-2.06	-2.21	0.31	-0.83	0.15	-0.66	-2.03	-1.31	-1.47	0.10	0.31	-0.61	0.001445
Acetic Acid	-1.04	-1.19	-1.87	-1.37	-1.93	-1.46	-0.61	-2.14	-0.98	-1.75	-1.36	-2.42	-1.49	-1.60	-1.75	-0.77	-1.17	-1.51	-1.14	-1.45	-0.44	-2.41	-0.48	-0.33	-1.07	0.31	0.23	-1.08	-1.09	-0.84	0.04	0.29	-0.53	0.001519
Arg-Lys	-0.73	-1.78	-2.17	-1.55	-2.12	-1.56	-1.54	-2.14	-1.60	-2.54	-1.62	-2.67	-1.97	-1.94	-1.75	-0.99	-1.33	-1.34	-1.19	-1.65	-0.97	-2.72	-1.11	-0.33	-1.74	0.32	-0.32	-0.68	-1.19	-1.19	-0.32	-0.26	-0.63	0.001554
Leucine	-1.49	-2.15	-2.74	-1.98	-2.63	-1.67	-1.87	-2.63	-1.70	-2.47	-2.16	-2.79	-2.20	-2.37	-1.98	-0.91	-1.25	-1.22	-1.19	-1.97	-1.24	-2.78	-1.59	-0.82	-1.88	-0.25	-0.76	-0.84	-1.44	-1.53	-0.72	-0.80	-1.42	0.001606
Malic Acid	-2.42	-1.62	-2.17	-2.50	-3.13	-2.97	-1.66	-2.61	-1.15	-2.02	-2.00	-2.31	-1.85	-1.67	-2.32	-1.34	-1.11	-2.31	-1.29	-2.24	-1.08	-2.06	-2.51	-0.35	-1.03	-1.40	-1.36	-1.48	-1.76	-1.33	-0.22	0.12	-0.74	0.001755
His-Leu	-1.33	-1.95	-2.19	-1.80	-3.37	-1.75	-1.88	-2.53	-1.85	-2.34	-2.34	-3.06	-2.19	-2.53	-2.10	-0.88	-1.34	-1.10	-1.16	-1.99	-2.04	-2.41	-1.43	-0.71	-1.77	-0.19	-0.86	-0.94	-1.51	-1.47	-0.50	-0.93	-1.30	0.001778
Ser-Pro	-2.85	-1.79	-2.59	-2.01	-2.95	-2.62	-2.63	-2.53	-1.24	-1.95	-1.95	-2.21	-2.05	-1.99	-2.03	-1.69	-1.63	-2.27	-1.60	-1.70	-1.41	-2.50	-2.83	-0.63	-1.80	-0.80	-1.41	-1.39	-1.63	-1.56	-0.75	-1.67	-0.86	0.001823
Histidine	-2.20	-1.66	-2.45	-1.95	-3.70	-3.01	-2.69	-2.72	-1.43	-2.24	-1.84	-2.49	-1.84	-1.72	-2.23	-1.50	-1.24	-2.40	-1.35	-1.87	-1.29	-2.11	-2.80	-0.40	-1.74	-1.08	-1.35	-1.76	-1.64	-1.29	-0.37	-1.70	-0.85	0.001875
Pro-Arg	-1.27	-1.63	-2.22	-1.41	-3.52	-1.89	-2.09	-2.40	-1.30	-1.59	-1.59	-2.77	-1.92	-2.07	-2.04	-1.02	-1.48	-1.31	-1.14	-1.50	-1.10	-2.74	-1.69	-0.93	-1.65	0.22	-0.64	-0.94	-1.12	-1.31	-0.70	-0.44	-0.72	0.001926
Alaninamide	-2.29	-2.40	-2.94	-2.40	-3.16	-2.51	-3.02	-3.00	-1.79	-2.47	-2.33	-2.89	-2.24	-2.35	-2.05	-1.57	-1.69	-2.27	-1.90	-2.13	-1.79	-2.70	-2.82	-1.10	-1.89	-1.74	-1.45	-1.91	-1.94	-1.81	-0.89	-1.98	-1.43	0.002047
Pro-Lys	-1.61	-1.81	-2.11	-1.44	-3.59	-1.66	-1.74	-2.27	-1.21	-1.69	-1.69	-2.37	-1.69	-1.70	-2.02	-0.91	-1.14	-1.33	-1.26	-1.66	-1.17	-2.43	-1.79	-0.54	-1.59	0.22	-0.70	-0.72	-1.04	-1.27	-0.37	-0.31	-0.77	0.002263
Asp-Leu	-1.27	-2.02	-2.29	-1.56	-2.28	-1.54	-1.80	-2.20	-1.23	-2.31	-1.79	-2.65	-1.94	-2.07	-1.95	-1.06	-1.39	-1.22	-1.16	-1.69	-1.03	-2.84	-0.97	-0.46	-1.84	-0.08	-0.69	-0.69	-1.30	-1.44	-0.76	-0.71	-1.16	0.002335
Gly-His	-2.57	-1.93	-2.47	-2.02	-3.70	-2.63	-2.57	-3.22	-1.63	-2.33	-2.33	-2.68	-2.32	-2.31	-2.59	-1.55	-1.88	-2.37	-1.51	-2.31	-2.16	-2.52	-2.91	-1.27	-1.76	-1.77	-1.47	-1.58	-1.84	-1.53	-0.69	-2.01	-1.18	0.002476
Glucose-1-P	-3.49	-2.01	-1.58	-0.17	-3.31	-1.74	-0.81	-1.98	-0.80	-1.65	-1.50	-1.72	-0.50	-0.99	-1.14	-0.72	-0.67	-1.18	-0.44	-0.97	-0.39	-1.32	-1.83	0.40	-0.91	-0.51	-0.35	-1.11	-0.82	-0.44	-0.14	0.51	-0.39	0.002535
Fructose-6-P	-1.12	-0.96	-0.72	-0.47	-3.08	-1.12	0.61	-0.86	-0.56	-1.34	-0.97	-1.55	-0.04	0.02	-0.75	-0.51	-0.38	-1.20	0.24	-0.79	0.18	-0.68	-1.19	0.93	-0.68	0.33	0.57	-0.47	-0.29	-0.09	0.55	0.82	0.04	0.002700
Gly-Arg	-1.61	-1.98	-2.47	-1.79	-2.33	-1.91	-1.84	-2.56	-1.80	-2.43	-2.09	-2.87	-2.28	-2.13	-1.66	-1.03	-1.25	-1.40	-1.51	-2.01	-1.36	-2.78	-2.13	-0.53	-1.68	-0.09	-0.75	-0.89	-1.25	-1.43	-0.55	-0.80	-1.01	0.002733
His-Ser	-2.36	-2.05	-2.58	-1.65	-3.22	-2.64	-2.77	-3.02	-1.65	-2.01	-2.01	-2.91	-2.28	-2.21	-2.09	-1.36	-1.58	-1.83	-1.42	-1.78	-1.85	-2.48	-2.83	-0.68	-2.08	-1.19	-1.25	-1.43	-1.71	-1.51	-0.90	-1.68	-1.32	0.002743
His-Glu	-3.02	-1.81	-2.47	-1.65	-3.70	-2.66	-2.80	-3.37	-1.20	-2.13	-2.13	-1.92	-2.40	-2.95	-2.82	-1.85	-1.70	-2.70	-1.70	-1.79	-1.69	-2.01	-2.92	-0.14	-1.57	-2.05	-1.93	-1.82	-2.20	-1.51	-0.25	-1.77	-0.86	0.002774
Ala-Glu	-3.01	-1.84	-2.78	-2.10	-3.44	-2.53	-2.56	-2.89	-1.26	-1.83	-2.13	-2.04	-2.16	-2.28	-2.62	-2.66	-2.26	-2.76	-1.99	-1.86	-1.46	-2.60	-2.82	-0.35	-2.02	-1.28	-1.44	-2.09	-2.05	-1.89	-0.65	-1.92	-1.18	0.002787
Ser-Ser	-3.13	-1.87	-2.29	-1.68	-2.94	-2.87	-2.41	-2.66	-1.40	-1.66	-1.66	-2.55	-1.96	-1.85	-1.78	-1.55	-1.41	-1.83	-1.52	-1.68	-1.28	-2.53	-2.83	-0.91	-1.63	-0.45	-0.93	-1.16	-1.59	-1.49	-0.66	-1.23	-1.10	0.002793
Pro-Ile	-3.12	-1.96	-2.81	-1.87	-3.64	-2.54	-2.47	-3.37	-1.22	-2.02	-2.02	-3.74	-2.36	-2.34	-2.60	-2.00	-1.81	-2.59	-1.72	-1.38	-1.72	-2.63	-3.12	-0.56	-1.67	-1.91	-1.81	-1.86	-2.08	-1.88	-0.65	-1.76	-0.95	0.002883
Threonine	-2.82	-1.75	-2.46	-1.91	-2.92	-2.49	-2.55	-2.24	-1.36	-2.06	-1.87	-2.36	-2.01	-1.77	-1.93	-1.48	-1.31	-2.20	-1.38	-1.91	-1.52	-2.47	-2.76	-0.54	-1.84	-0.76	-1.06	-1.30	-1.70	-1.48	-0.46	-1.23	-0.80	0.002962
Leu-Met	-1.30	-1.97	-2.82	-1.69	-3.06	-1.89	-1.88	-2.57	-1.64	-2.12	-2.12	-3.01	-2.52	-2.51	-2.02	-0.84	-1.66	-1.26	-1.43	-2.04	-2.31	-2.73	-1.48	-0.79	-1.84	-0.93	-0.76	-0.88	-1.28	-1.28	-0.47	-0.72	-1.06	0.003025
Ala-Gly	-2.05	-2.05	-2.51	-1.91	-2.72	-2.37	-2.19	-2.59	-1.34	-2.47	-1.76	-2.52	-2.02	-2.18	-1.86	-1.38	-1.38	-2.08	-1.31	-2.10	-1.86	-2.60	-2.51	-0.40	-1.72	-1.52	-0.97	-0.90	-1.37	-1.35	-0.62	-1.33	-1.17	0.003106
Arg-Tyr	-0.70	-1.54	-2.04	-1.59	-2.20	-1.92	-1.60	-2.00	-1.35	-2.08	-1.80	-2.29	-1.13	-1.20	-1.53	-0.91	-1.13	-1.44	-1.25	-1.84	-0.90	-2.03	-1.37	-0.48	-1.34	0.27	-0.52	-0.31	-1.35	-1.35	-0.34	-0.45	-0.82	0.003110
Pro-Glu	-3.04	-1.78	-2.52	-1.79	-3.54	-2.58	-2.47	-3.34	-0.99	-2.02	-2.02	-1.87	-2.50	-2.48	-2.98	-2.23	-2.47	-2.65	-1.63	-0.95	-1.32	-2.13	-2.76	-0.89	-1.45	-2.20	-1.82	-2.33	-2.53	-1.71	-0.35	-2.01	-0.76	0.003143
Methionine	-3.07	-2.01	-2.95	-2.71	-3.21	-2.63	-2.91	-3.66	-2.05	-2.25	-2.31	-2.67	-2.53	-2.55	-2.21	-1.75	-1.69	-2.24	-1.80	-2.30	-1.62	-2.66	-2.75	-1.50	-1.97	-2.53	-1.75	-2.29	-1.90	-1.73	-1.16	-2.18	-1.00	0.003147
Arg-Val	-0.89	-1.96	-2.26	-1.59	-2.22	-1.62	-1.62	-1.86	-1.48	-2.32	-1.80	-2.65	-1.99	-1.83	-1.59	-0.98	-1.18	-1.61	-1.05	-1.56	-1.06	-2.65	-1.89	-0.54	-1.51	-0.22	-0.79	-0.83	-1.29	-1.38	-0.35	-0.69	-0.83	0.003198
Lys-Arg	-1.57	-1.49	-2.31	-1.36	-2.12	-2.06	-1.35	-2.20	-1.55	-1.63	-1.63	-2.72	-1.97	-2.10	-1.80	-0.88	-1.33	-1.43	-1.28	-1.75	-1.97	-2.67	-1.02	-0.43	-1.71	0.44	-0.25	-0.43	-1.13	-1.10	-0.29	-0.31	-0.69	0.003349
Lys-Trp	-2.94	-1.82	-2.75	-1.93	-2.46	-2.63	-2.73	-3.07	-1.39	-2.05	-2.05	-2.71	-2.04	-1.98	-2.09	-1.48	-1.55	-2.43	-1.55	-2.61	-2.20	-2.63	-3.12	-0.32	-1.47	0.27	-0.55	-0.48	-1.21	-1.58	-0.26	-0.56	-0.81	0.003384
Tyr-Gln	-0.49	-0.38	-1.25	-0.71	-2.43	-1.21	-0.92	-1.38	-0.01	-1.20	-1.20	-0.99	-0.17	-0.50	-1.49	-0.62	-1.25	-1.15	-0.09	-0.08	-0.64	-1.31	-0.56	0.79	-0.26	-0.50	0.23	-0.85	-1.12	-0.73	0.99	-0.29	0.25	0.003449
Val-Arg	-1.16	-1.77	-2.06	-1.31	-3.41	-1.32	-1.22	-2.15	-1.34	-1.79	-1.79	-2.80	-1.69	-2.11	-1.80	-1.00	-1.37	-1.25	-1.25	-1.77	-1.01	-2.51	-1.47	-0.51	-1.53	0.16	-0.64	-0.56	-1.22	-1.37	-0.42	-0.67	-0.80	0.003635
Tyrosine	-2.33	-1.61	-2.22	-2.51	-3.56	-2.54	-2.71	-2.81	-1.37	-2.11	-2.35	-2.13	-1.92	-1.98	-2.79	-1.61	-1.49	-2.59	-1.65	-1.81	-1.50	-2.65	-2.78	-0.90	-1.31	-1.64	-1.63	-1.34	-1.93	-1.72	-0.67	-1.95	-1.06	0.003688
MethyGalactoside	-2.08	-1.85	-2.70	-1.88	-2.87	-2.62	-1.82	-2.66	-1.10	-2.43	-1.87	-2.40	-1.84	-1.85	-1.97	-1.40	-1.60	-2.27	-1.27	-1.56	-1.15	-2.68	-2.35	-0.92	-1.08	-2.10	-1.16	-2.07	-1.65	-1.36	-0.34	-0.15	-0.72	0.003718
Met-Thr	-3.31	-1.97	-3.00	-1.88	-3.16	-2.77	-2.56	-3.38	-1.75	-2.21	-2.21	-2.62	-2.32	-2.16	-2.32	-1.42	-2.03	-2.33	-1.78	-2.67	-1.98	-2.61	-2.68	-1.24	-1.81	-1.94	-1.58	-1.76	-1.74	-1.66	-0.95	-1.89	-1.01	0.003772
Ser-Leu	-1.68	-1.69	-2.52	-2.13	-2.29	-1.95	-1.87	-2.56	-1.64	-2.18	-2.18	-2.73	-2.16	-2.15	-1.73	-1.15	-1.42	-0.95	-1.49	-2.04	-1.42	-2.58	-1.51	-0.87	-1.99	-0.18	-0.99	-1.28	-1.30	-1.43	-1.04	-1.08	-1.32	0.003881
Ala-Ile	-2.08	-1.87	-2.40	-1.83	-2.67	-2.28	-1.99	-2.81	-1.42	-2.54	-1.88	-2.51	-1.97	-2.20	-1.96	-1.28	-1.47	-1.56	-1.13	-1.68	-1.33	-2.83	-2.66	-0.33	-1.51	-0.29	-1.05	-1.67	-1.70	-1.60	-0.66	-0.91	-0.74	0.003948
Pro-Ser	-2.93	-1.99	-2.52	-1.83	-3.04	-2.61	-2.63	-2.84	-1.37	-1.97	-1.97	-2.56	-1.93	-2.14	-2.04	-1.54	-1.63	-2.30	-1.55	-1.64	-1.36	-2.61	-2.91	-0.72	-2.02	-1.37	-1.38	-1.51	-1.88	-1.61	-0.92	-1.73	-1.12	0.003972
Gly-Met	-2.79	-2.11	-2.94	-2.39	-3.16	-2.92	-2.62	-3.20	-1.98	-2.43	-2.43	-2.89	-2.59	-2.69	-2.49	-1.58	-1.77	-2.18	-1.84	-2.94	-2.10	-2.74	-2.99	-0.98	-1.78	-2.10	-1.65	-1.58	-1.84	-1.66	-0.79	-1.98	-1.17	0.003982
Ala-Met	-2.31	-2.29	-3.07	-2.46	-2.82	-2.51	-2.70	-3.18	-1.95	-2.50	-2.42	-3.24	-2.40	-2.55	-2.18	-1.30	-1.41	-2.02	-1.74	-2.25	-2.04	-2.94	-2.81	-0.88	-1.83	-2.02	-1.54	-1.42	-1.80	-1.72	-1.01	-1.76	-1.31	0.004024
Ala-Asp	-2.53	-2.18	-2.77	-1.94	-3.32	-2.51	-2.43	-2.86	-1.45	-2.53	-2.00	-2.81	-2.03	-2.27	-2.07	-1.76	-1.57	-2.27	-1.74	-2.11	-1.37	-2.75	-2.88	-0.66	-1.94	-1.37	-1.51	-1.62	-1.84	-1.61	-0.69	-1.80	-1.38	0.004106
Ala-Asn	-2.98	-2.05	-2.80	-2.13	-3.25	-2.61	-2.63	-2.98	-1.47	-2.31	-1.88	-2.38	-2.05	-2.20	-2.08	-1.70	-1.35	-2.10	-1.87	-1.80	-1.61	-2.61	-2.95	-0.62	-1.89	-1.57	-1.67	-1.75	-1.69	-1.47	-0.69	-1.54	-1.04	0.004107
Serine	-2.54	-1.88	-2.39	-2.06	-3.21	-2.41	-2.42	-2.48	-1.47	-2.51	-2.50	-2.71	-2.01	-1.98	-2.04	-1.45	-1.44	-2.07	-1.55	-1.86	-1.83	-2.51	-2.62	-1.12	-2.11	-1.35	-1.18	-1.64	-1.79	-1.92	-0.87	-1.51	-1.19	0.004115
Pro-Val	-3.04	-1.87	-2.93	-2.10	-3.38	-2.50	-2.65	-3.54	-1.12	-1.86	-1.86	-2.32	-2.16	-2.32	-2.23	-2.11	-1.84	-2.70	-1.74	-0.87	-1.31	-2.82	-2.58	-1.09	-1.87	-1.64	-1.99	-2.16	-1.89	-1.83	-0.68	-2.08	-0.79	0.004188
Thr-Phe	-2.56	-2.40	-3.15	-2.03	-3.22	-2.79	-2.54	-3.37	-2.32	-2.51	-2.51	-3.35	-3.00	-2.79	-2.03	-1.83	-2.47	-2.36	-1.79	-2.45	-2.56	-3.37	-1.41	-1.16	-2.51	-2.33	-1.94	-2.11	-1.97	-2.02	-1.15	-2.32	-1.30	0.004202
Thr-Gly	-2.84	-2.01	-2.84	-2.57	-3.38	-2.65	-2.71	-3.22	-1.65	-2.01	-2.01	-2.70	-2.30	-2.27	-1.91	-1.48	-1.71	-1.93	-1.70	-2.39	-2.45	-2.53	-2.45	-0.93	-1.95	-1.46	-1.65	-1.74	-1.56	-1.61	-0.98	-1.94	-1.25	0.004338
Ala-Val	-2.13	-2.04	-2.69	-1.73	-3.06	-2.42	-2.54	-2.50	-1.52	-2.27	-1.79	-2.48	-2.11	-2.11	-1.89	-1.46	-1.61	-2.10	-1.54	-1.94	-1.52	-2.51	-2.63	-0.64	-1.80	-1.84	-1.06	-1.91	-1.82	-1.34	-0.63	-1.34	-0.87	0.004630
Met-Arg	-1.74	-1.80	-2.72	-1.59	-3.34	-2.11	-2.01	-2.74	-1.76	-2.14	-2.14	-3.16	-2.51	-2.44	-2.04	-1.03	-1.77	-1.45	-1.56	-2.36	-2.03	-2.90	-2.45	-0.99	-1.72	0.24	-0.67	-0.78	-1.40	-1.42	-0.49	-0.54	-0.80	0.004949
Pectin	-0.77	-1.56	-2.40	-1.69	-2.82	-2.40	-2.26	-1.61	-1.26	-2.05	-1.36	-2.16	-1.74	-1.78	-1.59	-1.27	-1.33	-1.16	-1.03	-1.76	-1.07	-3.31	-0.72	-0.09	-1.16	-0.09	-0.48	-1.30	-1.24	-1.05	-0.33	0.06	-0.89	0.004989
Ala-Phe	-2.84	-2.62	-3.02	-3.06	-3.47	-3.05	-3.43	-3.20	-2.46	-3.10	-2.87	-3.25	-3.01	-3.08	-2.75	-1.57	-1.71	-2.22	-2.02	-2.60	-2.84	-3.20	-3.18	-1.47	-2.36	-2.62	-1.92	-2.05	-2.08	-2.02	-1.51	-2.18	-1.59	0.005055
Ser-Gly	-2.18	-2.10	-2.56	-1.79	-3.38	-2.63	-2.65	-2.62	-1.74	-2.15	-2.15	-2.85	-2.08	-2.10	-1.95	-1.38	-1.43	-1.70	-1.48	-1.95	-1.48	-2.47	-2.99	-0.72	-1.87	-1.15	-1.22	-1.41	-1.65	-1.77	-0.90	-1.74	-1.31	0.005148
Glu-Asp	-0.82	-1.70	-2.57	-2.61	-3.59	-2.78	-2.66	-2.40	-0.94	-1.45	-1.69	-1.76	-2.34	-2.31	-2.50	-1.41	-1.28	-2.75	-1.10	-1.10	-0.86	-2.10	-2.87	-0.20	-1.26	-1.99	-1.19	-1.56	-2.03	-1.13	-0.01	-1.44	-0.78	0.005200
Val-Lys	-1.42	-1.91	-2.65	-2.91	-3.18	-2.59	-2.48	-3.66	-1.32	-1.94	-1.94	-2.46	-1.82	-1.91	-2.02	-0.91	-1.77	-2.75	-1.72	-1.57	-1.34	-2.53	-1.82	-1.64	-1.68	-2.49	-1.34	-1.46	-1.42	-1.76	-0.60	-1.35	-1.04	0.005279
Alanine	-2.37	-2.29	-2.94	-2.24	-3.16	-2.62	-2.84	-3.08	-1.69	-2.37	-2.20	-2.72	-2.28	-2.63	-2.29	-1.48	-1.53	-2.28	-1.76	-2.62	-1.92	-2.69	-2.95	-0.98	-1.94	-1.82	-1.36	-1.64	-1.70	-1.63	-0.77	-1.98	-1.31	0.005326
Arg-Met	-2.00	-2.08	-2.59	-1.82	-3.47	-2.71	-2.66	-3.01	-1.82	-2.68	-2.42	-2.95	-2.40	-2.39	-2.22	-1.66	-1.58	-2.20	-1.56	-2.64	-1.84	-2.77	-2.77	-0.87	-1.94	-1.76	-1.45	-1.94	-1.68	-1.60	-0.72	-1.83	-1.22	0.005359
Serine	-2.52	-1.87	-2.15	-1.34	-2.50	-2.40	-2.66	-2.40	-1.35	-2.25	-1.82	-2.44	-2.08	-1.99	-1.92	-1.34	-1.41	-1.92	-1.44	-1.45	-1.40	-2.44	-2.85	-0.84	-1.89	-0.42	-0.86	-1.39	-1.64	-1.58	-0.60	-1.41	-1.12	0.005414
Hydroxy-Proline	-1.99	-2.03	-2.52	-2.02	-3.24	-2.65	-2.88	-2.43	-1.49	-2.11	-1.89	-2.65	-1.94	-1.99	-1.84	-1.27	-1.15	-1.96	-1.40	-1.39	-1.47	-2.66	-2.65	-0.39	-1.89	-1.77	-1.25	-1.29	-1.56	-1.48	-0.75	-1.67	-1.10	0.005611
acetyneuraminic acid	-1.93	-1.84	-2.35	-1.61	-3.07	-2.51	-1.22	-2.30	-1.22	-2.30	-1.71	-2.24	-1.76	-1.86	-1.71	-1.31	-1.41	-2.14	-1.06	-1.53	-1.17	-2.73	-2.37	-0.80	-1.47	-0.94	-1.30	-1.80	-1.54	-1.16	-0.24	-0.02	-0.81	0.005942
Val-Met	-1.15	-2.02	-3.15	-2.29	-3.24	-2.56	-2.74	-3.78	-1.54	-2.16	-2.16	-2.68	-2.40	-2.02	-1.92	-1.06	-1.88	-2.56	-1.52	-2.04	-1.68	-2.74	-1.88	-1.01	-2.02	-1.49	-1.35	-2.66	-1.51	-1.71	-0.51	-1.26	-1.20	0.006053
Glutamic Acid	-2.34	-2.18	-2.43	-1.84	-2.88	-2.52	-2.74	-2.55	-1.61	-2.51	-1.90	-2.83	-2.02	-2.06	-2.03	-1.38	-1.45	-2.16	-1.45	-1.81	-1.40	-2.98	-2.68	-0.65	-1.91	-1.34	-1.45	-1.76	-1.71	-1.56	-0.55	-1.69	-1.18	0.006163
Met-His	-2.84	-1.97	-2.67	-2.30	-3.59	-2.70	-2.65	-3.11	-1.73	-2.10	-2.10	-3.06	-2.46	-2.78	-2.40	-1.41	-1.53	-1.83	-1.56	-2.67	-2.08	-2.67	-2.77	-0.96	-1.64	-2.22	-1.47	-1.61	-1.74	-1.54	-0.68	-1.93	-1.12	0.006260
Asp-Glu	-2.63	-1.44	-2.61	-2.56	-3.13	-2.87	-2.95	-2.93	-1.19	-1.70	-2.58	-1.56	-2.43	-2.40	-2.87	-1.97	-2.29	-2.68	-2.13	-1.16	-1.01	-1.85	-2.69	-0.63	-1.40	-2.40	-2.03	-2.54	-2.64	-1.33	-0.45	-2.04	-0.60	0.006340
Thr-Leu	-1.64	-2.14	-2.77	-1.77	-2.22	-1.49	-1.87	-2.52	-1.62	-2.13	-2.13	-2.89	-2.23	-2.17	-1.81	-0.93	-1.53	-1.11	-1.45	-2.02	-1.59	-2.80	-0.75	-0.85	-1.99	-0.23	-1.22	-1.14	-1.42	-1.65	-0.95	-0.98	-1.19	0.006419
Gly-Asp	-1.82	-1.95	-2.78	-2.40	-3.40	-2.70	-2.91	-2.95	-1.35	-2.40	-2.04	-2.39	-2.28	-2.31	-2.40	-1.42	-1.36	-2.33	-1.93	-1.95	-1.78	-2.42	-2.55	-0.74	-1.72	-2.28	-1.58	-1.51	-1.86	-1.68	-0.90	-2.04	-1.16	0.006601
Pro-Asp	-2.85	-1.98	-2.79	-1.86	-3.41	-2.58	-2.58	-3.21	-1.24	-1.78	-1.78	-2.22	-1.74	-2.51	-2.31	-1.90	-1.83	-2.43	-1.61	-1.48	-1.31	-2.79	-2.84	-0.49	-1.77	-1.92	-1.49	-1.92	-2.08	-1.59	-0.56	-1.77	-0.89	0.006684
Leu-His	-0.64	-2.06	-2.46	-1.70	-2.74	-1.64	-1.83	-2.53	-1.82	-2.20	-2.20	-3.37	-2.48	-2.46	-2.06	-0.96	-1.50	-1.10	-1.42	-2.11	-2.09	-2.59	-1.41	-0.77	-1.98	-0.18	-0.89	-0.89	-1.27	-1.43	-0.63	-0.70	-1.24	0.006695
Arginine	-2.66	-1.93	-2.64	-2.35	-3.29	-2.66	-3.28	-2.89	-1.70	-2.30	-1.90	-2.55	-2.10	-2.22	-2.12	-1.34	-1.28	-1.95	-1.59	-2.31	-1.39	-2.66	-2.87	-0.67	-1.89	-1.94	-1.67	-1.50	-1.69	-1.55	-0.55	-1.64	-1.07	0.006806
Ser-Tyr	-1.88	-1.74	-1.81	-1.47	-2.42	-2.63	-2.13	-2.26	-1.34	-1.62	-1.62	-2.14	-1.03	-1.14	-1.53	-1.29	-1.19	-1.63	-1.52	-1.56	-1.20	-1.92	-2.11	-0.52	-1.44	-0.15	-0.84	-0.89	-1.63	-1.44	-0.60	-1.17	-1.04	0.006898
Met-Leu	-1.81	-1.96	-2.84	-1.98	-3.54	-2.04	-2.01	-2.90	-1.76	-2.06	-2.06	-2.93	-2.42	-2.58	-2.10	-0.95	-1.27	-1.29	-1.67	-2.27	-2.18	-2.83	-1.38	-1.15	-1.92	-0.79	-1.13	-1.43	-1.55	-1.45	-0.88	-1.11	-1.21	0.007048
Tyr-Lys	-0.67	-1.85	-2.18	-1.65	-2.65	-1.89	-1.54	-1.86	-1.55	-1.82	-1.82	-2.37	-1.12	-1.23	-1.49	-1.28	-1.37	-1.34	-1.49	-1.93	-1.07	-2.06	-1.59	-0.69	-1.46	0.27	-0.90	-0.72	-1.41	-1.34	-0.49	-0.62	-0.77	0.007051
Methyl Pyruvate	-1.82	-2.01	-2.91	-2.27	-3.28	-3.12	-2.29	-2.49	-1.33	-2.29	-1.78	-2.66	-1.96	-2.16	-2.16	-1.47	-1.44	-2.37	-1.45	-2.14	-1.06	-2.90	-2.54	-0.66	-1.60	-1.38	-1.86	-2.24	-1.73	-1.56	-0.67	-0.67	-1.13	0.007071
Arg-Ile	-0.68	-1.66	-2.18	-1.25	-2.13	-1.82	-1.60	-2.22	-1.38	-1.79	-1.80	-2.59	-1.70	-1.65	-1.68	-0.95	-1.18	-1.70	-0.81	-1.39	-0.81	-2.67	-2.23	-0.42	-1.40	-0.01	-0.60	-1.16	-1.29	-1.25	-0.36	-0.27	-0.73	0.007300
Aspartic Acid	-3.20	-1.68	-2.52	-2.32	-3.16	-2.65	-3.01	-3.68	-1.34	-1.92	-2.45	-2.14	-2.45	-2.61	-2.84	-2.08	-1.82	-2.67	-1.75	-1.80	-0.91	-2.51	-2.88	-0.74	-1.83	-2.32	-2.12	-2.66	-2.36	-1.49	-0.43	-2.05	-0.90	0.007392
Leu-Arg	-1.20	-1.98	-2.41	-1.83	-2.97	-1.39	-1.42	-2.16	-1.58	-2.11	-2.11	-2.97	-2.42	-2.27	-1.85	-0.74	-1.30	-0.89	-1.07	-1.91	-1.96	-2.74	-1.69	-0.50	-1.84	0.13	-0.49	-0.53	-1.12	-1.37	-0.45	-0.44	-1.13	0.007403
Leu-Ser	-1.28	-2.00	-2.30	-1.71	-3.43	-1.79	-1.67	-2.26	-1.62	-2.09	-2.09	-2.92	-2.13	-1.88	-1.64	-0.93	-1.25	-1.10	-1.34	-1.83	-1.96	-2.43	-1.65	-0.60	-1.78	-0.06	-0.73	-0.97	-1.26	-1.52	-0.74	-0.93	-1.34	0.007432
His-Pro	-2.10	-1.65	-2.41	-2.17	-3.21	-2.71	-2.55	-3.51	-1.24	-1.86	-1.86	-2.47	-2.11	-2.18	-2.27	-1.45	-1.57	-2.42	-1.41	-2.08	-1.55	-2.38	-2.84	-0.61	-1.85	-1.74	-1.74	-1.77	-1.75	-1.56	-0.75	-1.44	-0.80	0.007471
Thr-Glu	-2.86	-1.69	-2.90	-2.42	-3.24	-2.80	-2.79	-3.63	-0.98	-1.89	-1.89	-1.89	-2.74	-2.48	-2.69	-1.77	-2.12	-2.62	-2.16	-2.00	-1.44	-2.40	-3.05	-0.98	-1.55	-2.02	-1.91	-1.97	-1.84	-1.95	-0.86	-1.98	-0.77	0.007634
Lactitol	-2.60	-1.83	-2.56	-2.47	-3.47	-2.73	-1.80	-2.85	-1.37	-2.32	-1.96	-2.42	-1.96	-1.85	-2.18	-1.40	-1.41	-2.48	-1.44	-1.85	-1.48	-2.84	-2.84	-0.61	-1.42	-2.16	-1.58	-1.61	-1.78	-1.52	-0.53	-0.44	-1.09	0.007716
Isoleucine	-2.73	-1.97	-2.76	-2.81	-3.37	-2.46	-2.49	-3.61	-1.30	-2.05	-1.95	-2.29	-2.03	-2.12	-2.87	-1.59	-1.35	-2.70	-1.32	-1.29	-1.40	-2.40	-2.90	-0.49	-1.60	-2.52	-1.98	-2.07	-2.29	-1.84	-0.54	-1.72	-0.86	0.007897
Thr-Ala	-1.94	-1.97	-2.63	-1.89	-2.76	-2.62	-2.56	-2.75	-1.46	-1.89	-1.89	-2.83	-1.98	-2.06	-1.97	-1.35	-1.39	-1.73	-1.54	-2.24	-1.74	-2.48	-2.67	-0.95	-1.76	-1.25	-1.25	-1.41	-1.52	-1.64	-0.66	-1.40	-1.06	0.008055
Glu-Ser	-1.80	-1.59	-2.92	-2.50	-3.16	-2.75	-2.75	-3.08	-1.65	-1.98	-2.09	-2.19	-2.28	-3.70	-2.39	-1.12	-1.48	-2.57	-2.07	-1.59	-1.56	-2.57	-2.85	-0.83	-1.75	-1.48	-1.88	-1.64	-2.17	-1.72	-1.04	-1.89	-1.05	0.008554
His-Val	-2.90	-1.86	-2.42	-1.95	-2.50	-2.56	-2.63	-3.40	-1.37	-1.77	-1.77	-2.83	-1.92	-2.23	-2.51	-1.68	-1.77	-2.66	-1.55	-2.31	-1.88	-2.56	-3.11	-0.85	-1.62	-1.18	-1.58	-1.77	-1.76	-1.65	-0.44	-1.63	-0.96	0.008597
Glu-Ala	-1.85	-1.92	-2.58	-2.65	-3.52	-2.57	-2.78	-2.74	-1.32	-1.87	-1.98	-2.13	-2.27	-2.25	-2.31	-1.91	-1.64	-2.47	-1.81	-2.12	-1.51	-2.63	-2.75	-0.99	-1.72	-1.58	-1.63	-1.82	-1.92	-1.69	-0.85	-2.12	-1.10	0.008599
Pro-Ala	-2.94	-1.85	-2.90	-2.18	-3.08	-3.00	-2.80	-3.38	-1.49	-1.94	-1.94	-2.47	-2.32	-2.01	-2.46	-1.72	-1.89	-2.31	-1.98	-2.18	-1.60	-2.91	-2.95	-1.04	-1.82	-1.79	-1.47	-2.02	-1.69	-1.86	-0.96	-1.91	-0.91	0.008663
Ile-Val	-2.68	-1.92	-2.66	-2.42	-2.67	-2.47	-2.43	-3.37	-1.24	-1.99	-1.99	-2.21	-2.13	-2.26	-2.60	-1.63	-1.48	-2.67	-1.81	-2.06	-1.79	-2.43	-3.20	-1.40	-1.38	-1.25	-1.68	-1.73	-2.02	-1.94	-0.44	-1.19	-0.79	0.008718
Ala-Ser	-2.90	-2.32	-2.60	-1.97	-2.89	-2.43	-2.78	-2.74	-1.63	-2.57	-2.11	-2.78	-2.19	-2.22	-1.94	-1.38	-1.57	-2.11	-1.77	-2.47	-1.73	-2.51	-2.99	-0.83	-1.91	-1.43	-1.33	-1.95	-2.01	-1.49	-0.89	-1.67	-1.34	0.008917
Ser-Phe	-2.51	-2.21	-3.28	-2.48	-3.37	-3.00	-2.80	-3.00	-2.31	-2.37	-2.37	-3.02	-3.05	-2.83	-2.21	-1.94	-2.37	-2.11	-1.86	-1.93	-2.04	-3.38	-3.22	-1.27	-2.40	-1.23	-1.40	-3.31	-1.70	-1.74	-1.30	-1.75	-1.46	0.008930
Malic Acid	-0.86	-1.86	-2.71	-2.53	-3.22	-3.29	-2.72	-3.05	-1.36	-2.22	-2.17	-2.34	-2.03	-2.29	-2.36	-1.42	-1.28	-2.51	-1.71	-2.57	-1.32	-2.80	-2.81	-0.51	-1.16	-1.40	-1.79	-1.43	-1.89	-1.53	-0.49	-0.17	-0.81	0.008982
Ile-His	-3.24	-2.01	-2.70	-1.76	-3.20	-2.62	-2.53	-3.68	-1.19	-1.91	-1.91	-2.55	-2.28	-2.53	-2.76	-1.69	-1.68	-2.54	-1.35	-2.06	-1.81	-2.64	-2.98	-0.30	-1.68	-2.32	-1.67	-1.84	-2.20	-1.72	-0.48	-1.55	-0.85	0.009073
Aspartic Acid	-2.25	-1.94	-2.59	-2.02	-3.05	-2.60	-2.68	-2.61	-1.62	-2.34	-2.02	-2.63	-2.02	-2.12	-2.13	-1.51	-1.56	-1.93	-1.39	-1.99	-1.51	-2.88	-2.66	-0.66	-1.94	-1.99	-1.34	-1.90	-1.65	-1.46	-0.69	-1.80	-1.16	0.009079
Glucose-6-P	-2.32	-0.97	-1.61	-0.70	-3.29	-2.78	-0.78	-2.13	-1.37	-1.56	-1.23	-1.70	-0.46	-0.42	-1.00	-0.69	-0.57	-1.24	-0.35	-1.99	-0.15	-1.05	-2.31	0.48	-0.73	-0.15	-0.07	-1.54	-0.69	-0.41	0.28	0.42	-0.32	0.009128
His-Tyr	-2.60	-1.64	-2.00	-1.73	-3.56	-2.57	-2.36	-2.74	-1.45	-1.98	-1.98	-2.50	-1.56	-1.69	-2.21	-1.55	-1.40	-2.18	-1.56	-2.45	-1.94	-1.94	-2.43	-0.63	-1.59	-1.53	-1.45	-1.40	-1.90	-1.59	-0.51	-1.30	-1.13	0.009300
Val-His	-1.34	-1.65	-2.71	-2.68	-3.34	-2.63	-2.53	-3.44	-1.20	-1.82	-1.82	-2.53	-2.06	-2.00	-1.78	-0.96	-1.68	-2.81	-1.51	-1.63	-1.66	-2.14	-2.34	-0.79	-1.68	-2.63	-1.10	-2.02	-1.28	-1.47	-0.65	-1.47	-0.94	0.009426
Met-Pro	-3.47	-1.83	-2.74	-2.23	-3.38	-2.58	-2.61	-3.72	-1.53	-2.23	-2.23	-2.59	-2.08	-2.63	-2.72	-2.00	-1.89	-2.56	-1.74	-2.83	-1.67	-2.79	-2.89	-1.18	-1.69	-2.34	-1.75	-2.18	-1.86	-1.78	-0.84	-2.11	-0.75	0.009547
Sedoheptulosan	-2.54	-1.62	-2.51	-1.99	-3.34	-2.61	-2.42	-2.36	-1.24	-2.03	-2.08	-2.21	-1.80	-2.06	-2.10	-1.45	-1.37	-2.13	-1.44	-1.94	-1.29	-2.95	-2.68	-0.63	-1.49	-1.32	-1.38	-1.95	-1.84	-1.37	-0.43	-0.26	-1.01	0.009587
Alanine	-2.24	-2.12	-2.70	-1.93	-2.98	-2.48	-2.92	-2.66	-1.28	-2.43	-1.61	-2.53	-1.83	-2.19	-1.96	-1.41	-1.40	-2.25	-1.48	-2.16	-1.56	-2.73	-2.63	-0.70	-1.87	-2.04	-1.32	-1.47	-1.49	-1.63	-0.70	-1.43	-1.12	0.009999
Asp-Val	-2.02	-2.02	-3.01	-2.87	-3.44	-2.52	-2.73	-3.57	-1.31	-2.06	-2.08	-2.36	-2.25	-2.50	-2.63	-4.24	-1.60	-2.98	-1.61	-1.30	-2.57	-2.74	-3.10	-0.59	-1.65	-2.63	-1.73	-1.94	-2.08	-2.14	-0.73	-2.17	-0.85	0.010109
Ala-Thr	-3.27	-2.16	-2.84	-1.71	-2.74	-2.46	-2.61	-2.61	-1.69	-2.58	-2.06	-2.74	-1.81	-1.92	-1.74	-1.36	-1.42	-1.89	-1.58	-2.09	-1.75	-2.72	-2.69	-0.95	-1.75	-1.30	-1.20	-1.75	-1.90	-1.47	-0.76	-2.02	-1.01	0.010713
Phe-Glu	-3.29	-1.97	-3.20	-3.21	-3.40	-2.88	-2.90	-3.80	-2.06	-3.00	-3.00	-2.05	-3.11	-3.02	-3.22	-2.29	-2.56	-3.02	-2.60	-2.99	-1.81	-2.64	-2.95	-1.40	-2.02	-3.10	-2.77	-2.70	-2.79	-2.40	-1.13	-2.28	-1.04	0.011279
Met-Gly	-2.76	-2.19	-2.95	-2.37	-3.52	-2.59	-2.81	-2.91	-1.71	-2.24	-2.24	-2.85	-2.39	-2.36	-2.39	-1.51	-1.67	-1.97	-1.74	-2.56	-2.62	-2.94	-2.81	-0.97	-1.73	-1.92	-1.65	-1.66	-1.77	-1.66	-0.79	-1.86	-1.19	0.011323
His-Lys	-2.51	-1.76	-2.16	-1.88	-2.63	-2.85	-2.61	-2.75	-1.47	-1.97	-1.97	-2.65	-1.77	-2.15	-2.34	-1.27	-1.16	-2.11	-1.35	-2.11	-1.72	-2.24	-2.68	-0.48	-1.67	-1.97	-1.41	-1.37	-1.91	-1.44	-0.37	-1.84	-0.90	0.011430
Val-Leu	-1.46	-1.65	-2.41	-1.41	-2.10	-0.76	-1.30	-2.47	-1.26	-1.83	-1.83	-2.48	-1.92	-2.01	-1.53	-0.86	-1.56	-1.01	-1.18	-1.64	-1.07	-2.73	-0.92	-0.58	-1.80	-0.14	-0.83	-1.00	-1.16	-0.94	-0.56	-1.33	-0.95	0.011784
Pro-Phe	-3.06	-1.95	-2.80	-3.49	-3.64	-2.90	-3.97	-3.82	-1.82	-2.25	-2.25	-2.67	-2.73	-2.86	-2.43	-1.76	-1.70	-2.25	-1.97	-2.59	-2.75	-3.10	-2.89	-1.39	-2.02	-2.63	-1.65	-1.85	-1.52	-1.88	-1.10	-2.38	-1.26	0.011916
Arg-Gln	-0.86	-0.28	-0.51	0.05	-1.89	-1.40	-1.31	-0.90	-0.12	-1.08	-1.01	-1.21	-0.29	-0.54	-0.53	-0.34	-0.66	-1.07	0.45	-0.20	0.01	-1.95	-0.91	1.07	-0.10	-0.08	0.74	-1.00	-1.03	0.16	1.10	-0.13	0.38	0.012237
Ile-Phe	-3.22	-2.12	-2.72	-2.22	-3.32	-2.82	-2.81	-3.80	-1.89	-2.27	-2.27	-3.05	-2.75	-2.75	-2.65	-2.18	-2.30	-2.69	-1.66	-2.79	-2.29	-3.32	-2.78	-0.79	-2.24	-2.23	-2.10	-2.64	-2.39	-2.07	-0.73	-1.60	-1.07	0.012550
Pro-Leu	-1.49	-2.09	-2.30	-1.61	-2.24	-1.79	-1.86	-2.43	-1.21	-2.03	-2.03	-2.49	-1.87	-1.83	-1.76	-0.92	-1.28	-1.09	-1.05	-1.79	-1.15	-3.15	-1.55	-0.50	-1.81	-0.15	-0.93	-1.05	-1.18	-1.44	-0.67	-0.83	-1.18	0.012681
Leu-Asp	-1.43	-2.24	-2.57	-1.82	-3.47	-1.61	-1.66	-2.57	-1.36	-1.98	-1.98	-2.63	-2.00	-2.23	-1.94	-1.11	-1.29	-1.32	-1.38	-1.80	-2.14	-2.63	-2.29	-0.88	-1.71	-0.04	-0.75	-1.15	-1.40	-1.47	-0.80	-0.74	-1.15	0.012789
MethylGlucoside	-2.54	-1.96	-2.72	-2.58	-3.38	-2.86	-1.94	-2.97	-1.37	-2.24	-1.87	-2.53	-1.97	-1.91	-2.24	-1.51	-1.32	-2.54	-1.44	-2.28	-1.65	-2.95	-2.87	-0.60	-1.37	-1.45	-1.74	-1.50	-2.18	-1.68	-0.54	-0.42	-1.60	0.012845
Asp-Gly	-1.86	-2.06	-2.74	-1.78	-3.70	-2.87	-2.89	-2.66	-1.40	-2.36	-2.10	-2.55	-1.93	-1.86	-1.76	-1.67	-1.69	-2.47	-1.61	-1.71	-1.72	-2.65	-2.70	-0.71	-1.95	-1.71	-1.85	-2.02	-1.94	-1.66	-0.81	-1.68	-1.03	0.013063
Lys-Ile	-1.60	-1.51	-1.93	-0.97	-2.40	-0.64	-0.63	-1.86	-1.14	-1.52	-1.52	-2.49	-1.42	-1.35	-1.80	-0.97	-1.39	-1.81	-0.92	-1.15	-1.44	-2.59	-1.74	-0.08	-1.26	0.09	-0.39	-0.60	-1.18	-1.35	-0.07	-0.12	-0.54	0.013243
Lactose	-2.71	-1.77	-2.78	-2.33	-3.17	-2.72	-2.30	-2.77	-1.53	-2.21	-1.86	-2.47	-1.96	-1.78	-2.12	-1.40	-1.44	-2.40	-1.66	-2.15	-1.55	-2.97	-2.56	-0.67	-1.42	-1.77	-1.89	-1.88	-1.85	-1.65	-0.72	-1.09	-1.38	0.013429
Ala-Lys	-2.34	-2.04	-2.68	-2.26	-2.97	-2.51	-2.80	-2.73	-1.61	-2.54	-2.13	-2.59	-2.31	-2.34	-1.94	-1.35	-1.44	-2.11	-1.64	-2.20	-2.17	-2.91	-2.74	-0.76	-1.81	-1.77	-1.40	-1.38	-1.78	-1.64	-0.80	-2.04	-1.29	0.013533
Pro-Gln	-0.14	-0.63	-0.37	0.31	-1.97	-1.14	-0.59	-0.99	0.09	-0.21	-0.21	-0.88	0.07	-0.27	-0.85	-0.35	-1.17	-1.05	0.13	0.22	0.28	-1.58	-0.85	1.07	-0.13	0.43	0.74	-0.56	-0.75	0.10	1.18	-0.04	0.40	0.013587
Adonitol	-2.51	-1.42	-2.44	-1.43	-3.22	-2.63	-2.46	-2.95	-1.31	-2.16	-1.53	-2.46	-1.84	-1.71	-2.03	-1.30	-1.32	-2.00	-1.49	-2.31	-1.07	-2.74	-1.93	-0.33	-1.24	-1.29	-1.23	-2.59	-1.70	-1.38	-0.45	-0.17	-1.01	0.013656
Ile-Arg	-1.42	-1.56	-1.72	-1.18	-2.92	-0.54	-1.04	-1.57	-1.05	-1.73	-1.73	-2.49	-1.74	-1.51	-1.50	-0.94	-1.34	-1.52	-0.86	-1.93	-1.88	-2.59	-1.47	0.09	-1.29	0.48	-0.27	-0.45	-0.92	-1.02	0.22	0.10	-0.56	0.013755
Val-Glu	-1.63	-1.55	-3.18	-2.61	-3.47	-2.45	-2.58	-3.32	-1.02	-2.83	-2.83	-1.90	-2.59	-2.40	-2.47	-2.64	-2.22	-2.92	-2.63	-1.73	-1.45	-2.09	-3.10	-0.82	-1.47	-2.65	-2.33	-2.58	-1.27	-1.80	-0.68	-2.15	-0.68	0.013974
Met-Met	-3.17	-2.13	-3.07	-3.20	-1.55	-2.48	-2.64	-3.56	-1.72	-2.18	-2.18	-2.86	-2.40	-2.45	-2.32	-1.43	-1.59	-2.18	-1.87	-2.91	-2.18	-2.72	-2.33	-1.30	-1.74	-2.38	-1.44	-1.92	-1.78	-1.58	-1.06	-1.74	-1.05	0.014196
Sorbitol	-2.09	-1.43	-2.56	-1.47	-3.04	-2.73	-2.06	-2.11	-1.25	-1.80	-1.60	-2.25	-1.89	-1.43	-2.06	-0.81	-0.89	-2.18	-1.03	-1.73	-0.88	-2.86	-2.73	0.17	-1.11	-1.28	-0.66	-1.66	-1.67	-0.89	0.28	0.21	-1.04	0.014245
Threonine	-2.60	-2.04	-2.47	-1.91	-3.20	-2.44	-2.55	-2.36	-1.24	-2.08	-1.90	-2.37	-1.91	-2.02	-1.66	-1.32	-1.21	-2.15	-1.42	-1.56	-1.61	-2.55	-2.88	-0.58	-1.91	-1.00	-1.36	-1.75	-1.71	-1.63	-0.68	-1.68	-1.01	0.014305
Arg-Ser	-1.13	-2.00	-2.37	-1.85	-3.10	-2.76	-2.87	-2.47	-1.51	-2.56	-1.86	-2.80	-2.14	-2.08	-1.86	-1.36	-1.53	-2.31	-1.51	-1.60	-1.50	-2.64	-2.82	-0.73	-2.02	-0.96	-1.84	-1.50	-1.79	-1.67	-0.69	-1.73	-1.20	0.014464
Dextrin	0.22	0.29	0.46	0.49	0.17	0.15	0.79	0.64	-0.04	-0.41	0.16	-0.66	0.98	1.14	0.76	0.38	0.73	0.56	0.91	0.14	0.87	-0.04	0.92	1.48	0.35	1.31	1.35	0.26	0.49	0.68	1.28	1.32	0.78	0.014798
Glucose	-2.79	-2.08	-2.84	-2.20	-3.37	-2.73	-2.37	-3.10	-1.56	-2.21	-2.00	-2.62	-1.94	-2.12	-2.05	-1.57	-1.43	-2.35	-1.69	-2.45	-1.45	-2.99	-2.38	-0.88	-1.96	-2.23	-1.73	-1.96	-2.01	-1.57	-0.65	-1.12	-1.46	0.015016
Ala-Gln	0.37	-0.30	-0.30	0.41	-1.03	-0.96	-0.57	-0.69	0.16	-0.78	-0.43	-0.79	0.21	-0.11	-0.46	-0.18	-0.11	-0.69	0.36	-0.13	-0.09	-0.22	-0.33	1.27	-0.26	0.45	0.68	-0.33	-0.88	0.16	1.03	-0.19	0.30	0.015105
Thr-Asp	-2.82	-1.81	-2.77	-1.99	-3.51	-2.73	-2.77	-3.16	-1.28	-1.92	-1.92	-2.71	-2.03	-1.95	-2.29	-1.49	-1.45	-2.20	-1.75	-2.01	-1.71	-2.98	-2.75	-0.70	-1.75	-1.80	-1.66	-1.69	-1.60	-1.69	-0.83	-1.87	-0.92	0.015390
Asp-Ala	-2.49	-2.17	-2.86	-2.35	-2.98	-2.68	-2.70	-3.01	-1.60	-2.55	-2.07	-2.86	-2.18	-2.36	-2.17	-1.44	-1.36	-2.15	-1.63	-1.78	-2.10	-2.78	-2.85	-0.89	-2.02	-2.26	-1.78	-1.62	-2.04	-1.77	-0.99	-2.05	-1.30	0.015706
Lactulose	-1.25	-1.78	-2.83	-2.36	-3.47	-2.88	-1.60	-2.59	-1.35	-2.29	-1.92	-2.24	-2.06	-2.02	-2.15	-1.67	-1.49	-2.46	-1.78	-2.45	-1.04	-2.91	-2.60	-0.34	-1.51	-1.11	-1.55	-1.27	-2.01	-1.69	-0.63	-0.69	-1.34	0.015865
Thr-Arg	-2.41	-1.91	-2.67	-2.10	-3.63	-2.60	-2.61	-3.11	-1.62	-2.01	-2.01	-3.02	-2.36	-2.27	-2.40	-1.73	-1.85	-2.09	-1.91	-1.99	-1.71	-3.07	-3.15	-0.82	-2.14	-1.69	-1.63	-1.56	-1.84	-1.94	-0.98	-1.74	-1.34	0.016431
Gly-Ala	-1.89	-2.18	-2.67	-2.21	-2.94	-2.68	-2.63	-3.07	-1.74	-2.40	-2.32	-2.84	-2.18	-2.29	-2.24	-1.43	-1.51	-2.08	-1.84	-2.37	-2.18	-2.77	-2.84	-0.80	-1.86	-1.87	-1.53	-1.54	-1.75	-1.73	-0.91	-1.99	-1.33	0.016473
Lys-Leu	-0.82	-1.50	-2.29	-1.28	-2.20	-1.19	-1.44	-2.16	-1.45	-1.76	-1.76	-2.84	-1.93	-2.12	-1.48	-0.91	-1.24	-0.92	-0.98	-1.84	-1.97	-2.50	-1.02	-0.49	-1.63	0.26	-0.47	-0.42	-1.22	-1.32	-0.37	-0.56	-1.12	0.017086
Pro-Hyp	-2.47	-2.04	-2.46	-1.89	-3.35	-2.59	-2.63	-2.93	-1.42	-1.97	-1.97	-2.53	-1.86	-1.76	-2.24	-1.45	-1.48	-2.17	-1.39	-1.66	-1.89	-2.54	-3.37	-0.68	-1.70	-1.83	-1.38	-1.51	-1.64	-1.62	-0.48	-1.67	-1.00	0.017101
Ile-Gly	-3.51	-2.16	-2.56	-2.24	-3.56	-2.43	-2.47	-3.16	-1.22	-2.05	-2.05	-2.64	-2.01	-2.20	-2.34	-1.56	-1.61	-1.98	-1.59	-1.81	-1.68	-2.83	-3.04	-0.80	-1.83	-2.20	-1.65	-1.43	-2.03	-1.72	-0.89	-1.99	-1.11	0.017293
Asp-Lys	-1.96	-1.97	-2.58	-2.12	-3.34	-2.60	-2.74	-2.70	-1.22	-2.18	-1.95	-2.35	-1.84	-2.02	-2.47	-1.47	-1.80	-2.67	-1.68	-1.87	-1.09	-2.80	-3.00	-0.88	-1.76	-1.93	-1.69	-1.68	-2.09	-1.57	-0.61	-1.81	-0.99	0.017323
Melibionic Acid	-2.55	-1.60	-2.50	-3.11	-3.08	-3.10	-2.56	-3.56	-1.47	-2.32	-1.89	-2.38	-2.12	-2.31	-2.37	-1.57	-1.67	-2.28	-1.68	-2.26	-1.56	-2.59	-2.39	-1.72	-1.57	-1.54	-1.77	-3.52	-1.93	-1.44	-0.86	-0.61	-1.12	0.017497
Gly-Leu	-1.71	-2.01	-2.68	-1.85	-1.95	-2.04	-1.89	-2.50	-1.91	-2.27	-2.27	-2.70	-2.42	-2.22	-1.93	-1.08	-1.53	-1.29	-1.37	-2.18	-2.45	-2.80	-2.09	-0.78	-2.01	-0.27	-0.76	-1.11	-1.38	-1.55	-0.80	-0.97	-1.55	0.017764
Hexanoic Acid	-0.98	-1.61	-2.27	-1.37	-1.55	-1.29	-1.00	-1.89	-1.23	-2.26	-1.65	-2.66	-1.57	-1.62	-1.46	-0.79	-0.93	-0.88	-1.51	-1.66	-1.03	-2.67	-0.23	-0.46	-1.51	0.24	0.21	-1.11	-1.23	-0.99	-0.29	0.02	-1.08	0.018008
Leu-Leu	-0.47	-2.11	-2.56	-1.64	-2.80	-1.38	-1.91	-2.47	-1.52	-2.04	-2.04	-3.06	-2.29	-2.47	-1.71	-0.80	-1.41	-1.07	-1.36	-1.72	-2.29	-2.74	-1.47	-0.63	-1.95	-0.06	-0.76	-0.74	-1.21	-1.40	-0.48	-0.75	-1.28	0.018104
His-His	-2.67	-1.75	-2.63	-2.06	-3.24	-2.75	-2.64	-3.12	-1.34	-2.06	-2.06	-2.34	-1.94	-2.28	-2.31	-1.51	-1.38	-2.53	-1.55	-2.27	-2.16	-2.54	-2.94	-0.56	-1.69	-1.87	-1.59	-1.53	-2.04	-1.36	-0.43	-2.01	-1.02	0.018127
Ala-Pro	-3.15	-1.98	-2.67	-3.21	-3.21	-2.62	-2.79	-3.28	-1.53	-2.14	-2.09	-2.65	-2.09	-2.20	-1.97	-1.79	-1.62	-2.23	-2.09	-2.17	-1.74	-2.63	-2.70	-0.94	-1.98	-2.11	-1.78	-2.62	-2.43	-1.51	-0.99	-1.75	-0.90	0.018351
Leu-Pro	-1.12	-2.05	-2.24	-1.61	-2.02	-2.04	-1.72	-2.25	-1.33	-1.71	-1.71	-2.73	-1.97	-2.05	-1.72	-0.91	-1.19	-1.18	-1.12	-1.80	-2.21	-2.62	-1.77	-0.78	-1.79	0.10	-0.63	-0.49	-1.25	-1.35	-0.57	-0.69	-1.01	0.018725
Val-Asp	-2.40	-2.13	-3.04	-2.15	-3.57	-2.47	-2.66	-3.57	-1.33	-1.65	-1.65	-2.48	-2.08	-1.87	-2.07	-2.45	-1.56	-2.44	-1.55	-1.71	-1.63	-2.52	-3.20	-0.73	-1.78	-2.72	-1.75	-1.99	-1.48	-1.56	-0.64	-1.76	-0.99	0.019209
Met-Lys	-3.31	-1.64	-2.74	-2.48	-3.16	-2.42	-2.63	-3.12	-1.80	-2.10	-2.10	-2.76	-2.27	-2.35	-2.28	-1.63	-1.66	-2.26	-1.86	-2.86	-2.05	-2.86	-2.46	-1.26	-1.80	-2.41	-1.63	-1.97	-1.75	-1.50	-1.30	-1.95	-0.94	0.019909
Melibiose	-3.25	-2.01	-2.49	-2.15	-3.31	-2.90	-2.51	-2.86	-1.38	-2.20	-2.06	-2.57	-1.93	-2.07	-2.11	-1.45	-1.62	-2.34	-1.48	-2.26	-1.54	-2.93	-2.58	-1.19	-1.66	-2.06	-1.42	-2.85	-2.04	-1.60	-0.77	-0.57	-0.97	0.020201
Proline	-3.41	-1.89	-2.54	-1.73	-3.56	-2.54	-2.60	-2.46	-1.28	-2.16	-2.06	-2.47	-1.85	-1.77	-2.11	-1.71	-1.57	-2.37	-1.46	-1.59	-1.12	-2.98	-2.58	-0.78	-1.93	-2.05	-1.54	-1.83	-2.26	-1.69	-0.63	-2.01	-0.77	0.020774
Gly-Ser	-2.43	-1.87	-2.63	-2.41	-3.32	-2.74	-2.84	-2.58	-1.68	-2.29	-2.29	-2.60	-2.21	-2.40	-2.29	-1.33	-1.28	-1.80	-1.58	-2.38	-2.17	-2.51	-3.12	-0.81	-1.92	-1.77	-1.25	-1.51	-1.79	-1.63	-0.93	-1.64	-1.91	0.020842
Phe-Ala	-2.69	-2.65	-3.21	-2.67	-2.33	-2.80	-2.76	-3.35	-2.60	-2.59	-2.59	-3.41	-2.99	-3.10	-2.65	-1.65	-2.24	-2.50	-2.14	-3.02	-3.32	-3.24	-3.05	-1.46	-2.36	-2.31	-1.71	-2.09	-2.02	-1.88	-1.25	-2.01	-1.50	0.021702
Glu-Tyr	-1.57	-1.45	-1.94	-1.77	-2.56	-2.33	-2.17	-2.35	-1.11	-1.46	-1.94	-1.47	-0.99	-1.17	-1.74	-1.67	-1.31	-1.91	-1.58	-1.61	-1.27	-1.28	-1.99	-0.60	-1.11	-1.40	-1.65	-1.79	-1.60	-1.47	-0.42	-1.59	-0.71	0.022317
Leu-Asn	-1.53	-1.99	-2.34	-1.43	-2.82	-1.21	-1.54	-2.28	-1.16	-1.97	-1.97	-2.72	-2.05	-1.95	-1.52	-0.87	-1.14	-1.04	-1.12	-1.73	-1.95	-2.58	-2.02	-0.30	-1.69	-0.09	-0.61	-0.88	-1.21	-1.55	-0.59	-0.67	-1.07	0.022539
His-Met	-2.47	-1.68	-2.56	-2.27	-3.07	-2.75	-2.64	-3.72	-1.75	-2.20	-2.20	-2.81	-2.40	-2.83	-2.31	-1.15	-1.30	-1.77	-1.77	-2.47	-2.32	-2.50	-2.54	-1.04	-1.70	-2.34	-1.66	-1.97	-1.63	-1.45	-0.80	-2.15	-0.98	0.022853
Pro-Pro	-3.17	-1.82	-2.85	-2.44	-3.41	-2.55	-2.81	-3.34	-1.25	-1.86	-1.86	-2.34	-2.12	-2.14	-2.04	-1.67	-1.85	-1.93	-1.79	-1.73	-1.60	-2.78	-3.02	-0.89	-2.01	-2.37	-1.79	-1.99	-1.75	-1.69	-0.79	-1.60	-0.97	0.023042
Glu-Glu	-1.03	-1.43	-2.75	-2.68	-3.52	-2.67	-2.71	-3.16	-1.04	-1.41	-2.64	-1.53	-2.39	-2.56	-2.91	-1.81	-1.57	-2.65	-2.60	-1.20	-0.74	-1.95	-2.76	-0.04	-1.23	-2.47	-1.76	-2.09	-2.61	-1.29	0.18	-1.78	-0.66	0.023920
Ile-Asn	-3.16	-1.58	-2.74	-1.90	-3.20	-2.48	-2.35	-3.12	-0.85	-1.63	-1.63	-1.99	-1.87	-2.52	-2.61	-1.81	-1.50	-2.70	-1.39	-2.46	-1.87	-2.34	-3.01	-0.38	-1.41	-1.45	-1.82	-1.69	-2.05	-1.97	-0.44	-1.30	-0.58	0.023932
Gly-Asn	-1.92	-1.91	-2.47	-2.37	-3.24	-2.78	-2.75	-2.65	-1.37	-2.04	-1.99	-2.56	-1.94	-1.95	-1.70	-1.35	-1.27	-2.13	-1.68	-1.97	-1.81	-2.46	-2.87	-0.65	-1.74	-1.76	-1.53	-1.25	-1.78	-1.61	-0.88	-1.98	-1.17	0.024672
Leu-Gly	-0.72	-2.28	-2.68	-1.83	-2.89	-1.81	-2.08	-2.40	-1.64	-2.25	-2.25	-3.05	-2.31	-2.32	-1.93	-1.04	-1.52	-1.06	-1.45	-2.16	-1.95	-2.90	-1.81	-0.89	-2.07	-0.39	-1.00	-1.12	-1.38	-1.71	-0.93	-1.13	-1.33	0.024697
Ile-Ser	-2.30	-1.84	-2.40	-1.34	-1.03	-2.57	-2.45	-2.92	-1.16	-1.93	-1.93	-2.34	-1.86	-1.75	-1.94	-1.58	-1.69	-2.56	-1.28	-1.49	-2.26	-2.52	-3.13	-0.55	-1.69	-0.19	-1.04	-1.22	-1.76	-1.55	-0.37	-1.06	-0.88	0.024702
Ile-Met	-3.17	-1.95	-2.92	-1.68	-3.25	-2.79	-2.37	-3.63	-1.47	-1.91	-1.91	-2.58	-2.04	-2.34	-2.39	-1.98	-1.77	-2.90	-1.51	-2.51	-2.13	-2.78	-2.66	-0.88	-1.88	-2.37	-1.93	-2.18	-1.85	-1.87	-0.80	-1.97	-0.82	0.024758
Phe-Phe	-2.74	-2.37	-3.32	-2.91	-3.54	-2.92	-2.93	-3.90	-2.43	-2.69	-2.69	-3.15	-3.10	-2.86	-2.86	0.06	-2.62	-2.63	-2.41	-2.67	-2.00	-3.11	-3.20	-1.32	-2.29	-2.67	-2.54	-2.31	-2.00	-1.88	-1.18	-1.65	-1.30	0.024772
Ile-Leu	-1.44	-1.64	-2.47	-1.44	-2.69	-1.45	-1.42	-2.47	-1.02	-1.84	-1.84	-2.29	-1.75	-1.71	-1.56	-0.95	-1.30	-1.30	-1.07	-1.47	-1.60	-2.61	-2.36	-0.57	-1.70	-0.20	-0.76	-1.22	-1.10	-1.37	-0.55	-0.55	-0.81	0.024969
His-Gly	-2.34	-1.87	-2.34	-1.71	-2.88	-2.40	-2.63	-3.17	-1.63	-2.00	-2.00	-2.69	-2.18	-2.30	-2.16	-1.43	-1.40	-2.29	-1.59	-2.34	-2.37	-2.59	-2.91	-0.70	-1.84	-1.72	-1.38	-1.31	-1.82	-1.63	-0.69	-1.66	-1.14	0.025013
Pro-Asn	-2.83	-1.83	-2.76	-1.83	-3.66	-2.83	-2.77	-3.44	-1.19	-1.84	-1.84	-2.35	-1.76	-1.98	-2.32	-1.79	-1.65	-2.42	-1.48	-1.88	-1.55	-2.77	-2.80	-1.62	-1.69	-1.88	-1.78	-1.97	-1.89	-1.97	-0.73	-1.95	-0.85	0.025267
Lys-Met	-2.63	-1.76	-2.77	-1.85	-3.06	-2.54	-2.81	-3.02	-1.64	-2.09	-2.09	-2.85	-2.22	-2.51	-2.26	-1.43	-1.58	-1.88	-1.66	-2.48	-2.18	-2.85	-3.28	-0.78	-1.75	-1.60	-1.40	-1.38	-1.73	-1.56	-0.68	-1.88	-1.07	0.025660
Lys-Tyr	-1.44	-1.61	-1.98	-1.48	-3.70	-1.79	-2.22	-1.86	-1.39	-1.75	-1.75	-2.25	-1.02	-1.14	-1.53	-0.85	-1.18	-1.17	-1.34	-1.99	-1.98	-2.04	-1.97	-0.61	-1.37	0.30	-0.48	-0.54	-1.48	-1.49	-0.25	-0.52	-0.73	0.026138
Glycine	-2.44	-2.19	-2.57	-2.05	-2.94	-2.41	-2.56	-2.29	-1.69	-2.27	-2.11	-2.48	-2.10	-1.98	-1.99	-1.43	-1.47	-2.11	-1.35	-2.28	-1.57	-2.94	-2.76	-0.71	-2.22	-1.76	-1.34	-1.69	-1.67	-1.54	-0.84	-1.82	-1.57	0.026618
Arg-Asp	-3.00	-1.90	-2.53	-2.27	-3.43	-2.45	-2.74	-2.84	-1.34	-2.08	-1.84	-2.40	-1.98	-1.95	-2.73	-1.32	-1.41	-2.55	-1.54	-1.49	-1.39	-2.60	-3.11	-0.42	-1.77	-2.23	-1.66	-2.27	-1.87	-1.64	-0.67	-2.29	-0.85	0.027211
Met-Val	-3.59	-1.98	-3.06	-2.40	-2.94	-2.61	-2.87	-3.76	-1.69	-2.10	-2.10	-2.53	-2.34	-2.47	-2.15	-1.61	-1.56	-2.84	-1.73	-2.45	-2.13	-2.81	-2.92	-1.56	-1.56	-2.68	-1.94	-2.41	-1.64	-1.80	-0.85	-2.21	-1.03	0.028917
Ile-Pro	-3.08	-1.90	-2.60	-1.84	-3.44	-2.51	-2.34	-3.02	-1.15	-1.67	-1.67	-2.16	-1.87	-2.37	-2.18	-1.52	-1.63	-2.39	-1.53	-2.43	-1.95	-2.55	-2.92	-0.37	-1.73	-0.84	-1.42	-2.23	-2.13	-1.68	-0.56	-1.30	-0.85	0.029911

Fucose	-0.61	-1.87	-2.77	-1.93	-3.27	-2.47	-0.87	-2.33	-1.16	-2.20	-1.73	-2.46	-1.99	-2.01	-1.69	-1.35	-1.53	-2.27	-1.29	-1.73	-1.06	-2.82	-2.71	-0.37	-1.39	-0.73	-1.94	-1.71	-1.58	-1.34	-0.27	-0.24	-0.75	0.030699
Val-Asn	-1.10	-2.15	-3.05	-2.47	-3.57	-2.51	-2.66	-3.56	-1.01	-1.46	-1.46	-2.13	-1.60	-1.82	-2.02	-2.48	-1.51	-2.63	-1.54	-1.97	-1.52	-2.39	-2.92	-0.66	-1.47	-2.68	-1.70	-1.73	-1.25	-1.45	-0.57	-1.43	-0.92	0.030851
Gly-Val	-2.95	-2.34	-2.66	-1.82	-2.88	-2.45	-2.69	-3.01	-1.51	-1.91	-1.91	-2.60	-2.06	-2.41	-2.26	-1.99	-1.63	-2.51	-1.82	-2.26	-2.14	-2.79	-2.65	-0.79	-1.99	-2.39	-1.73	-1.74	-1.97	-1.69	-0.78	-2.27	-1.25	0.030872
Gly-Lys	-2.45	-2.08	-2.87	-1.99	-3.16	-2.71	-2.59	-2.73	-1.58	-2.41	-2.41	-2.78	-2.29	-2.31	-2.42	-1.54	-1.61	-2.34	-1.78	-2.86	-2.33	-2.95	-3.04	-1.17	-1.95	-1.97	-1.50	-1.38	-1.81	-1.66	-0.87	-1.86	-1.27	0.032057
Mannan	0.32	-1.94	-2.79	-2.04	-2.29	-2.13	-1.52	-2.44	-1.16	-2.09	-1.51	-2.37	-1.90	-2.02	-2.01	-1.37	-1.52	-1.43	-1.00	-1.54	-1.18	-3.24	-0.80	-0.66	-2.05	-0.20	-0.88	-1.30	-2.02	-1.35	-0.75	-0.20	-1.07	0.033795
Butanone	-2.52	-1.89	-2.51	-2.40	-3.01	-2.37	-1.62	-2.37	-1.40	-2.08	-1.93	-2.51	-1.99	-1.74	-2.16	-1.29	-1.41	-2.34	-1.49	-2.27	-1.27	-2.91	-2.67	-0.50	-1.41	-2.11	-1.49	-2.05	-1.61	-1.43	-0.62	-0.49	-1.65	0.034752
Met-Glu	-3.08	-1.60	-2.99	-2.70	-3.44	-2.70	-2.67	-3.72	-1.31	-2.34	-2.34	-2.20	-2.87	-2.51	-2.82	-2.49	-2.15	-2.88	-2.34	-3.24	-2.13	-2.91	-2.73	-2.02	-1.73	-2.35	-2.21	-2.31	-1.81	-2.43	-0.79	-1.87	-0.87	0.035346
Glu-Gly	-2.09	-1.81	-2.87	-2.70	-3.54	-2.60	-2.82	-3.25	-1.47	-2.01	-2.18	-2.16	-2.41	-2.60	-2.57	-1.63	-1.40	-2.75	-1.98	-2.06	-1.57	-2.47	-3.07	-1.26	-1.70	-2.70	-2.05	-2.08	-2.11	-1.87	-1.16	-2.00	-1.19	0.036291
Ile-Ala	-2.50	-2.01	-2.43	-1.79	-3.21	-2.33	-2.40	-2.62	-1.33	-1.78	-1.78	-2.57	-1.91	-2.03	-2.13	-1.47	-1.70	-1.99	-1.39	-2.16	-2.01	-2.66	-2.90	-0.54	-1.63	-2.83	-1.06	-1.51	-1.66	-1.42	-0.40	-0.94	-0.71	0.036322
Inositol	-2.30	-2.06	-2.39	-2.14	-3.21	-2.90	-1.73	-2.39	-1.38	-2.11	-1.67	-2.42	-1.89	-1.80	-2.19	-1.48	-1.49	-2.23	-1.35	-1.88	-1.30	-2.88	-2.74	-0.40	-1.56	-0.99	-1.45	-3.01	-2.06	-1.40	-0.72	-0.20	-1.30	0.036514
Leu-Tyr	-1.20	-1.82	-1.97	-1.43	-3.63	-1.54	-1.58	-1.94	-1.22	-1.68	-1.68	-2.30	-1.08	-1.14	-1.27	-0.76	-0.96	-0.99	-1.24	-1.75	-1.71	-1.84	-1.23	-0.75	-1.42	-0.14	-0.72	-0.54	-1.41	-1.56	-0.60	-0.86	-1.11	0.037289
Val-Ala	-1.15	-2.33	-2.76	-1.90	-3.06	-2.47	-2.58	-2.88	-1.50	-1.97	-1.97	-2.65	-2.08	-2.10	-1.95	-2.12	-1.62	-1.99	-1.64	-2.33	-1.46	-2.84	-2.91	-0.96	-1.85	-1.98	-1.70	-1.78	-1.69	-1.65	-0.75	-1.47	-1.17	0.037602
Aminoethanol	-2.39	-1.83	-2.90	-2.59	-3.44	-2.58	-1.64	-2.95	-1.02	-1.67	-1.43	-1.98	-1.61	-1.58	-1.78	-1.06	-1.11	-1.81	-1.23	-2.18	-1.50	-2.84	-2.78	-0.19	-1.06	-1.91	-1.77	-1.05	-1.34	-1.02	-0.49	-0.50	-1.10	0.037819
Tween 20	-3.84	-3.35	-3.47	-3.01	-3.51	-2.88	-2.64	-3.74	-3.13	-3.21	-3.02	-3.44	-3.05	-3.84	-3.47	-3.80	-3.34	-3.68	-3.22	-3.27	-3.31	-3.35	-3.37	-3.92	-3.78	-3.38	-3.49	-3.74	-4.88	-4.27	-3.51	-3.49	-4.13	0.038020
Arg-Glu	-2.47	-1.60	-2.58	-2.08	-3.63	-2.55	-2.66	-3.08	-1.10	-1.60	-1.86	-1.75	-1.94	-2.22	-2.76	-1.16	-1.20	-2.62	-1.29	-1.11	-1.22	-1.94	-2.86	-0.20	-1.45	-2.26	-1.65	-2.81	-2.58	-1.44	-0.06	-1.83	-0.72	0.038776
Glu-Val	-1.34	-1.50	-2.32	-2.38	-3.38	-2.54	-2.57	-3.52	-1.66	-1.75	-3.13	-1.54	-2.56	-2.55	-2.53	-2.43	-2.40	-2.61	-2.06	-0.83	-1.42	-2.20	-2.85	-0.54	-1.50	-2.24	-2.34	-2.59	-2.46	-2.58	-0.40	-2.35	-0.81	0.038940
MethylGalactoside	-2.77	-1.89	-2.65	-1.90	-2.97	-2.67	-1.68	-2.24	-1.37	-2.21	-1.77	-2.26	-1.85	-1.98	-1.94	-1.45	-1.59	-2.29	-1.49	-2.48	-1.14	-2.66	-2.69	-0.58	-1.54	-1.14	-1.57	-2.90	-1.75	-1.46	-0.51	-0.46	-1.17	0.039040
Gly-Phe	-2.70	-2.43	-2.99	-2.82	-2.98	-2.73	-2.63	-3.25	-2.40	-2.74	-2.74	-3.29	-2.78	-3.16	-2.58	-1.60	-2.01	-2.26	-1.87	-3.21	-2.92	-3.25	-3.17	-1.54	-2.51	-2.54	-1.88	-1.94	-2.07	-1.89	-1.22	-2.10	-1.54	0.039812
Phe-Asp	-3.28	-2.14	-3.15	-2.86	-3.24	-3.06	-2.79	-3.56	-2.23	-2.51	-2.51	-3.01	-3.02	-3.04	-3.29	-1.92	-2.03	-2.93	-2.34	-3.51	-2.53	-2.94	-2.99	-1.33	-2.04	-2.99	-2.51	-2.70	-2.36	-2.10	-1.25	-2.57	-1.36	0.040879
Pro-Trp	-1.30	-1.52	-1.28	-0.94	-1.60	-1.17	-1.03	-1.45	-0.58	-1.06	-1.06	-1.45	-0.16	-0.34	-0.93	-0.52	-0.50	-1.16	-1.05	-1.45	-0.58	-1.20	-1.01	-0.08	-0.83	0.31	-0.34	-0.22	-0.94	-1.19	-0.15	-0.49	-0.61	0.041660
Asparagine	-2.87	-1.34	-2.47	-2.99	-3.32	-2.87	-3.72	-3.06	-1.14	-1.64	-1.49	-1.91	-1.64	-1.98	-2.15	-1.27	-1.20	-2.02	-1.29	-2.47	-1.39	-2.36	-2.93	-0.61	-1.54	-2.42	-1.91	-1.55	-1.51	-1.30	-0.62	-1.67	-0.73	0.042875
Val-Pro	-1.46	-2.06	-2.79	-2.98	-3.64	-2.51	-2.72	-3.54	-1.16	-2.05	-2.05	-2.27	-2.01	-2.04	-1.83	-1.15	-1.61	-2.86	-1.99	-1.82	-1.50	-2.43	-2.37	-1.01	-1.67	-2.68	-1.93	-2.15	-1.18	-2.74	-0.73	-1.23	-0.97	0.043017
Gelatin	-2.06	-1.76	-1.98	-1.88	-1.95	-2.39	-2.42	-1.79	-1.49	-1.56	-1.64	-1.96	-1.84	-2.15	-1.42	-1.41	-1.71	-1.67	-1.16	-1.81	-1.56	-2.85	-2.74	-0.82	-1.77	-0.92	-0.40	-1.75	-1.87	-1.07	-0.87	-0.62	-0.74	0.043560
Leu-Ala	-1.37	-1.99	-2.65	-1.70	-2.31	-1.56	-1.70	-2.51	-1.67	-2.06	-2.06	-2.84	-2.10	-2.47	-1.90	-0.91	-1.36	-1.06	-1.33	-1.93	-2.44	-2.87	-2.31	-0.74	-1.85	0.04	-0.72	-0.96	-1.27	-1.81	-0.64	-0.81	-1.16	0.043818
His-Ala	-2.11	-2.21	-2.58	-2.01	-0.78	-2.51	-2.48	-2.85	-1.91	-2.46	-2.46	-3.06	-2.72	-2.38	-2.25	-1.45	-1.62	-1.99	-1.66	-2.51	-2.52	-2.72	-2.73	-1.03	-1.94	-1.52	-1.70	-1.44	-1.79	-1.56	-0.74	-1.72	-1.24	0.043829
Val-Phe	-1.17	-2.42	-3.06	-3.20	-3.56	-2.75	-2.63	-4.01	-2.18	-2.38	-2.38	-2.94	-2.85	-2.49	-2.40	-1.19	-2.19	-3.28	-2.08	-1.93	-2.36	-3.13	-2.79	-1.04	-2.15	-3.05	-2.20	-2.97	-1.55	-2.34	-1.02	-1.35	-1.45	0.043887
MethylGlucoside	-1.99	-1.80	-2.74	-2.22	-3.06	-1.64	-1.70	-2.76	-1.15	-2.18	-1.79	-2.21	-1.94	-1.68	-2.06	-1.40	-1.19	-2.32	-1.34	-2.14	-1.24	-2.84	-2.67	-0.53	-1.38	-1.60	-1.56	-1.80	-2.00	-1.51	-0.54	-0.37	-1.26	0.044075
Val-Gln	0.31	-0.84	-1.04	0.16	-2.01	-0.52	-0.68	-1.45	0.16	-0.44	-0.44	-0.61	-0.12	-0.44	-0.49	-0.76	-0.54	-0.38	0.08	-0.49	-0.30	-0.91	-0.80	0.40	-0.42	-0.33	0.37	-0.37	-0.50	-0.19	0.66	-0.29	0.15	0.044403
Tyr-Phe	-1.77	-2.24	-2.35	-2.20	-2.98	-2.31	-2.11	-2.57	-1.52	-1.74	-1.74	-2.43	-1.40	-1.43	-1.75	-2.07	-1.56	-2.06	-1.67	-2.14	-2.25	-2.24	-2.25	-0.98	-1.74	-1.88	-1.66	-1.66	-1.65	-1.79	-0.64	-1.47	-1.18	0.044516
Gly-Gly	-2.79	-2.11	-3.11	-2.05	-3.51	-2.56	-2.79	-2.80	-1.43	-2.27	-2.27	-2.63	-2.19	-2.18	-2.47	-1.57	-1.70	-2.43	-2.03	-2.71	-2.76	-2.73	-3.05	-0.83	-1.95	-2.00	-1.70	-1.69	-1.86	-1.69	-0.94	-1.85	-1.31	0.044623
Glutamic Acid	-2.88	-1.25	-2.77	-2.06	-3.41	-2.60	-2.65	-3.37	-1.98	-2.08	-3.13	-2.13	-2.32	-2.47	-2.50	-2.45	-2.51	-2.82	-1.46	-1.20	-0.81	-2.33	-2.74	-2.60	-1.52	-2.75	-1.99	-2.94	-2.97	-2.93	-0.07	-2.15	-0.72	0.045501
Met-Phe	-3.32	-2.06	-2.99	-2.54	-2.56	-2.91	-2.61	-3.54	-2.28	-2.40	-2.40	-2.94	-2.84	-2.88	-2.81	-2.10	-2.15	-2.64	-2.10	-3.21	-3.37	-2.83	-2.70	-1.47	-2.02	-2.60	-2.06	-2.70	-1.91	-2.01	-1.16	-2.37	-1.27	0.045931
Stachyose	-2.09	-1.65	-2.39	-2.10	-3.01	-2.61	-0.46	-2.11	-1.23	-2.02	-1.69	-2.50	-1.94	-1.81	-2.04	-1.28	-1.38	-2.17	-1.33	-1.84	-1.22	-3.17	-2.59	-0.48	-1.46	-0.78	-1.20	-1.87	-1.69	-1.71	-0.37	-0.10	-0.97	0.046196
Asp-Asp	-3.06	-1.95	-2.86	-2.47	-3.61	-2.85	-2.84	-2.85	-1.58	-2.10	-1.84	-2.45	-1.97	-2.20	-2.26	-1.38	-1.25	-2.02	-1.47	-1.92	-1.38	-2.76	-2.79	-0.72	-1.72	-2.67	-2.72	-1.51	-2.04	-1.75	-0.77	-2.04	-1.20	0.047147
Keto-Glutaric Acid	-3.22	-1.33	-1.88	-1.04	-2.56	-2.56	-0.59	-1.29	-0.51	-1.37	-1.25	-1.63	-1.18	-0.73	-1.71	-0.97	-0.87	-1.54	-0.64	-1.69	-0.49	-1.64	-2.26	0.11	-1.12	-0.70	-0.70	-2.28	-0.89	-1.00	0.17	-0.15	-0.50	0.048046
Phe-Gly	-2.25	-2.47	-2.98	-2.94	-3.38	-2.80	-2.64	-3.44	-2.30	-2.78	-2.78	-3.25	-3.02	-2.61	-2.61	0.12	-2.52	-2.85	-1.87	-2.76	-2.46	-3.37	-3.10	-1.33	-2.47	-2.34	-1.80	-2.05	-1.79	-1.79	-1.37	-2.20	-1.56	0.049065
His-Asp	-2.46	-2.02	-2.12	-2.04	-2.94	-2.56	-2.61	-2.73	-1.37	-1.74	-1.74	-2.54	-1.73	-2.21	-2.09	-1.56	-1.54	-2.40	-1.27	-2.02	-2.16	-2.34	-2.68	-0.35	-1.76	-3.17	-1.22	-1.64	-1.91	-1.44	-0.53	-1.49	-1.16	0.050973
Propanediol	-2.42	-1.88	-2.53	-1.71	-3.25	-3.06	-1.98	-2.75	-1.39	-2.13	-2.01	-2.38	-1.87	-1.96	-1.80	-1.41	-1.46	-2.29	-1.61	-2.05	-1.75	-2.71	-2.87	-0.59	-1.38	-1.81	-1.68	-2.68	-1.89	-1.57	-0.71	-0.49	-1.07	0.051101
Mannitol	0.65	-2.01	-2.58	-1.88	-3.25	-2.35	-1.16	-2.34	-1.41	-2.05	-1.85	-2.22	-1.86	-1.71	-2.21	-1.47	-1.24	-2.24	-1.27	-1.69	-1.07	-2.85	-2.62	-0.31	-1.39	-0.58	-1.37	-1.33	-1.98	-1.28	-0.28	-0.07	-1.05	0.051495
Lys-Phe	-3.20	-1.94	-3.18	-3.35	-3.20	-2.70	-2.84	-3.22	-2.25	-2.51	-2.51	-3.08	-2.89	-2.76	-2.67	-1.79	-1.98	-2.38	-1.93	-3.27	-2.73	-3.25	-3.37	-1.18	-2.16	-2.73	-2.03	-2.29	-2.06	-1.88	-1.05	-2.35	-1.31	0.052167
Lys-Ala	-1.94	-1.91	-2.76	-2.31	-2.07	-2.71	-2.64	-2.97	-1.84	-2.10	-2.10	-2.82	-2.40	-2.56	-2.14	-1.52	-1.76	-2.30	-1.84	-2.87	-2.31	-2.75	-2.93	-1.03	-2.06	-1.70	-1.40	-1.74	-1.94	-1.68	-1.10	-1.78	-1.37	0.052999
Lys-Asp	-2.32	-1.72	-2.73	-1.88	-3.47	-2.51	-2.52	-2.88	-1.23	-1.72	-1.72	-2.53	-1.84	-1.84	-2.31	-1.53	-1.53	-2.34	-1.61	-2.39	-2.31	-2.20	-2.87	-0.52	-1.70	-1.79	-1.65	-1.82	-1.86	-1.67	-0.39	-1.82	-1.00	0.053444
Tagatose	-1.65	-1.61	-2.24	-2.80	-3.00	-2.56	-2.18	-3.10	-1.59	-2.30	-1.97	-2.38	-1.79	-1.93	-1.72	-1.45	-1.48	-2.00	-1.75	-2.21	-1.60	-2.67	-2.18	-1.29	-1.60	-1.60	-1.44	-2.86	-1.95	-1.80	-0.58	-0.34	-1.09	0.053735
Methyl Glucose	-1.97	-1.61	-2.47	-1.79	-2.97	-3.06	-1.78	-3.13	-1.10	-1.86	-1.49	-2.10	-1.71	-1.70	-1.95	-1.17	-1.22	-1.89	-1.13	-2.37	-1.20	-2.87	-2.46	-0.29	-1.40	-1.69	-1.37	-1.88	-1.82	-1.44	-0.39	-0.49	-1.02	0.054361
Fucose	-1.10	-1.81	-2.24	-1.78	-2.87	-2.63	-0.66	-2.05	-1.56	-2.27	-2.00	-2.63	-1.90	-1.65	-1.78	-1.25	-1.53	-2.08	-1.47	-1.95	-0.95	-2.71	-2.70	-0.26	-1.82	-0.94	-0.91	-2.15	-1.66	-1.51	-0.44	-0.42	-1.04	0.055836
Lactic Acid (DL)	-0.23	-1.39	-1.68	-0.95	-1.14	-0.42	-0.72	-1.25	-0.82	-1.74	-1.11	-2.05	-1.10	-1.40	-0.80	-0.07	-0.20	0.05	-0.21	-0.95	-0.28	-2.22	0.01	0.55	-0.94	0.83	0.47	-2.43	-0.36	-0.24	0.15	0.44	-0.50	0.056221
Ile-Tyr	-1.98	-1.55	-1.89	-1.37	-2.77	-2.07	-1.99	-2.37	-0.96	-1.51	-1.51	-1.95	-0.93	-0.87	-1.55	-1.20	-0.99	-2.01	-1.09	-2.51	-1.62	-1.75	-2.24	-0.17	-1.19	-0.27	-0.96	-1.26	-1.74	-1.56	-0.17	-0.65	-0.60	0.056498
Ser-Ala	-2.09	-2.24	-2.47	1.78	-2.68	-2.42	-2.60	-2.64	-1.53	-2.05	-2.05	-2.57	-2.13	-2.16	-1.88	-1.38	-1.45	-1.68	-1.43	-1.64	-1.28	-2.66	-1.64	-0.58	-1.93	-0.57	-0.96	-1.44	-1.62	-1.44	-0.53	-1.33	-1.24	0.057787
Thr-Gln	-0.28	-0.56	-1.36	-0.49	-2.37	-1.58	-1.62	-1.51	-0.17	-0.52	-0.52	-0.64	-0.48	-0.51	-0.84	-0.34	-0.94	-0.97	-0.23	-0.92	-0.46	-1.52	-1.54	0.44	-0.52	-0.66	0.29	-0.61	-0.96	-0.33	0.59	-0.49	0.20	0.059999
Lys-Gly	-2.43	-1.90	-2.60	-1.66	-3.10	-2.40	-2.56	-2.92	-1.49	-2.05	-2.05	-2.73	-2.13	-2.10	-2.06	-1.42	-1.67	-2.38	-1.53	-2.46	-2.50	-2.86	-2.68	-0.83	-1.98	-1.81	-1.56	-1.59	-1.90	-1.72	-0.89	-1.76	-1.38	0.061244
Tyr-His	-0.87	-1.61	-2.08	-2.10	-3.16	-2.37	-2.44	-2.91	-1.52	-1.95	-1.95	-2.66	-1.53	-1.68	-1.40	-1.23	-1.27	-2.11	-1.62	-2.23	-2.06	-2.03	-2.21	-0.70	-1.31	-1.83	-1.45	-1.60	-1.69	-1.50	-0.62	-1.57	-1.01	0.061916
Erythritol	-2.20	-1.87	-2.50	-1.51	-3.32	-2.61	-2.28	-2.64	-1.21	-1.99	-1.65	-2.34	-1.83	-1.82	-1.91	-1.54	-1.51	-2.38	-1.43	-2.56	-1.47	-2.58	-2.84	-0.54	-1.50	-1.72	-1.82	-2.18	-1.92	-1.47	-0.87	-0.52	-1.12	0.064623
Tyr-Val	-1.69	-1.89	-2.15	-2.40	-2.84	-2.06	-2.10	-2.49	-1.26	-1.69	-1.69	-2.12	-0.81	-1.06	-1.36	-1.79	-1.38	-2.20	-1.54	-1.87	-1.49	-2.05	-2.28	-0.67	-1.45	-1.85	-1.71	-1.82	-1.43	-1.57	-0.54	-1.27	-0.87	0.065842
Asn-Val	-1.76	-1.72	-2.62	-2.68	-3.20	-2.54	-2.63	-2.39	-1.29	-1.99	-1.62	-2.29	-1.84	-2.04	-2.04	-1.43	-1.23	-2.48	-1.56	-1.75	-1.54	-2.55	-2.57	-0.32	-1.57	-1.74	-2.71	-1.91	-1.75	-1.69	-0.64	-2.06	-0.97	0.067082
Glycerol	-2.56	-2.12	-2.76	-1.67	-3.32	-2.73	-1.91	-3.11	-1.30	-2.22	-1.78	-2.31	-1.76	-2.06	-1.94	-1.42	-1.33	-2.45	-1.71	-2.36	-1.74	-2.94	-2.83	-0.70	-1.42	-2.56	-1.82	-1.39	-1.87	-1.70	-0.84	-0.44	-1.18	0.067260
Ornithine	-3.04	-2.01	-2.63	-1.84	-3.22	-2.56	-2.81	-2.94	-1.40	-2.22	-1.92	-2.54	-1.86	-1.90	-2.26	-1.55	-1.45	-2.22	-1.57	-2.08	-1.41	-2.57	-2.73	-1.01	-2.01	-2.58	-1.54	-2.64	-2.27	-1.69	-0.63	-1.82	-1.04	0.068055
Arg-Leu	-1.70	-1.89	-2.55	1.33	-2.07	-1.43	-1.68	-2.42	-1.76	-2.47	-1.80	-2.81	-2.44	-2.18	-1.92	-0.84	-1.34	-0.97	-1.33	-1.75	-1.74	-2.74	-1.03	-0.62	-2.03	-0.04	-0.68	-0.79	-1.15	-1.40	-0.78	-0.69	-1.11	0.069070
Glutamine	0.41	-0.30	-0.06	0.67	-0.78	-0.96	-0.29	0.00	0.10	-0.23	-0.26	-0.24	0.45	0.14	0.10	-0.13	0.20	-0.54	0.63	-0.05	0.19	0.01	-0.46	1.18	-0.23	0.46	0.36	-0.04	-0.72	0.11	1.23	0.27	0.42	0.069473
Lys-Glu	-2.92	-1.53	-2.91	-1.35	-2.75	-2.60	-2.56	-3.43	-0.88	-1.79	-1.79	-1.83	-2.05	-2.37	-2.61	-1.66	-1.64	-2.69	-1.30	-2.54	-2.05	-1.92	-2.80	-0.37	-1.58	-2.35	-2.00	-2.42	-1.83	-1.56	-0.34	-1.82	-1.01	0.070432
Sorbose	-1.14	-1.97	-2.54	-3.57	-3.22	-2.63	-1.00	-2.33	-1.37	-2.31	-1.84	-2.58	-1.86	-1.85	-1.74	-1.37	-1.47	-2.16	-1.60	-2.18	-1.41	-2.73	-2.45	-0.49	-1.92	-1.71	-1.30	-2.40	-1.74	-1.69	-0.60	-0.73	-1.11	0.070610
Val-Val	-1.51	-2.16	-3.07	-3.02	-3.24	-2.64	-2.81	-3.57	-1.36	-1.76	-1.76	-2.46	-1.84	-1.86	-1.83	-1.04	-1.42	-2.41	-1.63	-1.63	-1.77	-2.45	-2.27	-0.83	-1.69	-2.74	-1.47	-2.11	-1.37	-2.94	-0.68	-1.77	-1.04	0.071640
Trehalose	-2.10	-1.15	-2.51	-3.05	-3.15	-2.66	-1.05	-2.86	-1.07	-1.94	-1.45	-1.88	-0.71	-1.48	-1.62	-1.03	-0.92	-1.97	-1.30	-2.18	-1.37	-2.53	-2.43	-0.13	-1.13	-2.32	-1.39	-1.06	-1.43	-1.17	-0.14	-0.34	-0.59	0.073523
Homoserine	-2.31	-1.79	-2.37	-1.92	-2.88	-2.58	-2.43	-2.78	-1.45	-2.18	-1.72	-2.43	-1.73	-1.75	-1.64	-1.17	-1.17	-1.74	-1.48	-2.11	-1.65	-2.51	-2.91	-0.72	-1.66	-1.59	-1.47	-1.98	-1.45	-1.58	-1.33	-1.47	-0.92	0.074371
Met-Gln	-0.10	-0.35	-0.61	0.24	-1.93	-1.41	-0.97	-1.07	0.19	-0.32	-0.32	-0.92	-0.32	-0.31	-0.31	-0.33	-1.15	-0.88	0.40	-0.54	-0.85	-0.80	-2.15	1.07	-0.06	0.21	0.36	-0.28	-0.73	-0.03	1.09	-0.06	0.27	0.074840
Gentiobiose	-1.86	-1.80	-2.84	-3.04	-3.08	-2.67	-2.09	-3.10	-1.34	-2.10	-1.93	-2.47	-1.63	-2.08	-1.92	-1.36	-1.59	-2.37	-1.91	-2.34	-1.62	-2.70	-2.71	-0.89	-1.47	-2.56	-2.09	-1.76	-2.03	-1.69	-0.81	-1.09	-1.22	0.075046
Gly-Pro	-2.51	-2.04	-2.74	-2.72	-3.29	-2.64	-2.69	-2.89	-1.33	-1.93	-1.93	-2.64	-1.92	-2.29	-2.17	-1.52	-1.47	-2.08	-1.82	-2.84	-2.14	-2.63	-3.16	-0.85	-1.91	-2.34	-1.56	-1.48	-2.01	-1.68	-0.89	-1.72	-1.01	0.078686
Cyclodextrin	-1.83	-1.99	-2.97	-2.28	-3.38	-2.73	-1.55	-3.25	-1.60	-2.22	-2.20	-2.81	-1.51	-1.98	-2.23	-1.75	-1.63	-2.63	-1.91	-2.56	-1.35	-3.02	-2.51	-0.87	-1.69	-2.45	-2.13	-1.71	-2.37	-1.98	-0.93	-0.88	-1.35	0.079221
Asn-Glu	-1.93	-1.45	-2.63	-2.67	-3.06	-2.56	-2.53	-3.15	-0.89	-1.53	-1.74	-1.73	-1.98	-2.12	-2.82	-1.67	-1.42	-2.73	-1.51	-1.65	-1.57	-2.25	-2.88	-0.05	-1.42	-1.70	-2.85	-2.32	-1.85	-1.57	-0.38	-2.34	-0.74	0.080406
Gly-Ile	-2.70	-2.08	-2.59	-1.89	-3.34	-2.52	-2.42	-3.32	-1.42	-1.95	-1.95	-2.56	-2.08	-2.13	-2.27	-1.70	-1.68	-2.62	-2.02	-2.28	-2.39	-2.74	-3.13	-0.80	-1.78	-2.80	-1.55	-1.58	-2.12	-1.70	-0.84	-1.59	-1.03	0.080436
Raffinose	-2.04	-1.82	-2.59	-2.20	-3.20	-2.53	-0.45	-2.40	-1.24	-2.04	-1.64	-2.40	-1.95	-1.72	-2.03	-1.29	-1.36	-2.29	-1.40	-2.43	-1.33	-2.93	-2.87	-0.21	-1.35	-1.56	-1.16	-1.64	-1.77	-1.36	-0.41	-0.69	-1.45	0.082641
Phe-Met	-2.50	-1.98	-2.93	-2.67	-3.43	-2.89	-2.78	-3.92	-2.24	-2.73	-2.73	-3.04	-3.08	-2.72	-2.61	0.27	-2.10	-2.56	-2.02	-2.84	-2.37	-2.83	-2.79	-1.51	-2.25	-2.98	-2.09	-2.30	-1.99	-1.88	-1.16	-2.07	-1.20	0.086321
Met-Asp	-3.28	-2.08	-2.99	-2.07	-3.27	-2.60	-2.89	-3.34	-1.40	-2.22	-2.22	-2.72	-2.36	-2.36	-2.22	-1.72	-1.87	-2.55	-1.94	-3.27	-2.54	-2.86	-3.18	-0.89	-1.79	-2.81	-1.78	-2.05	-1.84	-1.80	-0.77	-1.86	-0.93	0.086377
Val-Gly	-1.76	-2.28	-2.95	-3.52	-3.44	-2.70	-2.64	-2.58	-1.58	-2.13	-2.13	-2.58	-2.30	-2.20	-1.81	-0.96	-1.80	-2.55	-1.68	-2.38	-2.10	-2.73	-2.69	-1.02	-1.88	-2.63	-1.34	-1.53	-1.58	-1.76	-0.98	-3.25	-1.24	0.088632
Lys-Val	-3.11	-2.10	-2.87	-1.97	-2.28	-2.55	-2.67	-3.47	-1.21	-1.88	-1.88	-2.51	-1.91	-2.10	-1.89	-1.85	-1.73	-2.55	-1.53	-1.94	-2.16	-2.79	-3.07	-0.65	-1.72	-2.45	-2.13	-2.20	-1.71	-1.94	-0.64	-1.95	-1.01	0.088922
Phe-Pro	-2.47	-2.29	-3.13	-3.08	-3.49	-2.69	-2.94	-3.22	-1.89	-2.28	-2.28	-3.06	-2.94	-2.55	-2.81	0.21	-2.10	-2.64	-2.24	-2.55	-1.93	-3.11	-3.11	-1.26	-2.48	-2.44	-2.20	-1.95	-2.01	-1.93	-1.16	-2.03	-1.17	0.089156
MethylMannoside	0.82	-1.06	-1.62	-0.22	-2.13	-0.53	0.11	-1.44	-0.78	-1.39	-1.10	-1.91	-0.81	-0.50	-1.39	-0.69	-0.57	-1.19	-0.13	-0.82	0.02	-1.99	-1.42	0.42	-1.36	0.00	-0.03	-0.44	-1.15	-0.31	0.21	0.71	-0.54	0.089345
Gln-Gln	0.09	-0.44	-0.38	0.43	-0.63	-1.03	-0.11	-0.83	0.29	-0.35	-0.02	-0.31	0.28	0.35	-0.26	-0.03	0.18	-0.30	0.49	0.09	0.28	-0.57	-0.85	1.15	0.02	-0.06	0.72	0.10	-0.85	0.14	1.07	0.16	0.32	0.091717
Ala-Leu	-1.48	-2.04	-2.56	1.58	-2.31	-1.83	-1.84	-2.51	-1.54	-2.59	-2.15	-2.79	-2.18	-2.18	-1.82	-0.87	-1.15	-1.01	-1.22	-2.20	-1.44	-2.82	-1.35	-0.50	-1.83	-0.22	-0.63	-0.65	-1.30	-1.55	-0.73	-0.73	-1.20	0.092807
Uridine	-0.73	-0.87	-1.40	-0.72	-1.74	-1.18	-0.53	-0.78	-0.66	-1.73	-1.14	-1.96	-1.00	-0.53	-1.16	-0.80	-0.60	-1.05	-0.28	-1.87	-0.06	-2.04	-1.94	0.65	-1.06	0.05	-0.99	-0.21	-0.65	-0.71	0.55	0.44	-0.80	0.094609
AcetylMannosamine	0.56	-1.83	-2.83	-2.03	-3.43	-2.47	-2.10	-2.40	-1.33	-2.21	-1.83	-2.40	-1.97	-1.75	-2.50	-1.69	-1.19	-2.40	-1.42	-2.31	-1.27	-2.85	-2.68	-0.53	-1.39	-0.65	-1.62	-2.64	-1.77	-1.34	-0.35	-0.25	-1.29	0.098491
AcetylGlucosamine	-2.34	-1.70	-2.47	-2.10	-3.17	-2.56	-0.44	-2.41	-1.30	-2.11	-1.93	-2.40	-1.74	-1.91	-2.11	-1.26	-1.29	-2.16	-1.35	-2.32	-1.42	-2.72	-2.50	-0.58	-1.34	-2.42	-1.77	-1.38	-1.83	-1.57	-0.56	-0.66	-1.17	0.099048
Valine	-3.01	-2.10	-2.88	-2.40	-3.20	-2.51	-2.84	-3.16	-1.38	-2.10	-2.16	-2.31	-2.10	-2.15	-2.37	-1.56	-1.42	-2.58	-3.64	-1.61	-1.46	-2.40	-3.11	-0.78	-1.67	-2.67	-2.08	-2.22	-1.89	-1.69	-0.70	-2.11	-0.93	0.108410
Ala-Ala	-2.74	-2.15	-3.00	-2.26	-2.92	-2.40	-4.06	-3.02	-1.58	-2.36	-2.24	-2.83	-2.33	-2.30	-2.32	-1.41	-1.29	-1.89	-1.84	-2.56	-3.66	-2.76	-2.90	-1.26	-1.74	-2.25	-1.63	-1.69	-1.73	-1.66	-1.07	-1.98	-1.25	0.109279
Met-Ile	-3.27	-1.94	-2.79	-2.45	-3.52	-2.58	-2.36	-3.61	-1.29	-1.80	-1.80	-2.48	-2.11	-2.29	-2.34	-1.69	-1.38	-2.37	-1.85	-2.93	-2.44	-2.65	-3.01	-0.84	-1.70	-2.43	-2.06	-2.06	-1.80	-1.74	-0.81	-2.05	-0.71	0.111423
Asp-Gln	0.48	-0.64	-0.40	0.39	-2.46	-1.37	-1.13	-0.75	0.10	-1.00	-0.51	-0.94	-0.07	-0.26	-0.31	-0.13	-1.21	-0.97	0.45	-0.25	0.09	-2.14	-0.90	0.75	-0.43	0.02	0.64	-0.89	-1.06	-0.02	0.98	-0.27	0.35	0.111954
Phe-Ile	-2.46	-2.40	-2.94	-2.55	-3.12	-2.57	-2.52	-3.74	-1.73	-2.45	-2.45	-3.02	-2.81	-2.41	-2.98	0.23	-2.15	-2.88	-2.22	-2.18	-2.13	-3.22	-3.21	-2.44	-2.04	-2.27	-1.85	-2.18	-1.99	-1.95	-0.94	-1.81	-0.86	0.112552
Ile-Ile	-3.01	-4.92	-2.89	-2.63	-2.92	-2.51	-2.38	-3.59	-1.10	-1.61	-1.61	-2.10	-1.87	-2.37	-2.55	-1.45	-1.29	-2.11	-2.17	-3.16	-1.54	-2.56	-3.05	-0.97	-1.67	-2.51	-1.97	-1.85	-1.85	-1.77	-0.90	-2.01	-0.91	0.113938
Sucrose	0.51	-1.78	-2.99	-2.32	-3.51	-2.52	-2.37	-2.91	-1.24	-2.40	-2.08	-2.46	-1.99	-1.78	-2.06	-1.38	-1.34	-2.40	-1.63	-1.97	-1.24	-3.01	-2.76	-0.60	-1.68	-2.63	-1.39	-1.08	-2.02	-1.52	-0.52	-0.71	-1.11	0.114131
Mannose	1.07	0.51	0.16	0.84	-0.13	1.01	0.97	0.00	0.20	-0.24	-0.11	-0.69	0.37	0.45	0.42	0.62	0.84	0.97	0.90	-0.10	0.41	0.84	0.85	1.35	0.03	1.26	1.21	-0.19	0.16	0.64	1.38	1.18	0.59	0.114344
Val-Ile	-1.35	-1.99	-3.04	-2.26	-3.25	-2.49	-2.40	-3.59	-1.21	-1.72	-1.72	-2.24	-1.68	-1.56	-1.78	-1.06	-1.98	-2.58	-1.80	-1.56	-1.67	-2.44	-2.47	-0.75	-1.64	-2.47	-1.45	-3.32	-1.38	-1.86	-0.66	-1.29	-0.75	0.118516
Cellobiose	-2.34	-1.88	-3.12	-2.80	-3.35	-2.61	-1.43	-3.28	-1.43	-2.31	-1.50	-2.56	-1.42	-2.16	-2.15	-1.28	-1.44	-2.38	-1.62	-2.50	-1.38	-3.17	-2.92	-0.78	-1.45	-2.69	-2.26	-1.66	-2.11	-1.61	-0.78	-0.61	-1.01	0.121899
Arg-Trp	-1.82	-1.56	-1.25	-1.67	-2.40	-1.68	-1.60	-1.78	-1.01	-1.78	-1.63	-2.07	-0.83	-0.72	-1.54	-1.30	-1.16	-1.83	-1.43	-1.85	-1.12	-1.71	-1.75	-0.29	-1.30	-1.09	-1.35	-1.06	-1.62	-1.73	-0.49	-1.51	-1.05	0.123692
Lys-Ser	-2.38	-1.81	-2.69	-2.26	-3.61	-2.75	-2.64	-3.04	-1.66	-2.10	-2.10	-2.83	-2.27	-2.16	-2.01	-1.41	-1.41	-2.13	-1.69	-2.74	-2.15	-2.81	-3.47	-0.87	-2.06	-1.82	-1.38	-1.47	-1.84	-2.47	-1.06	-1.85	-1.34	0.125605
Lys-Pro	-2.86	-1.80	-2.75	-1.85	-2.98	-2.61	-2.75	-2.95	-1.36	-1.83	-1.83	-2.42	-1.91	-1.91	-2.36	-1.40	-1.37	-2.27	-1.87	-2.40	-1.70	-2.63	-2.97	-0.50	-1.79	-2.18	-1.74	-1.74	-1.91	-2.17	-0.84	-2.22	-0.91	0.127953
Butyric Acid	-0.83	-1.51	-2.02	-1.60	-1.68	-1.38	-0.63	-1.78	-1.24	-1.97	-1.27	-2.34	-1.69	-1.71	-1.26	-0.72	-0.87	-0.62	-0.86	-1.47	-0.77	-2.54	-0.31	-0.21	-1.18	0.49	0.22	-4.13	-0.99	-0.72	-0.03	0.09	-0.82	0.132464
Rhamnose	-1.28	-1.89	-2.64	-2.10	-3.31	-2.47	-1.65	-2.40	-1.37	-2.05	-1.86	-2.43	-1.84	-1.89	-1.80	-1.40	-1.46	-2.40	-1.62	-2.37	-1.54	-3.12	-2.76	-0.78	-1.93	-1.51	-1.29	-2.26	-1.84	-1.72	-0.59	-0.47	-1.02	0.137321
Tyr-Tyr	-1.19	-1.79	-1.92	-1.78	-2.61	-2.15	-1.95	-1.99	-1.30	-1.60	-1.60	-2.02	-0.76	-1.01	-1.30	-3.52	-1.27	-1.99	-1.57	-2.26	-1.61	-1.74	-1.95	-1.30	-1.28	-1.52	-1.41	-1.36	-1.58	-1.69	-0.67	-1.49	-0.96	0.139049
Trp-Phe	-1.00	-1.38	-1.11	-1.10	-1.38	-1.16	-1.16	-1.68	-1.03	-1.63	-1.63	-1.96	-0.80	-0.97	-1.12	-0.94	-0.86	-1.23	-1.24	-1.67	-1.29	-1.25	-0.97	-0.45	-0.99	-0.49	-0.87	-1.52	-1.18	-1.45	-0.58	-0.99	-0.79	0.147102
Methyl Lactate	-2.58	-2.01	-2.73	-2.34	-2.87	-2.77	-2.59	-2.44	-1.38	-2.42	-1.87	-2.43	-1.76	-1.91	-2.03	-1.49	-1.57	-2.23	-1.64	-2.01	-1.39	-3.02	-2.70	-0.62	-1.64	-1.47	-1.97	-4.06	-1.82	-1.69	-0.79	-0.99	-1.29	0.147423
His-Trp	-2.32	-1.30	-0.94	-1.09	-3.54	-1.53	-1.57	-2.01	-0.80	-1.58	-1.58	-1.96	-0.67	-0.83	-1.63	-0.93	-0.58	-1.28	-1.18	-2.11	-1.23	-1.19	-1.24	-0.46	-1.16	-0.93	-1.07	-0.89	-1.57	-1.64	-0.21	-1.44	-0.87	0.148615
Glucose	0.38	0.24	-0.06	0.85	0.29	0.87	0.95	-0.08	0.24	-0.90	-0.14	-0.75	0.20	-0.52	0.47	0.62	0.70	0.84	0.80	-0.55	0.19	0.47	0.78	1.28	-0.15	1.23	1.17	-0.44	0.27	0.54	1.25	1.08	0.15	0.150433
Adenosine	0.74	0.87	1.06	1.24	0.46	0.94	1.12	0.79	0.40	0.04	0.54	-0.24	0.86	0.96	0.77	0.62	0.48	1.09	1.19	0.18	0.84	0.11	1.21	1.42	0.55	1.54	1.43	0.65	0.65	0.87	1.29	1.45	0.57	0.152936
Glucuronic Acid	0.52	-1.92	-2.71	-2.18	-3.22	-2.45	-2.31	-2.61	-1.57	-2.41	-2.03	-2.56	-2.02	-2.04	-2.51	-1.65	-1.43	-2.27	-1.60	-2.95	-1.64	-2.87	-2.54	-1.08	-1.69	-1.15	-2.02	-2.40	-1.85	-1.33	-0.63	-0.51	-0.94	0.153344
Palatinose	0.66	-1.46	-2.27	-2.20	-2.84	-2.19	-2.74	-2.37	-1.23	-2.02	-1.85	-2.34	-1.74	-1.73	-2.43	-1.33	-1.27	-2.19	-1.31	-2.13	-1.39	-2.66	-2.71	-0.36	-1.39	-1.98	-1.69	-1.14	-1.95	-1.51	-0.45	-0.38	-1.06	0.155538
Gly-Thr	-2.40	-1.91	-2.64	-2.22	-3.16	-2.71	-2.82	-3.15	-1.69	-2.06	-2.06	-2.69	-2.13	-2.34	-1.99	-1.58	-1.59	-2.08	-2.00	-2.88	-2.90	-2.74	-2.56	-0.94	-1.97	-2.00	-1.50	-2.60	-1.78	-1.64	-1.13	-2.13	-1.16	0.157404
Xylitol	-0.72	-1.23	-1.83	-0.27	-1.87	-2.34	-0.06	-1.02	-0.81	-1.45	-0.75	-1.80	-0.79	-0.70	-1.29	-0.73	-0.77	-1.15	-0.22	-1.18	-0.97	-1.91	-2.18	0.57	-0.75	0.33	0.08	-2.88	-0.58	-0.35	0.57	0.57	-0.75	0.160744
Glucose	0.26	0.07	-0.27	0.64	0.21	0.93	0.66	-0.04	0.24	0.09	0.09	-0.26	0.43	0.89	0.30	0.62	0.54	0.95	0.36	-0.22	0.44	0.67	0.93	1.04	-0.16	0.98	0.83	0.60	0.17	0.53	0.97	0.84	0.25	0.165656
Tyr-Leu	-1.28	-1.60	-1.78	-1.38	-1.86	-1.51	-1.60	-1.62	-1.39	-1.61	-1.61	-2.15	-0.76	-1.02	-1.30	-0.96	-1.14	-0.86	-1.35	-1.79	-0.93	-1.83	-1.10	-0.84	-1.45	-0.23	-1.00	-2.27	-1.23	-1.40	-0.54	-0.86	-0.95	0.166812
Maltotriose	0.57	0.46	0.27	-0.97	0.81	1.10	1.33	0.79	0.45	-0.11	0.74	-0.20	1.32	1.32	0.80	0.62	0.80	0.71	1.07	0.20	0.62	0.07	1.00	1.50	0.19	1.31	1.36	0.39	0.94	0.70	1.39	1.46	0.78	0.167053
Chondroitin Sulfate	0.60	-1.90	-2.56	-2.11	-3.02	-2.43	-1.84	-2.51	-1.50	-2.39	-2.05	-2.56	-1.93	-1.91	-2.13	-1.56	-1.42	-2.31	-1.85	-2.56	-1.18	-3.21	-2.50	-1.11	-1.95	-0.84	-1.21	-1.85	-1.62	-1.36	-0.91	-0.60	-1.42	0.172189
Leu-Ile	0.03	-1.83	-1.96	-0.97	-1.86	-0.49	-0.82	-1.70	-0.99	-1.35	-1.35	-2.42	-1.62	-1.58	-1.51	-0.92	-1.16	-1.10	-0.73	-1.23	-1.82	-2.47	-1.70	-0.22	-1.61	-0.09	-0.68	-0.63	-1.05	-1.38	-0.25	-0.38	-0.79	0.179387
Leu-Val	-1.28	-1.93	-2.47	-1.45	-2.13	-1.04	-1.83	-2.56	-1.28	-1.83	-1.83	-2.75	-2.05	-2.02	-1.88	-1.02	-1.16	-1.15	-1.27	-1.60	-2.10	-2.85	-1.62	-0.54	-1.67	-0.38	-0.85	-0.90	-1.34	-1.43	-0.66	-0.74	-3.29	0.180943
Met-Tyr	-2.33	-1.59	-1.89	-1.65	-3.16	-2.14	-2.04	-2.05	-1.21	-1.55	-1.55	-2.00	-0.93	-0.98	-1.38	-1.16	-1.09	-1.69	-1.75	-1.19	-1.73	-1.37	-1.77	-0.97	-1.34	-1.86	-1.50	-1.60	-1.79	-1.62	-0.82	-1.77	-1.00	0.181066
Arabinose	-3.17	-1.96	-2.59	1.77	-3.17	-2.65	-2.14	-2.62	-1.56	-2.34	-1.82	-2.58	-2.04	-1.96	-2.16	-1.37	-1.37	-1.98	-1.42	-2.12	-1.25	-2.87	-2.55	-0.62	-1.38	-0.87	-1.38	-2.99	-1.80	-1.36	-0.78	-0.53	-1.24	0.183396
Ile-Trp	-1.66	-1.23	-0.83	-1.05	-2.72	-1.05	-1.01	-1.35	-0.47	-0.96	-0.96	-1.07	0.04	-0.05	-0.90	-0.72	-0.41	-1.09	-0.93	-1.29	-1.06	-0.91	-1.28	0.24	-0.52	-0.54	-0.61	-0.56	-1.25	-1.21	0.08	-0.50	-0.40	0.186078
Tyr-Ile	-0.45	-1.69	-1.85	-1.80	-2.80	-2.02	-2.31	-2.25	-1.05	-1.46	-1.46	-2.04	-1.00	-1.02	-1.44	-1.34	-1.08	-1.92	-1.28	-1.45	-1.55	-1.80	-2.08	-0.92	-1.09	-1.70	-1.35	-1.49	-1.47	-1.59	-0.64	-1.54	-0.77	0.186689
Tween 80	-4.29	-3.11	-3.25	-3.06	-3.34	-2.95	-2.85	-3.70	-3.28	-3.46	-3.15	-3.47	-2.94	-3.61	-3.37	-3.46	-3.21	-3.37	-3.31	-3.38	-3.21	-3.32	-3.02	-3.66	-3.80	-3.41	-3.43	-3.25	-5.01	-3.68	-3.32	-3.31	-3.68	0.188964
Tartaric Acid	-0.73	-1.81	-2.66	1.16	-3.28	-2.82	-2.84	-2.85	-1.64	-2.32	-2.07	-2.69	-2.31	-2.50	-2.62	-1.45	-1.43	-2.25	-1.86	-2.31	-1.55	-3.01	-2.56	-0.85	-1.30	-2.15	-1.62	-1.69	-2.13	-1.53	-0.44	-0.35	-1.37	0.189226
Salicin	-1.94	-2.02	-2.82	-2.23	-3.10	-2.49	-1.15	-2.94	-0.72	-1.43	-1.28	-1.76	-1.87	-1.80	-1.91	-1.43	-1.36	-2.49	-1.63	-2.11	-1.40	-2.74	-2.77	-0.60	-1.71	-1.66	-1.79	-1.50	-2.19	-1.74	-0.60	-0.58	-1.22	0.191309
Phe-Trp	-1.27	-1.41	-1.31	-1.37	-1.55	-1.05	-1.33	-1.82	-1.09	-1.53	-1.53	-1.89	-0.98	-0.99	-1.49	0.32	-0.76	-1.31	-1.34	-1.87	-1.28	-1.35	-0.89	-0.32	-0.92	-0.44	-0.99	-0.71	-1.35	-1.59	-0.34	-1.11	-0.94	0.202397
Amino Butyric Acid	-3.10	-1.39	-2.45	-1.91	-3.27	-2.76	-2.10	-3.47	-1.42	-2.11	-1.79	-2.34	-1.79	-1.80	-2.24	-1.43	-1.62	-2.54	-1.76	-3.47	-1.43	-2.73	-2.71	-0.56	-1.62	-1.54	-1.52	-4.16	-1.97	-1.41	-0.79	-0.27	-0.78	0.205415
Glucose	0.40	0.68	0.05	0.63	0.03	0.70	0.77	0.02	0.21	-0.57	0.01	-0.54	0.39	0.36	0.39	0.76	0.60	0.68	0.47	-0.11	-0.09	0.01	0.58	1.01	-0.17	0.91	0.98	0.89	0.16	0.40	1.09	0.87	0.58	0.206215
Inosine	0.51	0.76	0.98	1.26	0.52	1.06	0.96	0.62	0.42	-0.05	0.54	-0.20	0.87	1.02	0.88	0.75	0.58	1.13	1.09	0.14	1.00	0.03	1.14	1.31	0.50	1.41	1.31	0.30	0.96	0.90	1.26	1.31	0.72	0.207012
Glucosaminic Acid	0.57	-1.72	-2.54	-2.24	-3.20	-2.74	-2.23	-3.00	-1.86	-2.25	-2.14	-2.47	-2.08	-1.91	-2.25	-1.48	-1.43	-2.31	-1.61	-2.87	-1.28	-2.87	-2.51	-0.79	-1.79	-2.13	-1.85	-2.56	-1.85	-1.37	-0.57	-0.84	-1.14	0.224465
Phe-Val	-2.55	-1.93	-2.86	-3.24	-3.47	-2.72	-3.84	-3.84	-2.09	-1.79	-1.79	-2.95	-2.94	-2.85	-2.46	0.30	-1.75	-2.50	-1.88	-2.32	-2.67	-2.87	-3.02	-1.48	-1.96	-2.63	-2.57	-2.59	-1.81	-1.93	-1.17	-2.60	-1.19	0.229931
Tyr-Glu	0.35	-1.55	-2.24	-1.73	-3.12	-2.24	-2.31	-2.74	-1.10	-2.14	-2.14	-1.84	-1.22	-1.55	-2.06	-1.89	-1.60	-2.61	-2.04	-1.54	-1.93	-2.11	-2.31	-0.43	-1.19	-2.13	-1.73	-2.03	-1.73	-1.62	-0.43	-1.63	-0.94	0.229996
Arabinose	-2.67	-1.86	-2.51	-1.36	-3.35	-2.82	-1.64	-2.49	-1.79	-2.21	-1.65	-2.46	-1.79	-1.80	-2.15	-1.44	-1.55	-2.02	-1.43	-2.21	-1.42	-2.61	-2.52	-0.94	-1.28	-1.87	-1.63	-4.35	-1.92	-1.53	-0.65	-0.54	-1.33	0.230105
Trp-Leu	-1.47	-1.49	-1.22	-1.22	-1.89	-0.91	-1.29	-1.66	-0.72	-1.24	-1.24	-1.65	-0.44	-0.63	-1.40	-0.80	-0.57	-1.18	-1.19	-1.75	-1.67	-1.15	-0.64	-0.40	-0.99	-0.07	-0.68	-0.51	-1.35	-1.75	-0.44	-0.89	-1.03	0.235351
Val-Tyr	-1.53	-1.82	-2.11	-2.20	-2.56	-2.16	-2.15	-2.31	-1.27	-1.46	-1.46	-2.13	-1.00	-1.10	-1.50	-0.56	-1.17	-2.16	-1.55	-2.05	-1.67	-1.89	-2.20	-0.77	-1.25	-1.97	-1.42	-1.77	-1.67	-1.23	-0.56	-1.26	-1.07	0.237489
Asp-Phe	-2.63	-2.31	-3.12	1.66	-3.27	-2.90	-2.99	-3.64	-2.10	-2.47	-2.51	-3.01	-2.80	-2.68	-2.71	-2.10	-2.27	-3.12	-2.24	-1.72	-1.61	-3.12	-2.98	-1.18	-2.13	-3.37	-1.86	-2.53	-2.48	-2.04	-1.24	-2.05	-1.27	0.243602
Lys-Lys	-1.99	-1.72	-2.73	-2.13	-2.22	-2.52	-2.70	-2.83	-1.22	-1.78	-1.78	-2.59	-1.80	-1.93	-2.10	-1.51	-1.44	-2.46	-1.49	-2.84	-2.29	-2.74	-2.95	-1.19	-1.78	-1.98	-1.56	-1.58	-2.13	-1.73	-0.53	-1.70	-1.02	0.248300
Lys-Thr	-1.25	-1.37	-1.47	-1.55	-3.13	-1.56	-1.32	-1.93	-0.99	-1.34	-1.34	-1.70	-0.49	-0.58	-1.13	-0.64	-0.58	-0.87	-1.36	-1.88	-1.67	-1.29	-1.12	-0.67	-1.75	-2.04	-1.65	-1.83	-1.71	-1.76	-0.94	-1.80	-1.00	0.259153
Leu-Glu	-0.16	-1.32	-1.67	1.60	-3.21	-0.99	-1.23	-1.70	-0.44	-1.27	-1.27	-1.90	-1.01	-1.52	-1.68	-0.81	-1.31	-0.63	-0.95	-0.85	-1.50	-1.92	-0.60	-0.17	-1.60	-0.09	-0.35	-0.55	-1.02	-1.11	-0.35	-0.55	-0.94	0.259331
Trp-Asp	-1.34	-1.76	-1.36	-1.29	-2.20	-1.33	-1.30	-1.73	-0.93	-1.26	-1.26	-1.90	-0.53	-0.67	-1.13	-1.09	-0.76	-1.76	-1.46	-1.58	-1.53	-1.43	-1.51	-0.31	-0.71	-0.68	-1.04	-0.94	-1.64	-1.54	-0.31	-1.35	-0.92	0.260981
Citric Acid	-2.80	-1.74	-2.43	-1.31	-2.82	-2.37	-2.17	-1.82	-1.34	-1.75	-1.51	-1.78	-1.73	-1.63	-1.88	-1.05	-1.28	-2.11	-1.76	-2.70	-1.38	-2.63	-2.29	-0.72	-1.36	-2.27	-1.81	-1.69	-1.80	-1.47	-0.75	-0.61	-1.11	0.262281
Tyr-Ala	-0.44	-1.97	-2.20	-2.24	-2.71	-1.95	-2.05	-2.22	-1.46	-1.78	-1.78	-2.29	-1.00	-1.12	-1.61	-1.47	-1.48	-1.93	-1.69	-2.08	-1.98	-1.97	-1.89	-0.97	-1.51	-1.57	-1.42	-1.62	-1.82	-1.62	-0.66	-1.57	-1.29	0.265366
Phenylalanine	-3.38	-2.42	-3.17	1.70	-3.54	-2.95	-2.80	-3.41	-2.28	-3.08	-2.81	-3.12	-3.11	-2.90	-2.92	-2.16	-2.30	-2.66	-1.85	-2.51	-2.26	-3.16	-2.99	-2.24	-2.83	-2.59	-2.08	-2.73	-2.22	-2.08	-1.12	-2.22	-1.38	0.272863
Glucose	0.49	0.17	-0.13	0.72	0.28	0.80	0.82	0.20	0.25	-0.51	0.08	-0.95	0.32	0.41	0.51	0.85	0.84	0.94	0.78	-0.39	0.14	0.21	0.83	1.20	-0.25	1.19	1.21	-0.08	0.29	0.52	1.27	1.06	0.14	0.292929
Trp-Val	-0.78	-1.57	-1.30	-2.00	-2.15	-1.13	-1.22	-1.43	-0.57	-0.99	-0.99	-1.56	-0.18	-0.37	-1.27	-1.04	-0.57	-1.49	-1.13	-1.09	-1.12	-1.03	-1.26	-0.18	-0.83	-0.81	-1.02	-1.13	-1.38	-1.58	-0.25	-1.09	-0.81	0.304017
Butanediol	-2.88	-1.95	-2.65	-2.30	-3.47	-2.56	-1.25	-2.92	-1.38	-2.04	-1.92	-2.46	-2.17	-1.99	-2.19	-1.34	-1.47	-2.52	-1.56	-3.11	-1.60	-2.85	-2.95	-0.65	-1.60	-2.03	-1.69	-3.74	-1.75	-1.70	-0.69	-0.77	-1.88	0.305248
Hydroxy-Butyric Acid	-2.67	-1.89	-2.85	-2.30	-3.32	-2.59	-1.75	-3.05	-1.28	-2.20	-1.90	-2.31	-1.81	-1.89	-2.20	-1.44	-1.39	-2.32	-1.80	-2.71	-1.22	-2.83	-2.50	-0.42	-1.90	-1.85	-1.33	-5.21	-1.54	-1.37	-0.63	-0.56	-1.60	0.315695
Phe-Tyr	-1.91	-1.73	-2.16	-2.15	-3.29	-2.33	-2.92	-2.63	-1.45	-1.70	-1.70	-2.34	-1.40	-1.50	-2.63	0.28	-1.38	-2.03	-1.86	-2.42	-1.90	-1.98	-2.04	-1.07	-1.54	-1.95	-1.89	-1.61	-2.02	-1.88	-0.87	-1.86	-0.96	0.317261
Melezitose	0.58	-1.65	-2.58	1.74	-3.29	-2.41	-1.60	-2.56	-1.61	-2.37	-1.83	-2.59	-1.93	-1.94	-2.28	-1.70	-1.29	-2.15	-1.46	-2.19	-1.23	-2.81	-2.79	-0.58	-1.45	-1.06	-1.35	-0.92	-1.93	-1.43	-0.43	-0.30	-1.11	0.318970
Tyr-Gly	-1.26	-1.89	-2.13	-1.73	-2.99	-2.11	-2.09	-2.20	-1.46	-1.91	-1.91	-2.55	-1.05	-1.18	-1.50	-1.33	-1.32	-1.91	-1.63	-2.15	-1.98	-2.62	-2.11	-0.77	-1.54	-1.81	-1.39	-1.59	-1.74	-1.66	-0.74	-1.51	-1.22	0.322517
Trp-Lys	-1.86	-1.64	-1.67	-1.56	-2.40	-1.12	-1.31	-2.00	-0.96	-1.32	-1.32	-1.81	-0.41	-0.55	-1.13	-0.98	-0.71	-1.50	-1.22	-1.93	-1.40	-1.28	-1.34	-0.29	-0.94	-0.76	-1.02	-1.65	-1.39	-1.71	-0.44	-1.32	-0.97	0.322568
Phe-Ser	-2.21	-2.27	-3.04	1.59	-3.59	-2.71	-2.85	-3.25	-2.34	-2.60	-2.60	-3.10	-2.80	-2.80	-2.63	0.18	-2.01	-2.36	-2.04	-2.20	-2.47	-2.91	-2.93	-1.12	-2.24	-1.81	-1.66	-1.75	-1.89	-1.90	-1.31	-1.94	-1.49	0.328355
Tyr-Trp	-1.55	-1.73	-1.48	-1.55	-2.06	-1.65	-1.63	-1.97	-1.16	-1.68	-1.68	-2.24	-1.00	-1.16	-1.25	-1.20	-0.83	-1.42	-1.37	-2.12	-1.61	-1.64	-1.76	-0.53	-1.42	-0.99	-1.32	-1.01	-1.64	-1.97	-0.56	-1.43	-1.21	0.345399
Pro-Gly	-2.56	-2.11	-2.54	1.73	-3.88	-2.73	-2.56	-3.15	-1.38	-2.00	-2.00	-2.57	-1.92	-1.83	-2.13	-1.66	-1.67	-2.34	-1.72	-2.05	-1.94	-2.74	-2.93	-0.73	-1.84	-2.40	-1.56	-1.73	-1.67	-1.67	-0.77	-1.83	-1.14	0.353227
MethyXyloside	-1.85	-1.98	-2.77	-0.61	-3.35	-2.64	-1.48	-2.66	-1.51	-2.23	-1.94	-2.57	-1.88	-1.93	-2.27	-1.53	-1.57	-2.13	-1.79	-2.79	-1.62	-3.27	-2.68	-0.68	-1.43	-1.30	-1.82	-3.16	-2.06	-1.59	-0.80	-0.81	-1.46	0.378542
Leu-Trp	-1.08	-1.54	-1.02	-1.14	-1.89	-0.99	-1.02	-1.32	-0.57	-1.15	-1.15	-1.44	-0.28	-0.37	-1.02	-0.62	-0.51	-0.66	-1.16	-1.45	-0.93	-1.06	-1.04	-0.19	-0.99	0.20	-0.67	-0.21	-1.35	-1.69	-0.40	-0.80	-0.88	0.387547
Acetoacetic Acid	-0.91	-1.61	-2.24	1.19	-2.09	-1.83	-1.51	-1.77	-1.67	-2.41	-1.66	-2.75	-1.33	-1.35	-1.52	-0.76	-1.05	-0.96	-1.19	-1.39	-0.82	-2.21	-0.30	-1.02	-1.60	0.47	-0.24	-5.27	-1.10	-1.03	-0.09	-0.06	-0.93	0.404615
Tricarballylic Acid	-1.21	-1.93	-2.79	-2.72	-3.38	-2.77	-2.84	-3.40	-1.49	-2.32	-1.81	-2.53	-2.28	-2.33	-2.46	-1.77	-1.49	-2.70	-2.09	-3.37	-1.25	-2.86	-2.50	-1.15	-1.77	-2.30	-2.14	-4.16	-2.27	-1.60	-0.80	-2.36	-1.08	0.408673
Trp-Arg	-1.54	-1.63	-1.28	-1.16	-2.22	-1.34	-1.33	-1.83	-1.04	-1.32	-1.32	-1.92	-0.65	-0.69	-1.31	-1.22	-0.92	-1.59	-1.44	-1.87	-1.43	-1.53	-1.49	-0.34	-1.00	-0.76	-1.09	-0.91	-1.62	-1.92	-0.36	-1.42	-1.17	0.423686
Glycogen	0.04	0.51	0.78	0.56	0.08	0.69	1.06	0.35	0.32	0.07	0.46	-0.26	1.21	1.24	0.75	0.73	0.79	0.79	0.89	-0.15	0.07	-0.01	1.02	1.34	0.27	0.78	1.36	0.35	0.92	0.67	1.04	1.31	0.57	0.426064
Glucose	0.32	0.28	0.04	0.99	0.37	0.90	0.75	0.00	0.24	-0.48	0.07	-0.70	0.30	0.40	0.44	0.82	0.99	1.06	0.91	-0.23	0.30	0.42	0.73	1.23	-0.51	1.15	1.13	-0.08	0.12	0.42	1.25	0.88	0.18	0.428964
Ile-Gln	-0.34	-0.30	0.20	0.68	-1.53	0.09	0.28	-0.34	0.50	-0.10	-0.10	-0.83	0.53	0.31	0.04	0.34	-0.31	0.05	0.78	-0.11	-0.40	-1.51	-0.45	1.15	-0.07	0.69	0.89	0.24	-0.56	0.26	1.11	-0.35	0.36	0.430716
Maltitol	-1.95	-1.28	0.51	-2.11	-3.13	-2.50	-1.51	-3.41	-1.33	-2.24	-1.92	-2.22	-1.13	-1.74	-2.26	-1.47	-1.33	-2.33	-1.58	-2.66	-1.32	-2.83	-2.49	-0.70	-1.37	-2.41	-1.87	-1.33	-2.10	-1.45	-0.56	-0.74	-1.15	0.441279
Gly-Tyr	-2.29	-2.01	-2.11	-1.61	-3.41	-2.34	-2.17	-2.21	-1.39	-1.76	-1.76	-2.18	-1.06	-1.13	-1.54	-1.45	-1.28	-1.87	-1.83	-2.54	-2.45	-2.31	-2.27	-0.85	-1.63	-1.74	-1.41	-1.34	-1.81	-1.64	-0.87	-1.85	-1.31	0.457345
Propionic Acid	-2.18	-0.78	-2.89	-3.00	-3.44	-2.36	-2.05	-3.11	-0.69	-1.22	-1.30	-1.27	-1.54	-2.08	-1.43	-0.42	-1.08	-2.34	-1.22	-3.21	-2.42	-1.80	-2.11	-1.13	-1.56	-2.10	-1.71	-2.18	-0.83	-1.89	-0.33	-1.00	-1.11	0.477189
Fructose	1.06	1.16	1.36	1.50	1.03	1.32	1.45	1.10	1.07	0.29	1.09	0.14	1.40	1.52	1.23	1.35	1.57	1.35	1.57	0.55	0.99	1.13	1.49	1.71	0.75	1.62	1.63	0.63	1.27	1.13	1.72	1.57	1.19	0.483382
Succinamic Acid	0.66	0.09	0.38	0.92	0.58	0.92	0.93	0.40	0.98	0.48	0.73	0.40	0.88	1.09	0.66	0.77	1.28	0.84	1.12	0.10	0.84	0.51	1.11	1.24	0.06	0.93	1.04	-1.89	0.47	0.50	1.23	1.00	0.25	0.483641
Trp-Trp	-0.94	-1.56	-0.97	-1.12	-1.69	-0.94	-0.95	-1.24	-0.81	-0.92	-0.92	-1.66	-0.27	-0.53	-0.90	-0.90	-0.56	-1.07	-1.12	-1.43	-1.15	-1.14	-0.89	-0.14	-0.56	-0.50	-0.85	-0.57	-1.27	-1.67	-0.24	-1.14	-0.87	0.511075
Asp-Trp	-1.57	-1.54	-1.14	-1.50	-1.93	-1.51	-1.28	-1.64	-0.68	-1.22	-1.17	-1.53	-0.25	-0.40	-1.15	-1.11	-0.74	-1.47	-1.49	-1.60	-1.02	-1.20	-1.24	-0.30	-0.75	-0.69	-1.21	-0.77	-1.81	-1.77	-0.51	-1.40	-0.91	0.525736
Maltose	0.98	0.40	0.18	1.36	1.07	1.35	1.40	1.06	0.95	0.23	0.89	0.08	1.43	1.44	1.01	1.11	1.25	1.16	1.06	0.45	0.91	0.48	1.31	1.60	0.49	1.44	1.53	0.63	0.82	0.98	1.56	1.53	0.95	0.576629
Trp-Gly	-1.33	-1.63	-1.21	-0.98	-1.94	-1.16	-1.22	-1.41	-0.68	-1.12	-1.12	-1.51	-0.29	-0.45	-1.00	-0.89	-0.68	-1.21	-1.27	-1.62	-1.35	-1.17	-1.31	-0.24	-0.70	-0.60	-0.82	-0.54	-1.41	-1.50	-0.53	-1.22	-1.06	0.589857
Ala-Tyr	-2.13	-1.89	-2.28	1.75	-2.80	-2.12	-2.13	-2.34	-1.41	-2.01	-1.85	-2.10	-1.15	-1.15	-1.60	-1.29	-1.34	-1.91	-1.71	-2.09	-1.79	-2.09	-2.28	-0.94	-1.34	-1.06	-1.12	-1.40	-1.74	-1.50	-0.61	-1.81	-1.21	0.591199
Methyl Succinate	0.88	0.51	0.73	1.12	0.63	1.09	1.27	0.69	1.13	0.73	0.83	0.60	1.09	1.21	0.93	1.01	1.37	0.90	1.30	0.29	0.93	0.68	1.22	1.41	0.30	1.16	1.22	-0.24	0.69	0.72	1.44	1.27	0.54	0.637598
Turanose	0.69	-0.36	1.24	-1.67	-2.61	-2.36	0.13	-2.17	-0.82	-1.51	-1.23	-1.72	-1.11	-0.92	-1.63	-0.89	-0.71	-1.76	-0.71	-1.52	-0.70	-1.51	-2.38	0.22	-0.84	-1.40	-0.93	-1.33	-1.24	-0.83	-0.03	0.08	-0.99	0.647552
Hydroxy-Butyric Acid	-0.32	-1.82	-1.69	-0.22	-1.24	-1.79	0.16	-1.28	-0.32	-1.63	-0.80	-1.78	-1.30	-0.64	-0.56	0.07	-0.01	-0.09	0.31	-0.72	-0.20	-2.73	-1.41	0.69	-0.89	0.33	0.34	-5.38	-0.18	-0.42	0.52	0.51	-0.41	0.648925
Keto-Butyric Acid	-0.52	-2.23	-2.47	-0.99	-2.47	-0.87	-1.09	-2.63	-1.59	-2.67	-2.06	-2.71	-1.99	-1.59	-2.02	-1.43	-1.24	-1.63	-1.86	-1.22	-1.55	-2.50	-2.43	-0.96	-2.82	-0.94	-1.43	-5.88	-1.93	-2.34	-0.76	-0.89	-1.83	0.650408
Tween 40	-4.13	-3.28	-3.72	-3.10	-3.70	-2.95	-3.07	-3.74	-3.32	-3.43	-3.16	-3.80	-3.12	-3.68	-3.34	-3.90	-3.46	-3.68	-3.40	-3.32	-3.38	-3.27	-3.07	-3.28	-3.49	-3.21	-3.49	-3.41	-4.01	-3.78	-3.41	-3.51	-3.54	0.695335
Trp-Tyr	-0.45	-1.45	-1.01	-1.42	-2.01	-1.27	-1.37	-1.46	-0.83	-1.28	-1.28	-1.75	-0.24	-0.54	-0.95	-1.12	-0.68	-1.43	-1.26	-1.75	-1.22	-1.01	-1.25	-0.43	-0.86	-0.80	-1.06	-0.96	-1.52	-1.67	-0.35	-1.23	-0.89	0.705451
Trp-Glu	-1.29	-1.40	-1.01	-1.17	-1.93	-1.13	-1.14	-1.39	-0.53	-0.97	-0.97	-1.43	-0.31	-0.45	-1.18	-1.30	-0.89	-1.77	-1.78	-1.53	-1.32	-1.04	-1.48	-0.50	-0.69	-0.82	-1.35	-1.11	-1.72	-1.69	-0.27	-1.60	-0.84	0.718796
Pro-Tyr	-2.72	-1.81	-2.11	1.75	-2.75	-1.95	-2.29	-2.31	-1.20	-1.50	-1.50	-2.12	-0.97	-1.02	-1.53	-1.47	-1.28	-2.13	-1.71	-1.63	-1.14	-2.10	-2.10	-0.71	-1.45	-1.86	-1.43	-1.61	-2.11	-1.67	-0.54	-2.04	-0.79	0.769062
Glycerol Phosphate	-1.17	-0.20	-0.15	1.35	-1.31	-0.75	0.17	-1.01	-0.33	-1.01	-0.92	-1.17	-0.57	-0.16	-0.34	-0.72	-0.49	-1.03	-0.51	-1.11	-0.89	-1.17	-1.14	0.21	-0.78	-0.50	-0.47	-0.36	-1.17	-0.87	0.30	0.14	-0.67	0.785345
Tryptophan	-0.99	-1.23	-0.61	-1.13	-1.53	-0.89	-0.86	-1.12	-0.29	-0.83	-0.69	-0.98	0.33	0.18	-0.66	-0.71	-0.28	-0.94	-1.04	-1.33	-0.90	-0.66	-1.08	-0.11	-0.52	-0.22	-0.68	-0.25	-1.22	-1.48	-0.26	-1.02	-0.90	0.787319
Ala-Trp	-1.68	-1.52	-1.05	-1.08	-1.86	-1.40	-1.33	-1.37	-0.72	-1.40	-1.03	-1.46	-0.15	-0.31	-1.00	-0.86	-0.58	-1.28	-1.35	-1.71	-1.16	-1.13	-1.34	-0.36	-0.95	-0.68	-0.81	-0.98	-1.52	-1.38	-0.41	-1.33	-1.10	0.814605
Trp-Ser	0.10	-1.61	-1.34	-1.06	-2.02	-1.21	-1.25	-1.45	-0.82	-1.20	-1.20	-1.93	-0.25	-0.43	-0.85	-0.96	-0.71	-1.35	-1.23	-1.61	-1.28	-1.15	-1.41	-0.33	-0.88	-0.56	-0.81	-0.83	-1.30	-1.56	-0.60	-1.19	-1.08	0.848801
Gly-Trp	-1.79	-1.59	-1.10	-1.11	-3.05	-1.21	-1.18	-1.51	-0.74	-1.19	-1.19	-1.40	-0.13	-0.31	-1.02	-0.89	-0.46	-1.16	-1.40	-1.93	-1.38	-1.11	-1.34	-0.31	-0.98	-0.82	-1.05	-0.77	-1.39	-1.47	-0.55	-1.43	-1.08	0.853004
Pyruvic Acid	-0.12	-1.55	-0.93	0.09	-1.29	-0.38	-0.16	-1.06	-0.23	-1.42	-1.05	-1.55	-0.82	-0.69	-1.18	-1.28	-0.54	-0.45	-1.05	-0.97	-1.14	-1.87	0.39	-0.14	-1.56	0.08	0.05	-2.44	-1.42	-1.31	-0.19	-0.35	-0.91	0.858587
Met-Trp	-1.58	-1.22	-1.09	-1.27	-0.63	-1.05	-1.07	-1.50	-0.65	-1.06	-1.06	-1.40	-0.09	-0.20	-0.91	-0.66	-0.36	-0.92	-1.16	-1.61	-1.20	-0.83	-0.69	-0.38	-0.83	-0.60	-1.01	-0.68	-1.32	-1.47	-0.50	-1.11	-0.87	0.874212
Trp-Ala	-1.23	-1.60	-1.15	-1.01	-1.99	-1.04	-1.09	-1.40	-0.76	-1.07	-1.07	-1.59	-0.22	-0.36	-1.04	-0.87	-0.67	-1.34	-1.69	-1.64	-1.15	-1.27	-1.06	-0.42	-2.05	-0.41	-0.75	-0.55	-1.39	-1.69	-0.37	-1.17	-0.93	0.910456
Butyric Acid	-2.02	-2.10	-2.77	-3.18	-3.11	-2.69	-2.15	-2.91	-2.74	-3.24	-3.11	-3.59	-2.51	-2.67	-2.37	-1.52	-2.24	-2.85	-2.58	-2.81	-2.84	-3.34	-1.58	-2.22	-3.70	-2.55	-2.42	-3.97	-2.76	-3.25	-1.73	-1.95	-2.44	0.918654
Glu-Trp	-1.19	-1.40	-1.08	-1.12	-1.55	-1.16	-1.04	-1.74	-0.40	-0.92	-0.95	-1.07	-0.03	-0.22	-1.04	-1.06	-0.36	-1.10	-1.37	-1.09	-1.07	-0.84	-1.16	-0.21	-0.63	-0.70	-1.16	-0.73	-1.67	-1.43	-0.38	-1.38	-0.86	0.945239
Galactose	0.44	0.45	0.56	0.91	-0.06	1.13	0.97	0.42	0.32	-0.69	-0.04	-1.14	0.64	0.71	0.41	0.49	0.30	0.37	0.75	-0.23	0.57	-1.53	1.05	1.14	-0.29	1.04	1.21	-1.17	0.42	0.39	0.63	1.12	0.30	0.949686
Glucose	0.37	0.37	-0.04	0.59	0.03	0.88	0.70	-0.57	0.16	-0.03	-0.03	-0.41	0.44	0.39	0.41	0.75	0.65	0.74	0.35	-0.74	-0.98	0.43	0.48	0.91	-0.29	0.86	0.94	0.73	0.01	0.19	0.98	0.59	0.03	0.997130
