# Supplementary material for: Decreased tryptophan metabolism in patients with autism spectrum disorders
Source: Mol Autism. 2013 Jun 3;4:16. doi: 10.1186/2040-2392-4-16 (PMC3680090; doi:10.1186/2040-2392-4-16)
Supplement: Additional file 4: Table S4 — Significant absorbance data of PM-M4 wells for 20 controls (C1-C20) and 20 patients with ASDs (A1-A20). Notes. The data were log-transformed before undergoing statistical analyses. The wells containing tryptophan are indicated in red. [file 2040-2392-4-16-S4.pdf]

Additional Table 4. Significant absorbance data of PM-M4 wells for 20 controls (C1-C20) and 20 patients with ASDs (A1-A20).

**Substrate**	C1	C2	C3	C4	C5	C6	C7	C8	C9	C10	C11	C12	C13	C14	C15	C16	C17	C18	C19	C20	A1	A2	A3	A4	A5	A6	A7	A8	A9	A10	A11	A12	A13	A14	A15	A16	A17	A18	A19	A20	*P* value
Phe-Met	0.14	0.16	0.11	0.14	0.13	0.15	0.15	0.18	0.15	0.15	0.14	0.17	0.13	0.12	0.16	0.13	0.13	0.19	0.20	0.18	0.20	0.18	0.34	0.18	0.23	0.25	0.15	0.14	0.10	0.18	0.42	0.16	0.28	0.13	0.14	0.17	0.18	0.20	0.17	0.19	0.0103
Trp-Asp	0.62	0.69	0.35	0.40	0.67	0.66	0.65	0.55	0.91	1.23	1.05	0.62	0.28	0.55	0.37	0.58	0.52	0.65	0.78	0.89	0.65	0.47	0.53	0.44	0.28	0.70	0.38	0.46	0.69	0.58	0.45	0.53	0.52	0.17	0.44	0.44	0.61	0.67	0.38	0.35	0.0122
Trp-Ala	0.65	0.62	0.40	0.45	0.68	0.80	0.63	0.64	1.01	1.45	1.21	0.73	0.35	0.63	0.47	0.62	0.59	0.71	0.88	1.00	0.82	0.57	0.57	0.50	0.34	0.83	0.45	0.60	0.72	0.59	0.39	0.60	0.55	0.23	0.64	0.47	0.58	0.52	0.46	0.44	0.0137
Trp-Trp	0.76	0.77	0.39	0.43	0.62	0.88	0.76	0.72	1.07	1.52	1.19	0.75	0.34	0.67	0.52	0.73	0.66	0.84	0.89	1.20	0.95	0.57	0.61	0.51	0.36	0.86	0.37	0.63	0.74	0.64	0.44	0.66	0.55	0.25	0.69	0.49	0.77	0.81	0.46	0.40	0.0144
Trp-Ser	0.59	0.60	0.31	0.34	0.57	0.69	0.58	0.58	0.83	1.12	1.04	0.61	0.33	0.51	0.44	0.56	0.52	0.63	0.75	0.82	0.69	0.44	0.46	0.42	0.28	0.68	0.40	0.48	0.59	0.51	0.42	0.49	0.48	0.21	0.56	0.42	0.65	0.67	0.40	0.37	0.0163
Trp-Arg	0.54	0.54	0.31	0.22	0.61	0.61	0.54	0.47	0.71	1.05	0.88	0.49	0.27	0.43	0.33	0.47	0.46	0.66	0.73	0.72	0.59	0.40	0.46	0.38	0.25	0.57	0.30	0.43	0.56	0.41	0.40	0.50	0.46	0.17	0.43	0.38	0.52	0.57	0.33	0.31	0.0164
Trp-Tyr	0.59	0.61	0.31	0.38	0.55	0.66	0.59	0.51	0.81	1.09	0.93	0.60	0.29	0.51	0.40	0.54	0.61	0.60	0.74	0.82	0.59	0.46	0.48	0.48	0.30	0.66	0.37	0.49	0.54	0.47	0.40	0.48	0.51	0.22	0.51	0.44	0.65	0.68	0.41	0.39	0.0177
Trp-Gly	0.58	0.63	0.38	0.36	0.61	0.71	0.64	0.58	0.87	1.22	1.14	0.67	0.32	0.60	0.42	0.58	0.56	0.67	0.83	0.85	0.71	0.52	0.52	0.43	0.29	0.81	0.38	0.51	0.67	0.51	0.43	0.52	0.53	0.18	0.51	0.46	0.65	0.70	0.39	0.41	0.0179
Trp-Val	0.65	0.63	0.35	0.38	0.60	0.64	0.63	0.57	0.84	1.21	1.01	0.58	0.32	0.52	0.39	0.55	0.62	0.66	0.81	0.88	0.72	0.48	0.52	0.44	0.34	0.70	0.38	0.51	0.58	0.51	0.49	0.52	0.52	0.20	0.53	0.44	0.70	0.69	0.38	0.36	0.0211
Trp-Glu	0.59	0.59	0.33	0.41	0.70	0.62	0.64	0.52	0.81	1.10	0.98	0.55	0.29	0.54	0.38	0.55	0.49	0.63	0.69	0.87	0.63	0.49	0.51	0.40	0.28	0.74	0.35	0.48	0.61	0.51	0.49	0.50	0.53	0.18	0.50	0.40	0.65	0.64	0.36	0.34	0.0222
Trp-Leu	0.55	0.68	0.40	0.38	0.58	0.79	0.62	0.58	0.80	1.13	1.05	0.63	0.34	0.56	0.48	0.61	0.55	0.83	0.74	0.86	0.71	0.49	0.54	0.51	0.35	0.74	0.44	0.47	0.67	0.54	0.43	0.61	0.53	0.11	0.45	0.63	0.61	0.69	0.56	0.44	0.0225
Trp-Lys	0.68	0.58	0.32	0.33	0.53	0.66	0.67	0.55	0.87	1.14	1.03	0.55	0.29	0.50	0.37	0.51	0.50	0.64	0.72	0.82	0.67	0.47	0.45	0.40	0.31	0.65	0.38	0.49	0.60	0.55	0.42	0.47	0.49	0.19	0.52	0.42	0.63	0.65	0.41	0.35	0.0260
Trp-Phe	0.68	0.55	0.38	0.35	0.54	0.70	0.63	0.48	0.71	0.93	0.85	0.58	0.37	0.49	0.44	0.55	0.50	0.62	0.69	0.72	0.65	0.40	0.51	0.48	0.33	0.57	0.41	0.58	0.60	0.54	0.46	0.60	0.52	0.22	0.45	0.49	0.57	0.63	0.46	0.41	0.0281
Phe-Phe	0.16	0.14	0.11	0.12	0.13	0.16	0.13	0.15	0.14	0.17	0.16	0.16	0.13	0.14	0.18	0.14	0.15	0.19	0.24	0.19	0.22	0.16	0.28	0.17	0.18	0.17	0.16	0.15	0.13	0.16	0.33	0.16	0.22	0.12	0.12	0.16	0.20	0.20	0.20	0.19	0.0337
Phe-Val	0.23	0.14	0.11	0.13	0.14	0.17	0.14	0.17	0.15	0.19	0.17	0.16	0.17	0.13	0.13	0.15	0.17	0.17	0.21	0.18	0.26	0.19	0.37	0.20	0.20	0.15	0.15	0.15	0.15	0.14	0.43	0.14	0.27	0.10	0.11	0.16	0.21	0.23	0.18	0.19	0.0514
