# Supplementary material for: Decreased tryptophan metabolism in patients with autism spectrum disorders
Source: Mol Autism. 2013 Jun 3;4:16. doi: 10.1186/2040-2392-4-16 (PMC3680090; doi:10.1186/2040-2392-4-16)
Supplement: Additional file 5: Table S5 — Expression microarray data for genes involved in tryptophan metabolic pathways in 10 patients with ASDs versus 10 controls. Notes. Each cell line from the 10 patients with ASDs was individually compared to the control group using the Mann–Whitney one sample test. Bold digits denote that control intensities are well above detection threshold, red color denotes that the gene is expressed in the patient significantly more than in controls, blue color denotes that the gene is expressed in the patient significantly less than in controls. [file 2040-2392-4-16-S5.pdf]

Additional Table 5. Expression microarray data for genes involved in tryptophan metabolic pathways in 10 patients with ASDs versus 10 controls.

Gene Symbol	Control Average	AUT1	AUT2	AUT3	AUT4	AUT5	AUT6	AUT7	AUT8	AUT9	AUT10
AADAT	100	33	29	40	36	36	69	37	54	52	26
ACMSD	29	17	33	40	24	23	28	17	34	15	6
HAAO	1201	1159	455	285	743	566	992	641	697	918	374
KMO	1167	1890	1217	763	646	629	1311	896	705	723	288
KYNU	9312	12141	9682	3832	5647	4633	10400	8417	7019	9868	4566
MAOA	781	422	437	246	394	247	567	354	411	542	153
QPRT	51099	120513	89478	41459	76330	59482	68206	45937	62899	68640	30829
TDO2	43	250	118	50	28	36	38	14	29	40	45
TPH1	35	27	33	41	27	29	27	15	36	15	9
TPH2	58	22	43	35	28	35	32	22	41	31	11
WARS (cytoplasmic form)	60518	68775	81421	64014	64490	50202	55002	33855	43543	42147	22084
WARS2 (mitochondrial form)	2207	2812	1707	630	790	689	1250	1387	1717	2250	977
SLC3A2	24016	20724	32092	18967	20111	13763	21640	20804	24236	25123	8286
SLC7A5	4513	1641	4189	4972	3944	1595	3028	2382	3313	2782	905
SLC7A8	95	22	19	43	29	23	38	18	29	48	9
